# Carbon–Phosphorus Coupling from C^N Cyclometalated Au^III^ Complexes

**DOI:** 10.1002/chem.201905392

**Published:** 2020-03-06

**Authors:** Riccardo Bonsignore, Sophie R. Thomas, Wim T. Klooster, Simon J. Coles, Robert L. Jenkins, Didier Bourissou, Giampaolo Barone, Angela Casini

**Affiliations:** ^1^ School of Chemistry Cardiff University Main Building, Park Place CF10 3AT Cardiff UK; ^2^ School of Chemistry University of Southampton Southampton SO17 1BJ UK; ^3^ CNRS/Université Paul Sabatier Laboratoire Hétérochimie Fondamentale et Appliquée (LHFA, UMR 5069) 118 Route de Narbonne 31062 Toulouse Cedex 09 France; ^4^ Dipartimento di Scienze e Tecnologie Biologiche, Chimiche e Farmaceutiche Università degli Studi di Palermo Viale delle Scienze, Edificio 17 90128 Palermo Italy; ^5^ Department of Chemistry Technical University of Munich Lichtenbergstr. 4 85747 Garching Germany

**Keywords:** carbon–phosphorous bond, cross-coupling, density functional calculations, gold(III) cyclometalated compounds, reductive elimination

## Abstract

With the aim of exploiting new organometallic species for cross‐coupling reactions, we report here on the Au^III^‐mediated C_aryl_−P bond formation occurring upon reaction of C^N cyclometalated Au^III^ complexes with phosphines. The [Au(C^N)Cl_2_] complex **1** featuring the bidentate 2‐benzoylpyridine (C^CO^N) scaffold was found to react with PTA (1,3,5‐triaza‐7‐phosphaadamantane) under mild conditions, including in water, to afford the corresponding phosphonium **5** through C−P reductive elimination. A mechanism is proposed for the title reaction based on in situ ^31^P{^1^H} NMR and HR‐ESI‐MS analyses combined with DFT calculations. The C−P coupling has been generalized to other C^N cyclometalated Au^III^ complexes and other tertiary phosphines. Overall, this work provides new insights into the reactivity of cyclometalated Au^III^ compounds and establishes initial structure–activity relationships to develop Au^III^‐mediated C−P cross‐coupling reactions.

## Graphical abstract

**Go for P**: C^N cyclometalated Au^III^ complexes were discovered to readily react with phosphines under mild conditions to afford phosphoniums. NMR spectroscopy, mass spectrometry and DFT calculations provide insight into this rare Au^III^‐mediated C−P cross‐coupling reactions.

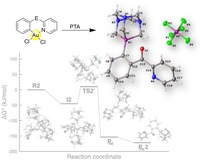

Gold homogeneous catalysis is a thriving field with numerous novel and unexpected discoveries every year.^1^ Homogenous gold catalysed transformations present several key features, including high atom economy, high functional group tolerance, orthogonal reactivities compared to other transition metal catalysts, as well as an important increase in molecular complexity. In contrast to the well explored Au^I^ catalysts, only limited studies exist on Au^III^‐mediated reactions,^2^ mostly due to the instability of Au^III^ complexes and their propensity to undergo reduction to colloidal gold. In any case, most examples of homogeneous Au^III^ catalysis exploit the metal's Lewis acidity to activate heteroatoms or alkynes.

Certainly, the use of the redox pair Au^I^/Au^III^ has gained interest to trigger new carbon–carbon and carbon–heteroatom bond‐forming reactions.2a, 3 Over the last few years, examples of fast C−C cross‐coupling reactions under mild conditions have been reported through Au^I^/Au^III^ cycles.^4^ Similarly, some experimental studies have shed light into the C−X (X=halide) reductive elimination from gold complexes.^5^ Few examples of C(sp^2^)−E bond formation through reactions with O‐ and N‐nucleophiles have also been described.^6^

Further developments in this still young area of gold‐catalysed cross‐coupling reactions have been hampered by the poor mechanistic understanding of the individual steps along the proposed catalytic cycles. Elucidating these mechanisms has been a great challenge owing to the reactivity of high‐valent gold intermediates and the issues associated with the synthesis of such rather labile and rapidly evolving species. A strategy to overcome these limitations relies on the use of cyclometalated ligands that are able to stabilize Au^III^ ions by both the presence of at least one Au−C bond and the chelating effect of the resulting metallacycle. Nitrogen is one of the most commonly explored donor atoms for cyclometallation, giving rise to different types of bidentate or tridentate ligands, including C^N, C^N^N, C^N^C and N^C^N scaffolds.^7^

In this context, in 2018, Bochmann and co‐workers reported on the reaction of Au^III^ C^N^C pincer complexes (Figure 1) with thiols leading to the formation of aryl thioethers by cleavage of the pincer Au−C bonds by C−S reductive elimination.^8^ Of note, with the aim of introducing aryl moieties in proteins, in 2014, Wong and co‐workers tackled the possibility of derivatizing the pendant SH group of cysteine residues by C−S bond formation.^9^ In proof‐of‐concept experiments, exposure of different peptidic domains to an equimolar amount of a Au^III^ C^N complex (Figure 1) in aqueous environment at 37 °C for 24 h produced the corresponding aryl thioethers.^9^

**Figure 1:**
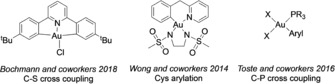
Structures of the Au^III^ organometallic complexes studied for various cross‐coupling reactions to achieve C−E (E=heteroatom) bond formation.

Following these promising results, we recently reported on the cysteine arylation by a series of C^N cyclometalated Au^III^ complexes upon reaction with a model of a zinc finger domain.^10^ By combining mass spectrometry and DFT calculations, initial mechanistic insights and structure–activity relationships were obtained, enabling the control of the reductive elimination in aqueous environment.^10^ As reported hereafter, we have now discovered that Au^III^ C^N complexes achieve C−P cross‐coupling reactions under mild conditions.

To the best of our knowledge, the only example of Au^III^‐mediated C−P reductive elimination has been reported by Toste and co‐workers in 2016.^11^ In this study, several phosphine‐supported Au^III^ organometallic complexes of general formula [(R_3_P)Au(aryl)Cl_2_] (Figure 1) were shown to undergo irreversible C_aryl_−P formation by reductive elimination to afford phosphonium salts when reacted with silver salts or Lewis bases. Previous work by the same group showed that C_aryl_−P cross‐coupling is feasible from aryldiazonium salts and *H*‐phosphonates by dual gold and photoredox catalysis.^12^ Moreover, aryl‐phosphoniums have occasionally been detected by NMR and/or MS as side‐products in gold‐catalysed transformations.^13^ Overall, these studies suggest that C−P coupling may be a decomposition pathway in catalytic transformations involving Au^III^−aryl species.

In general, reductive elimination plays a major role in transition‐metal mediated reactions (cross‐couplings in particular). It is the key product‐releasing step of many transformations. In contrast to oxidative addition, the feasibility of reductive elimination at gold was demonstrated experimentally early on. Nevertheless, the determinants and mechanisms of this reactivity are scarcely understood,^14^ which prevent its control.4a, 5e, 15

Following a different approach from the one of Toste and co‐workers,^11^ in this study, the C^N cyclometalated Au^III^ complex [Au(C^CO^N)Cl_2_] **1** 16 (C^CO^N=2‐benzoylpyridine) was found to directly mediate C_aryl_−P bond formation by reductive elimination. Upon reaction with 3 equivalents of 1,3,5‐triaza‐7‐phosphaadamantane (PTA) and 5 equivalents of KPF_6_ in acetone, complex **1** smoothly and cleanly gives the corresponding reductive elimination product (**5**) after 24 h at room temperature (Scheme 1).

**Scheme 1:**
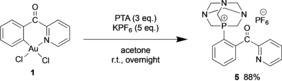
C−P coupling upon reaction of the C^N cyclometalated complex **1** with PTA (1,3,5‐triaza‐7‐phosphaadamantane).

Although the procedure reported in Scheme 1 is the optimized one, in a first synthetic attempt only 1 equivalent of PTA was reacted with [Au(C^CO^N)Cl_2_] **1** (C^CO^N=2‐benzoylpyridine) resulting in a very low yield of product **5** (ca. 10 %). However, when an excess of PTA was used (2 and 3 equivalents), the product's yields increased up to 49 and 88 %, respectively. Further addition of PTA did not improve the yield any further. Different counter anions, namely BF_4_^−^, NO_3_^−^ and B(C_6_F_5_)_4_^−^, were also investigated using the cyclometalated [Au(C^CO^N)Cl_2_] precursor. However, in all cases the yield of the reductive elimination product was markedly lower (10 % for BF_4_^−^, <1 % for NO_3_^−^ and <20 % for C_24_BF_20_^−^).

After purification by column chromatography, the coupling product **5** was characterised by ^31^P{^1^H}, ^1^H, ^13^C NMR, HR‐ESI‐MS (Figures S1–S4) and elemental analyses. Colourless needle‐shaped crystals were grown from a dichloromethane/*n*‐pentane mixture and an X‐ray diffraction study was performed (Figure 2, and supplementary material). The length of the formed C−P bond is typical for an aryl‐phosphonium at 1.796(2) Å. Note that the C=O bond in *ortho* position is quasi‐coplanar with a relatively short O…P distance (2.752(2) Å)^30^ suggesting the presence of some O→P interaction.^17^

**Figure 2:**
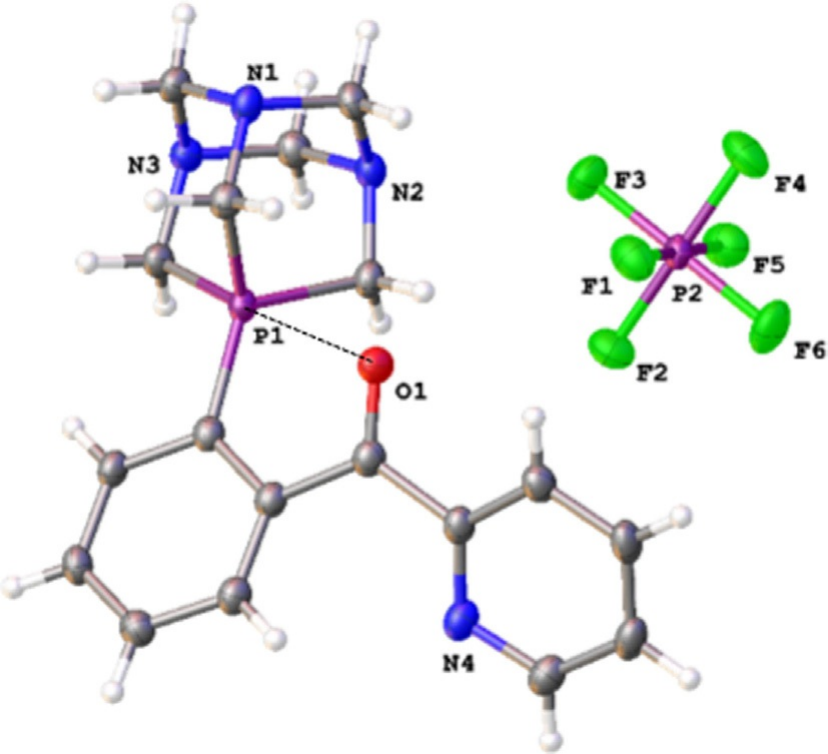
ORTEP plot of phosphonium **5**. Thermal ellipsoids are shown at 50 % probability.

To gain some mechanistic insight into the formation of the phosphonium **5**, the reaction of the Au^III^ precursor **1** with PTA (3 equiv) and KPF_6_ (5 equiv) in acetone was monitored by NMR spectroscopy. ^31^P{^1^H} NMR spectra were recorded at different time intervals: after 1 h, then every 3 h for the succeeding 18 h, with a final collection after 24 h. Afterwards, the reaction mixture was analysed using HR‐ESI‐MS to further characterise the products.

After 3 h, the ^31^P{^1^H} NMR spectrum of the reaction of **1** shows the appearance of a singlet peak at −55.7 ppm (Figure 3), which corresponds to the aryl‐phosphonium **5** and is shifted downfield compared to the signal of free PTA reagent (quintet at −102.3 ppm).^31^

**Figure 3:**
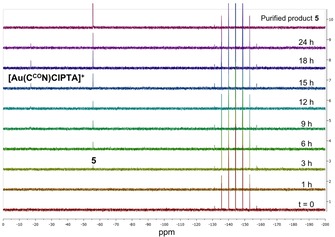
^31^P {^1^H} NMR spectra of the reaction mixture of **1** with 3 equivalents of PTA and 5 equivalents of KPF_6_ in [D_6_]acetone recorded over 24 h, compared to the spectrum of purified product **5**.

The intensity of the signal at −55.7 ppm increases over time as the reaction proceeds over 24 h. Moreover, after 6 h, another singlet peak appears at −17.1 ppm, which is assigned to the coordination product [Au(C^CO^N)Cl(PTA)]^+^, where the PTA ligand is bound to the gold atom *trans* to the N of the pyridyl group, as previously reported.^18^ In addition, the heptet at −144.3 ppm corresponding to the PF_6_^−^ counter‐anion is observed throughout the reaction. HR‐ESI‐MS confirms the formation of product **5** (339.1515 *m*/*z*), as well as the existence of the Au^III^ complex (571.0737 *m*/*z*) (Figure S5). This result supports the idea that [Au(C^CO^N)Cl(PTA)]^+^ is a likely intermediate in the C−P bond formation reaction.

To further explore the potential of the Au^III^‐mediated C−P cross‐coupling reaction, the synthesis of compound **5** was repeated in water instead of acetone as solvent. After 24 h an abundant precipitate was collected, dissolved in acetone and purified by column chromatography to afford **5** as a clean yellow solid in 70 % yield (slightly lower than the 88 % obtained from the reaction conducted in acetone). This result holds promise for the exploitation of Au^III^‐mediated reactions in biological aqueous environment.

To rationalize the results of the experimental investigations, DFT calculations were performed on the reaction of **1** with PTA. Based on the previously reported results on the C−S bond formation,^10^ and on the aforementioned experimental evidences, we postulated that PTA first coordinates to gold to form either [Au(C^N)Cl(PTA)]^+^ or [Au(C^N)(PTA)_2_]^2+^ and that C−P coupling then occurs through reductive elimination. In the case of [Au(C^N)Cl(PTA)]^+^, it is most likely that the C−P bond formation involves the complex with the aryl group and the phosphine in *cis* arrangement, due to the strong preference for reductive elimination to occur between groups located in *cis* position.

Due to the high electronic dissymmetry of the C^N chelate (C exerts a much stronger *trans* influence than N),^14^, ^19^ the PTA adduct with P in *trans* position to N (and thus, *cis* to the aryl group) is actually more favoured thermodynamically. This stereochemical preference has been supported experimentally in related PTA complexes,^18^ and by DFT calculations, according to which the difference in energy with the other diastereomer is very large (see Figure S6 and ref. [20]).

Based on these considerations, the hypothesized mechanisms for C_aryl_−P coupling are depicted in Scheme 2. The relative energy values and activation barriers calculated in acetone by DFT are reported in Figure 4 (Table S1). Cartesian coordinates of all the species considered are also reported in the Supporting Information. The calculations show that the first chloride/PTA substitution in compound **1** producing complex **R1** (Scheme 2) is a thermodynamically highly favoured process, with low activation energy (E_0_^≠^=13 kJ mol^−1^, Figures S4A, Table S1). Further reaction of **R1** with PTA can lead to two different routes: either a second Cl^−^/PTA substitution leading to **R2** or displacement of the pyridine moiety at gold by PTA (**Path 1**). The resulting Au^III^ complex **I1** would then undergo reductive elimination and C−P coupling (formation of **P_RE_**, Figure 4). Although the energy of the first transition state along **Path 1** (**TS1**) is very low (E_1_^≠^=0.4 kJ mol^−1^), the activation energy for the C−P reductive elimination (**TS1’**) is much higher (E_1’_^≠^=89 kJ mol^−1^). As far as **Path 2** is concerned, the energy of the transition state corresponding to the second Cl^−^/PTA substitution leading to product **R2** is also low (**TS00**, E_00_^≠^=8 kJ mol^−1^) and the resulting complex is slightly more stable than **R1** (Δ*E*=−4.3 kJ mol^−1^). Therefore, we hypothesize that the formation of **R2** is favoured with respect to **Path 1** in the presence of excess PTA (Figures 4, Table S1). Once **R2** is formed, even transiently, the reaction can proceed through the formation of intermediate **I2**, involving the displacement of the pyridine by a third PTA molecule. Of note, it was not possible to identify a transition state between **R2** and **I2**. The existence of reaction steps without energy barriers is well known, in particular those involving the reactivity of metal complexes.^32^ In fact, a good combination of leaving‐group lability, interaction energy between joining atoms, strain and steric hindrance of substituents in a transition state can lead to a vanishing activation energy barrier.^32^ Anyway, from **I2** the reaction continues towards C−P reductive elimination. The associated activation barrier (**TS2’**, E_2’_^≠^=55 kJ mol^−1^) is substantially smaller than that computed for **Path 1**. Note that the initially formed reductive elimination product **P_RE_** can easily form the conformer **P_RE_2** (Figure 4) after a simple rotation of the CO‐phenyl bond, which is about 18 kJ mol^−1^ more stable (Figure S26). Such conformer essentially corresponds to the obtained X‐ray crystal structure of **5**, showing a weak O→P coordination, with a calculated O−P distance of 2.66 Å in good agreement with that observed crystallographically.

**Scheme 2:**
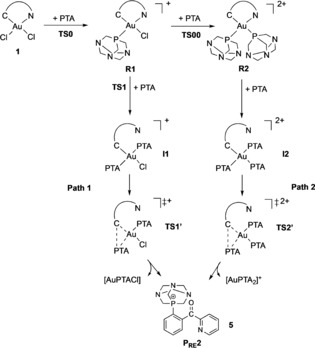
Proposed reaction mechanisms for the phosphine arylation reaction (C−P coupling) by the Au^III^ C^N complex **1**.

**Figure 4:**
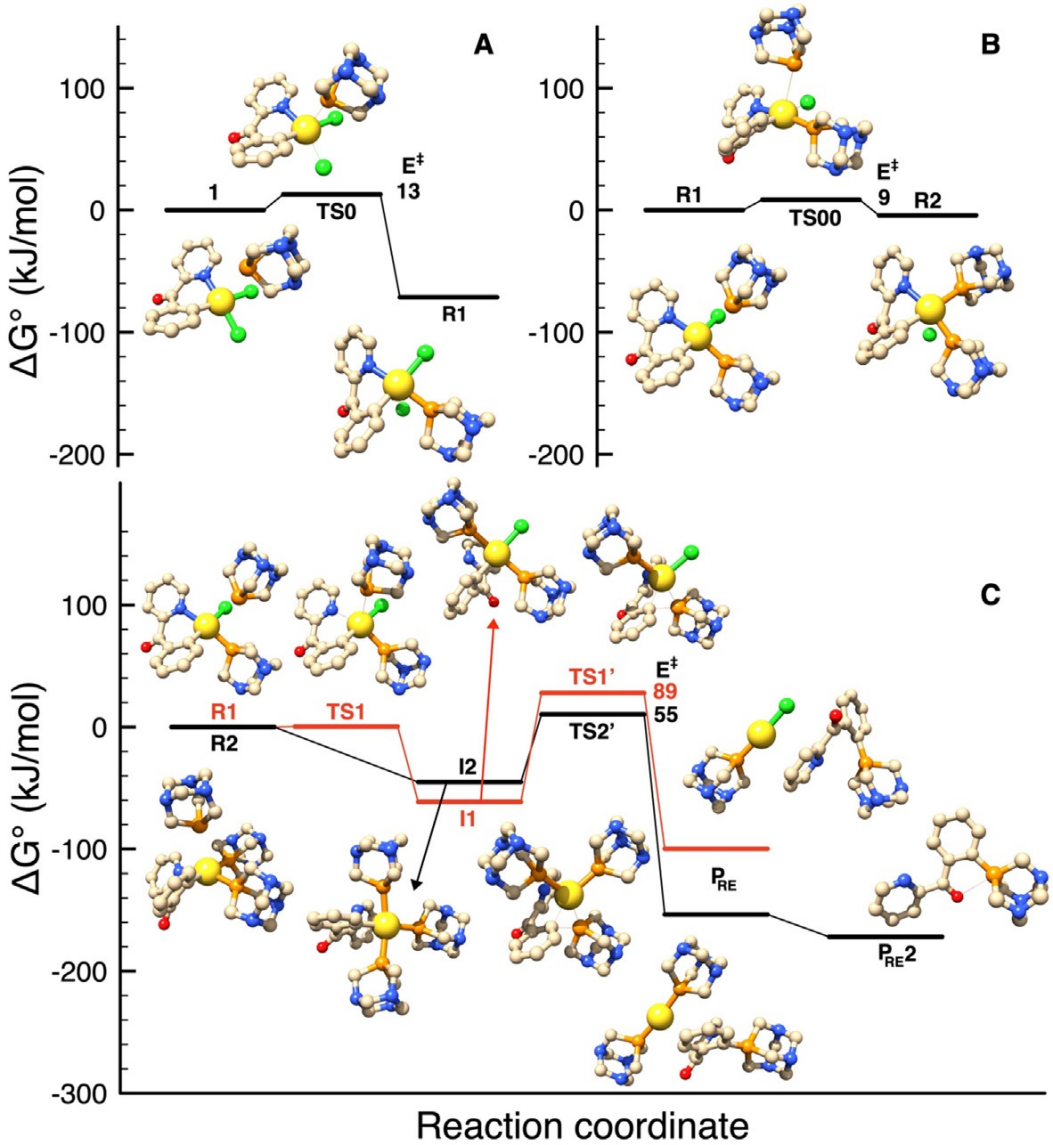
Species involved along the C−P cross‐coupling reaction pathway of compound [Au(C^CO^N)Cl_2_] (**1**) containing the C=O bridging group in the chelating ligand, undergoing Cl^−^/PTA substitution (to give **R1** (**A**) or **R2** (**B**)) and further reductive elimination (**C**). Structures and energies have been obtained by DFT calculations.

To assess the generality of the C−P coupling as observed from the C^N cyclometalated Au^III^ complex **1**, different ligand scaffolds were then investigated.

Complexes **2**–**4** featuring C^CH2^N (2‐benzylpyridine), C^NH^N (*N*‐phenylpyridin‐2‐amine) or C^‐^N (2‐phenyl‐pyridinate) ligands (Scheme S1) were synthesized according to literature procedures,^18^, ^21^ and reacted with PTA in the same conditions. In all cases, the corresponding phosphoniums were formed, as inferred by multi‐nuclear NMR, HR‐ESI‐MS (Figure S7–18) and elemental analyses, as well as X‐ray diffraction (for **6** and **7**, Figure S19). However, the efficiency of the coupling (as estimated from the isolated yields after column chromatography) depends markedly on the ligand backbone, decreasing from compound **5** to **8** (from 88 to 46, 42 and 16 % respectively). The latter observation is in line with the previously reported Cys arylation efficiency of the same cyclometalated Au^III^ complexes.^10^ In the case of compounds **1**–**3** not even a detailed computational analysis of the electronic and steric features of the three compounds could rationalize the observed differences in reactivity.^10^ Instead, in the case of compound **4**, DFT calculations showed that the higher steric demand nearby the C atom of the aryl group approaching the cysteinate residue, increases the activation barrier for C−S coupling.^10^ The same effect may account for the low yield of the herewith investigated C−P cross‐coupling.

Interestingly, ^31^P{^1^H} NMR monitoring of the reaction mixture between the [Au(C^CH2^N)Cl_2_] complex **2**, PTA and KPF_6_ shows at time 0 a unique peak at −56.6 ppm related to the phosphonium **6**, besides PF_6_^−^ (Figure S20). HR‐ESI‐MS further confirms the presence of **6** (325.1707 *m*/*z*) together with a second relevant specie at 511.1231 *m*/*z* (Figure S21). The latter could be identified as the secondary product of the reductive elimination where the reduced Au^I^ binds two molecules of PTA, forming the [Au(PTA)_2_]^+^ complex.

To gain further information on the intermediates involved in this reaction, the NMR study was repeated by lowering the temperature to 15 °C and recording spectra every 5 min over 100 min (Figure S22). Notably, at time 0, together with the peak related to **6**, a second more intense signal appears at −16.1 ppm, attributed to the [Au(C^CH2^N)Cl(PTA)]^+^ complex, as previously reported.^18^ Over time, whereas the intensity of the peak of **6** increases, the one of [Au(C^CH2^N)Cl(PTA)]^+^ decreases, suggesting the transformation of this species into the final product. To further confirm the involvement of the coordination species as a key reaction intermediate, [Au(C^CH2^N)Cl(PTA)]^+^ has been synthesised according to literature^18^ and subsequently reacted with 2 additional equivalents of PTA and 5 equivalents of KPF_6_. Following this procedure, **6** was formed after 24 h (74 % yield) without the need for further purification. Moreover, compound **1** was selected to extend the C−P cross‐coupling studies to other phosphines including triphenylphosphine (**9**), tri‐*n*‐butylphosphine (**10**) and tris(hydroxypropyl)phosphine (**11**).

In all cases, C−P bond formation was observed, demonstrating the generality of the reaction. The corresponding aryl‐phosphoniums were identified by ^31^P{^1^H} NMR^22^ and HR‐ESI‐MS (see Figures S23–S25) along with their respective Au^III^ precursors [Au(C^CO^N)Cl(phosphine)]^+^. In these cases, purification of the final products was more challenging due to the lower yield of the reductive elimination, as well as to the similar hydrophilic character of the cross‐coupling products and Au‐intermediates, making their chromatographic separation more challenging. Therefore, the C−P cross coupling will require further optimisation. However, it should be noted that in case of the reaction with the triphenylphosphine, we managed to obtain the X‐ray structure of the intermediate **I1** [Au(C^CO^N)Cl(triphenylphosphine)_2_]^+^ (Figure S27), which further supports our mechanistic hypothesis.

Organophosphorus compounds are one of the most important class of organic products, because of their broad applications in the field of materials science,^23^ medicinal chemistry,^24^ catalysis,^25^ and organic and inorganic synthesis.^26^ Classical synthetic strategies for the formation of C−P bond rely on the use of transition‐metal‐catalysed cross‐coupling processes,^27^ typically based on Pd, and in some cases Ni or Cu. However, new methods for synthesizing organophosphorus compounds need to be developed to improve the sustainability of these chemical processes.27b

Here, we have discovered that C^N cyclometalated Au^III^ complexes react with phosphines under mild conditions through C−P cross‐coupling. The reaction of complex **1** with PTA proceeds readily and in high yield, including in water. The transformation works with different tertiary phosphines. Of note, most metal‐mediated carbon–phosphorus bond‐forming reactions generate neutral products (i.e. phosphines, phosphites, and phosphinates).^28^ Reductive elimination of phosphoniums, as reported herewith, is far less precedented,^11^ and is typically observed with group 10 metals. ^[33]^ In addition, we have gained first mechanistic insights into the pathways of Au^III^‐mediated reductive elimination by a combination of NMR and DFT methods. Further studies are planned to elucidate the impact of the cyclometalated ligand and to determine the factors influencing C−P coupling at Au^III^, including solvent and counter‐anion effects, with the ultimate goal to exploit Au^III^ in catalytic transformations and bioconjugation reactions. Finally, the presented knowledge may be useful to increase efficiency of gold catalysis by preventing formation of by‐products, when C−P coupling is a side product. Moreover, this type of reactivity may open to new ways for transition‐metal mediated bio‐orthogonal reactions in living systems.^29^

## Conflict of interest

The authors declare no conflict of interest.

## Supporting information

As a service to our authors and readers, this journal provides supporting information supplied by the authors. Such materials are peer reviewed and may be re‐organized for online delivery, but are not copy‐edited or typeset. Technical support issues arising from supporting information (other than missing files) should be addressed to the authors.

Supplementary
