# Supplementary material for: Carbon–Phosphorus Coupling from C^N Cyclometalated AuIII Complexes
Source: Chemistry. 2020 Mar 6;26(19):4226–31. doi: 10.1002/chem.201905392 (PMC7187188; doi:10.1002/chem.201905392)
Supplement: Supplementary file 1 — Supplementary [file CHEM-26-4226-s001.pdf]

CHEMISTRY
A European Journal

Supporting Information

Carbon–Phosphorus Coupling from C^N Cyclometalated Au^{III} Complexes

Riccardo Bonsignore+,[a] Sophie R. Thomas+,[a] Wim T. Klooster,[b] Simon J. Coles,[b] Robert L. Jenkins,[a] Didier Bourissou,[c] Giampaolo Barone,*[d] and Angela Casini*[a, e]

chem_201905392_sm_miscellaneous_information.pdf

Supplementary Information Available

Experimental Section

General. Solvents and reagents (reagent grade) were all commercially available and used without further purification. ^{1}H, ^{13}C and $^{31}P\{^{1}H\}$ NMR spectra were recorded in Acetone-d_6 solution, with TMS as an internal reference, on Bruker Avance (400-500 MHz) NMR spectrometers. HR-ESI-MS spectra were recorded on Synapt G2-Si time-of-flight (TOF) mass spectrometer (Waters). Mass spectra were acquired and processed using MassLynx V4.1 (Waters). Compounds **1-4** have been synthesized following procedures already reported in literature.[1] The purity of the compounds was confirmed by elemental analysis.

General procedure for the synthesis of the compounds 5-8. The corresponding gold C^N organometallic complex, **1-4**, (1 eq., 40 mg, 0.09 mmol) was suspended in acetone (6 mL), followed by the addition of KPF_6 (5 eq., 84 mg, 0.44 mmol) and 1,3,5-triaza-7-phosphaadamantane (PTA, 3 eq., 43 mg, 0.27 mmol) (Scheme 1 and S1). The reaction mixture was stirred overnight at room temperature. On completion the solvent was removed under vacuum and the crude product was re-suspended in DCM before filtration. The filtrate was then purified by flash column chromatography (DCM:MeOH, gradient from 100:0 to 85:15, respectively) to obtain the clean product.

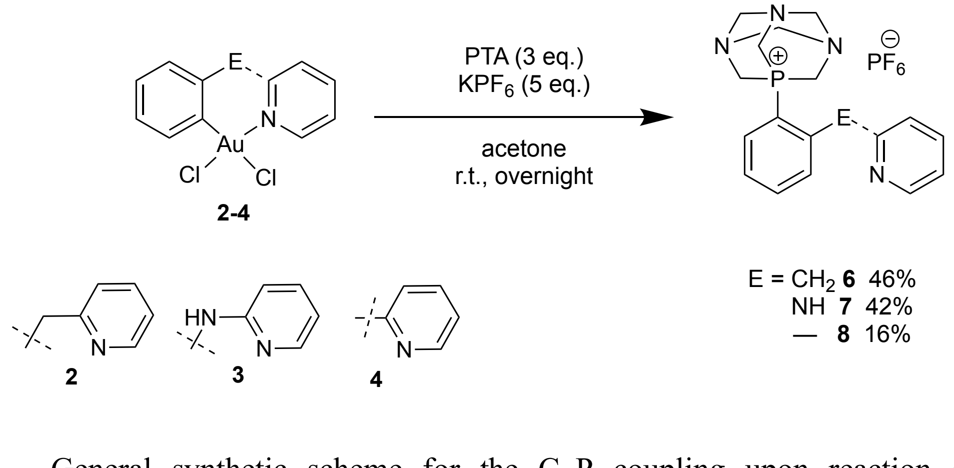

Scheme S1 – General synthetic scheme for the C–P coupling upon reaction of the C^N cyclometalated complex **2-4** with PTA (1,3,5-triaza-7-phosphaadamantane).

5: yellow solid, 38 mg, 88%. ^{1}H NMR (400 MHz, Acetone-*d_6*) δ 8.87 – 8.80 (m, 2H, ar), 8.31 – 8.26 (m, 1H, ar), 8.20 (td, *J* = 7.7, 1.7 Hz, 1H, ar), 8.08 – 8.00 (m, 3H, ar), 7.80 (ddd, *J* = 7.6, 4.7, 1.3 Hz, 1H, ar), 5.17 (d, *J* = 6.7 Hz, 6H, CH_2), 4.84 – 4.69 (m, 6H, CH_2). ^{13}C NMR (126 MHz, Acetone-*d_6*) δ 194.32 (d, *J* = 2.2 Hz, CO), 154.08 (s, ar), 149.85 (s, ar), 139.65 (d, *J* = 4.2 Hz, ar), 138.82 (s, ar), 137.29 (d, *J* = 8.2 Hz, ar), 136.22 (d, *J* = 9.4 Hz, ar), 135.08 (d, *J* = 3.3 Hz, ar), 134.92 (d, *J* = 12.8 Hz, ar), 128.78 (s, ar), 126.70 (s, ar), 122.28 (d, *J* = 56.7 Hz, ar), 72.65 (d, *J* = 10.0 Hz, CH_2), 51.78 (d, *J* = 32.3 Hz, CH_2). ^{31}P {^{1}H} NMR (162 MHz, Acetone-*d_6*) δ -55.67 (s, PTA), -144.26 (hept, *J* = 707.6 Hz, PF_6) ESI-MS (CH_3CN, pos. mode) for $[C_{18}H_{20}N_4OP]^+$: exp. 339.1375 (calc. 339.1382). Elemental analysis for $C_{18}H_{22}F_6N_4O_2P_2$ (**5** • H_2O): exp. C 43.30%, H 3.90%, N 11.30% (calc. C 43.04%, H 4.41%, N 11.15%).

6: white solid, 20 mg, 46%. ^{1}H NMR (400 MHz, Acetone-*d_6*) δ 8.76 – 8.73 (m, 1H, ar), 7.98 (ddd, *J* = 14.3, 7.9, 0.8 Hz, 1H, ar), 7.84 (td, *J* = 7.7, 1.8 Hz, 1H, ar), 7.75 – 7.66 (m, 1H, ar), 7.63 – 7.59 (m, 1H, ar), 7.59 – 7.53 (m, 2H, ar), 7.33 (ddd, *J* = 7.6, 4.9, 1.2 Hz, 1H, ar), 5.26 (d, *J* = 6.3 Hz, 6H, CH_2), 4.77 (s, 6H, CH_2), 4.29 (s, 2H, CH_2). ^{13}C NMR (126 MHz, Acetone-*d_6*) δ 159.23 (s, ar), 150.46 (s, ar), 146.12 (d, *J* = 8.0 Hz, ar), 138.79 (s, ar), 135.57 (d, *J* = 3.5 Hz, ar), 133.09 (d, *J* = 25.4 Hz, ar), 133.07 (d, *J* = 3.0 Hz, ar), 128.65 (d, *J* = 12.7 Hz, ar), 124.52 (s, ar), 123.27 (s, ar), 120.56 (d, *J* = 58.6 Hz, ar), 72.46 (d, *J* = 9.8 Hz, CH_2), 52.10 (d, *J* = 31.0 Hz, CH_2), 43.32 (d, *J* = 3.4 Hz, CH_2). ^{31}P {^{1}H} NMR (162 MHz, Acetone-*d_6*) δ -56.55 (s, PTA), -144.32 (hept, *J* = 707.4 Hz, PF_6). ESI-MS (CH_3CN, pos. mode) for $[C_{18}H_{22}N_4P]^+$: exp. 325.2334 (calc. 325.1582). Elemental analysis for $C_{18}H_{22}F_6N_4P_2$ (**6**): exp. C 45.98%, H 4.40%, N 11.75% (calc. C 45.97%, H 4.71%, N 11.91%).

7: brown solid, 18 mg, 42%. ^{1}H NMR (400 MHz, Acetone-*d_6*) δ 8.30 (s, 1H, NH), 8.24 (d, *J* = 3.9 Hz, 1H, ar), 7.89 (dd, *J* = 13.8, 7.8 Hz, 1H, ar), 7.81 (ddd, *J* = 9.2, 3.2, 1.6 Hz, 1H , ar), 7.77 – 7.70 (m, 1H, ar), 7.59 – 7.44 (m, 2H, ar), 6.97 (dd, *J* = 7.5, 4.4 Hz, 2H, ar), 4.97 (d, *J* = 6.8 Hz, 6H, CH_2),

4.71 – 4.56 (m, 6H, CH_2). ^{13}C NMR (126 MHz, Acetone-d_6) δ 158.61 (s, ar), 148.48 (s, ar), 147.30 (s, ar), 139.74 (s, ar), 136.82 (d, J = 3.1 Hz, ar), 133.40 (d, J = 9.9 Hz, ar), 128.76 (s, ar), 127.46 (d, J = 12.3 Hz, ar), 118.78 (s, ar), 117.81 (s, ar), 111.78 (s, ar), 72.42 (d, J = 9.9 Hz, CH_2), 50.96 (d, J = 32.9 Hz, CH_2). ^{31}P {^1H} NMR (162 MHz, Acetone-d_6) δ -58.38 (s, PTA), -144.24 (hept, J = 707.7 Hz, PF_6). ESI-MS (CH_3CN, pos. mode) for $[C_{17}H_{21}N_5P]^+$: exp. 326.1535 (calc. 326.1563). Elemental analysis for $C_{17}H_{21}F_6N_5P_2$ (**7**): exp. C 43.23%, H 4.45%, N 14.20% (calc. C 43.32%, H 4.49%, N 14.86%).

8: white solid, 7 mg, 16%. ^1H NMR (400 MHz, Acetone-d_6) δ 9.20 – 9.15 (m, 1H, ar), 8.23 (dd, J = 6.8, 5.1 Hz, 1H, ar), 8.17 (d, J = 8.1 Hz, 1H, ar), 8.07 (td, J = 8.0, 1.7 Hz, 1H, ar), 7.88 – 7.77 (m, 2H, ar), 7.71 – 7.65 (m, 1H, ar), 7.61 (ddd, J = 7.5, 4.9, 1.0 Hz, 1H, ar), 4.91 (d, J = 6.4 Hz, 6H, CH_2), 4.59 (q, J = 13.3 Hz, 6H, CH_2). ^{13}C NMR (126 MHz, Acetone-d_6) δ 153.93 (s, ar), 149.09 (s, ar), 144.45 (d, J = 3.8 Hz, ar), 140.45 (s, ar), 136.34 (d, J = 10.7 Hz, ar), 135.99 (d, J = 3.4 Hz, ar), 131.18 (d, J = 13.1 Hz, ar), 129.76 (d, J = 9.3 Hz, ar), 126.16 (s, ar), 122.70 (s, ar), 118.36 (d, J = 62.8 Hz, ar), 72.77 (d, J = 9.9 Hz, CH_2), 54.26 (d, J = 34.9 Hz, CH_2). ^{31}P {^1H} NMR (162 MHz, Acetone-d_6) δ -60.55 (s, PTA), -144.25 (hept, J = 707.5 Hz, PF_6). ESI-MS (CH_3CN, pos. mode) for $[C_{17}H_{20}N_4P]^+$: exp. 311.1500 (calc. 311.1425). Elemental analysis for $C_{23}H_{32}F_6N_4O_2P_2$ (**8** • 2 CH_3COCH_3): exp. C 48.66%, H 5.63%, N 9.79% (calc. C 48.70%, H 5.58%, N 9.79%).

X-ray diffraction analysis. Suitable crystals of complex **5** were obtained from a mixture of dichloromethane/n-pentane. Complexes **6** and **7** were allowed to form crystals by slow evaporation of acetone at room temperature. Furthermore, crystals of $[Au(C^{CO}N)Cl(triphenylphosphine)_2]^+$ were grown in a mixture of ethyl acetate/hexane. The crystals were analysed at the UK National Crystallography Service in Southampton. A suitable colourless needle-shaped crystal of **5** (0.200×0.080×0.040 mm^3), a colourless block-shaped crystal of **6** (0.360×0.280×0.120 mm^3), a colourless block-shaped crystal of **7** (0.320×0.240×0.150 mm^3) and suitable colourless block-shaped

crystal of [Au($C^{CO}N$)Cl(triphenylphosphine)$_2$]$^+$ (0.580×0.140×0.060 mm^3) were selected and mounted on a MITIGEN holder in perfluoroether oil on a Rigaku 007HF diffractometer equipped with Varimax confocal mirrors and an AFC11 goniometer and HyPix 6000HE detector. The crystals were kept at a steady T = 100.01(10) K during data collection. The structures were solved with the ShelXD[2] structure solution program using the Dual Space solution method and by using Olex2[3] as the graphical interface. Each model was refined with version 2014/7 of ShelXL [4] using Least Squares minimisation. CCDC codes 1947336 (**5**), 1941494 (**6**), 1941496 (**7**) and 1968702 ([Au($C^{CO}N$)Cl(triphenylphosphine)$_2$]$^+$).

$^{31}P\{^1H\}$ NMR studies. The reactions yielding **5-7** were monitored over 24 h by NMR spectroscopy on a Bruker Avance (400 MHz). The reaction mixtures were prepared by suspending 2 mg of **1**, **2** or **3** (1 eq.), 4 mg of KPF_6 (5 eq.) and 2.1 mg of PTA (3 eq.) in 0.7 ml of Acetone-d_6 in an NMR tube. Initially, spectra were acquired at room temperature at different time intervals (after the first hour, every 3 h for the subsequent 18 h and a final measurement at 24 h). The reaction yielding **6** was additionally studied at 15 °C collecting spectra every 5 min over 100 min.

Computational studies. DFT calculations were performed on the structure of compound **1**, as well as those of the species involved in its reaction pathways with the PTA ligand (see Scheme 2) following recently reported procedures,[5] using the M06-L DFT functional,[6] the Lanl2tz(f) basis set[7] for Au and the and the 6-31G(d,p) basis set[8] for P, Cl, O, N, C, and H atoms. Solvent effects were implicitly taken into account by full geometry optimization in the acetone solvent, reproduced by the polarizable continuum model (PCM).[9] The PF_6^- counter-anion has not been included in the calculations. Transition-state structures were found by the synchronous transit guided quasi-Newton method.[10] Vibration frequency calculations, within the harmonic approximation, were performed to check that each optimized geometry corresponded to a minimum or to a first-order saddle point (for transition-state structures) in the potential energy surface, and to evaluate their standard Gibbs free

energy values at 298.15 K. The Gibbs free energy values reported in Table S1were obtained by single point calculations on the optimized structures by using the Lanl2tz(f) for Au and expanding the all electron basis set to 6-311G(d,p)[11] for the other atoms. All calculations were performed by the Gaussian 09 program package.[12]

References

[1] aM. A. Cinellu, A. Zucca, S. Stoccoro, G. Minghetti, M. Manassero, M. Sansoni, *J. Chem. Soc., Dalton Trans.* **1996**, 4217-4225; bY. Zhu, B. R. Cameron, R. Mosi, V. Anastassov, J. Cox, L. Qin, Z. Santucci, M. Metz, R. T. Skerlj, S. P. Fricker, *J. Inorg Biochem.* **2011**, *105*, 754-762; cB. Bertrand, S. Spreckelmeyer, E. Bodio, F. Cocco, M. Picquet, P. Richard, P. Le Gendre, C. Orvig, M. A. Cinellu, A. Casini, *Dalton Trans.* **2015**, *44*, 11911-11918.

[2] G. M. Sheldrick, *Acta Crystallogr., Sect. C: Struct. Chem.* **2015**, *71*, 3-8.

[3] O. V. Dolomanov, L. J. Bourhis, R. J. Gildea, J. A. K. Howard, H. Puschmann, *J. Appl. Crystallog.* **2009**, *42*, 339–341.

[4] G. M. Sheldrick, *Acta Crystallogr., Sect. A* **2015**, *71*, 3–8.

[5] M. Wenzel, R. Bonsignore, S. Thomas, D. Bourissou, G. Barone, A. Casini, *Chem. –Eur. J.* **2019**, *25,* 7628-7634.

[6] Y. Zhao, D. G. Truhlar, *J. Chem. Phys.* **2006**, *125*, 194101.

[7] aL. E. Roy, P. J. Hay, R. L. Martin, *J. Chem. Theory Comput.* **2008**, *4*, 1029-1031; bK. L. Schuchardt, B. T. Didier, T. Elsethagen, L. Sun, V. Gurumoorthi, J. Chase, J. Li, T. L. Windus, *J. Chem. Inf. Model.* **2007**, *47*, 1045-1052.

[8] aM. M. Francl, W. J. Pietro, W. J. Hehre, J. S. Binkley, M. S. Gordon, D. J. DeFrees, J. A. Pople, *J. Chem. Phys.* **1982**, *77*, 3654-3665; bP. C. Hariharan, J. A. Pople, *Theor. Chim. Acta* **1973**, *28*, 213-222.

[9] J. Tomasi, B. Mennucci, R. Cammi, *Chem. Rev.* **2005**, *105*, 2999-3094.

[10] C. Peng, H. Bernhard Schlegel, *Isr. J. Chem.* **1993**, *33*, 449-454.

[11] aR. Krishnan, J. S. Binkley, R. Seeger, J. A. Pople, *J. Chem. Phys.* **1980**, *72*, 650-654; bA. D. McLean, G. S. Chandler, *J. Chem. Phys.* **1980**, *72*, 5639-5648.

[12] M. Frisch, G. Trucks, H. Schlegel, G. Scuseria, M. Robb, J. Cheeseman, G. Scalmani, V. Barone, B. Mennucci, G. Petersson, H. Nakatsuji, M. Caricato, X. Li, H. Hratchian, A. Izmaylov, J. Bloino, G. Zheng, J. Sonnenberg, M. Hada, M. Ehara, K. Toyota, R. Fukuda, J. Hasegawa, M. Ishida, T. Nakajima, Y. Honda, O. Kitao, H. Nakai, T. Vreven, J. Montgomery, J. Peralta, F. Ogliaro, M. Bearpark, J. Heyd, E. Brothers, K. Kudin, V. Staroverov, R. Kobayashi, J. Normand, K. Raghavachari, A. Rendell, J. Burant, S. Iyengar, J. Tomasi, M. Cossi, N. Rega, J. Millam, M. Klene, J. Knox, J. Cross, V. Bakken, C. Adamo, J. Jaramillo, R. Gomperts, R. Stratmann, O. Yazyev, A. Austin, R. Cammi, C. Pomelli, J. Ochterski, R. Martin, K. Morokuma, V. Zakrzewski, G. Voth, P. Salvador, J. Dannenberg, S. Dapprich, A. Daniels, Farkas, J. Foresman, J. Ortiz, J. Cioslowski, D. Fox, *Gaussian 09, Revision A.1, Gaussian, Inc., Wallingford CT* **2009**.

Figures

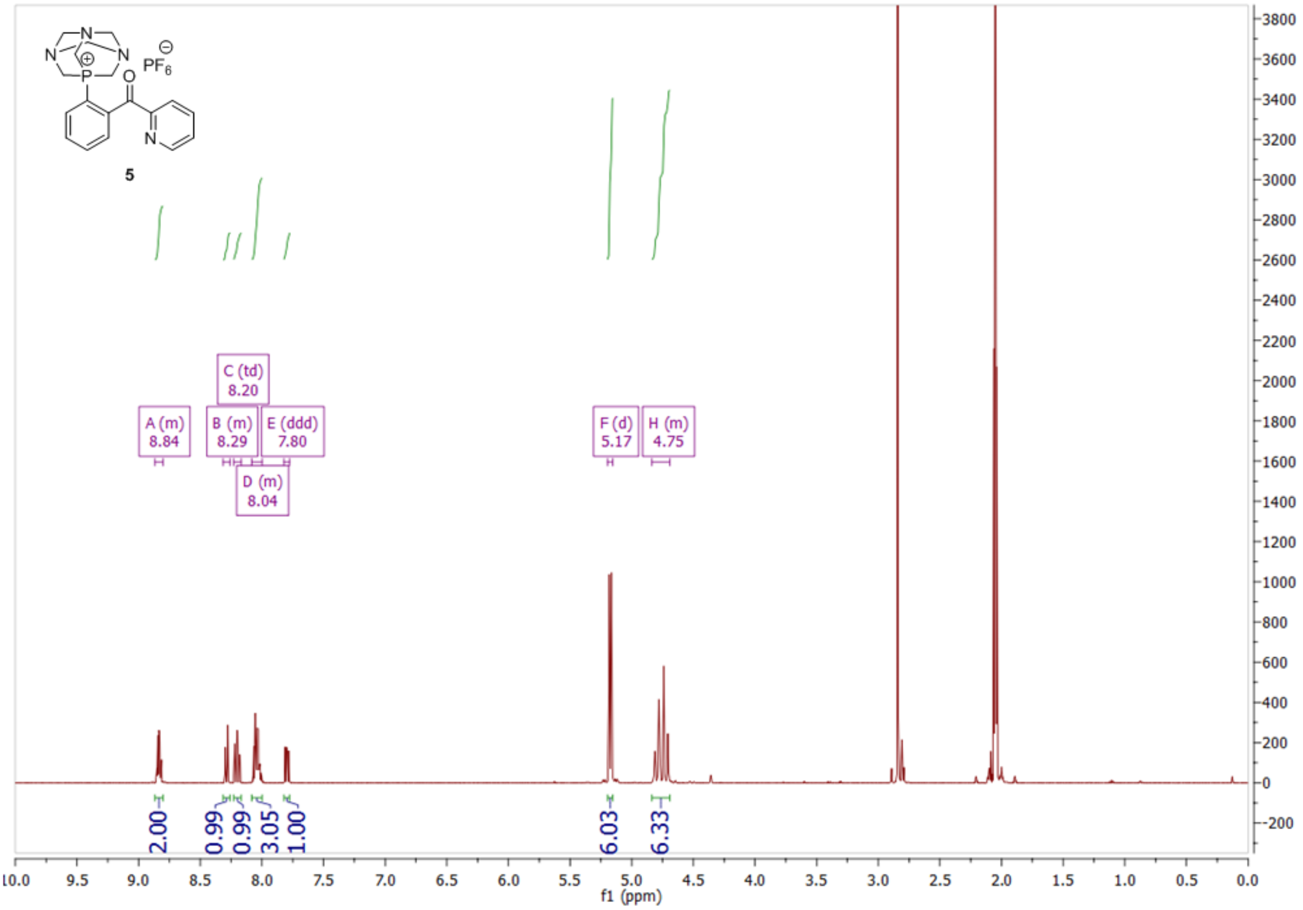

Figure S1. ^{1}H NMR spectrum of **5** in Acetone-d_6.

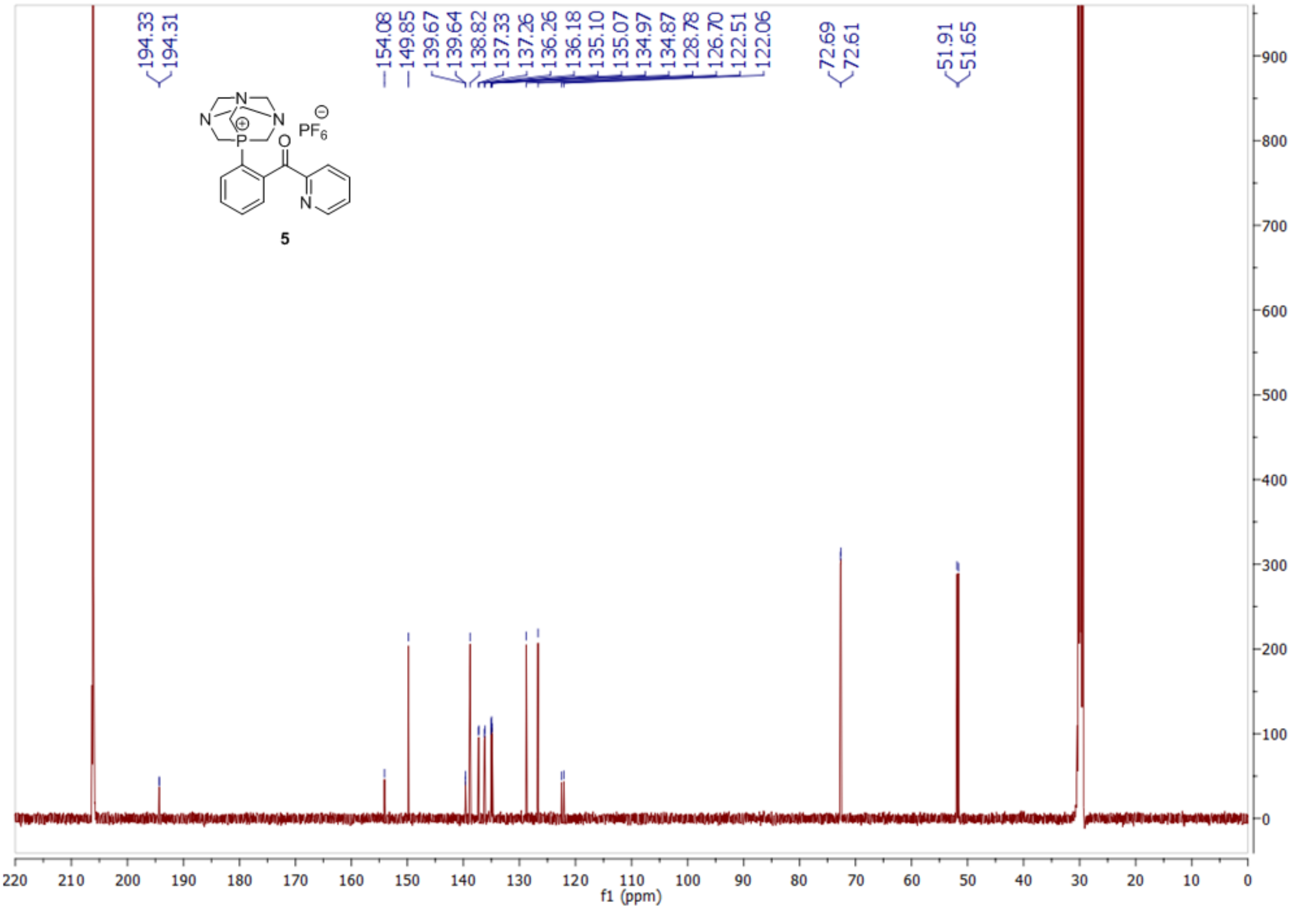

Figure S2. ^{13}C NMR spectrum of **5** in Acetone-d_6.

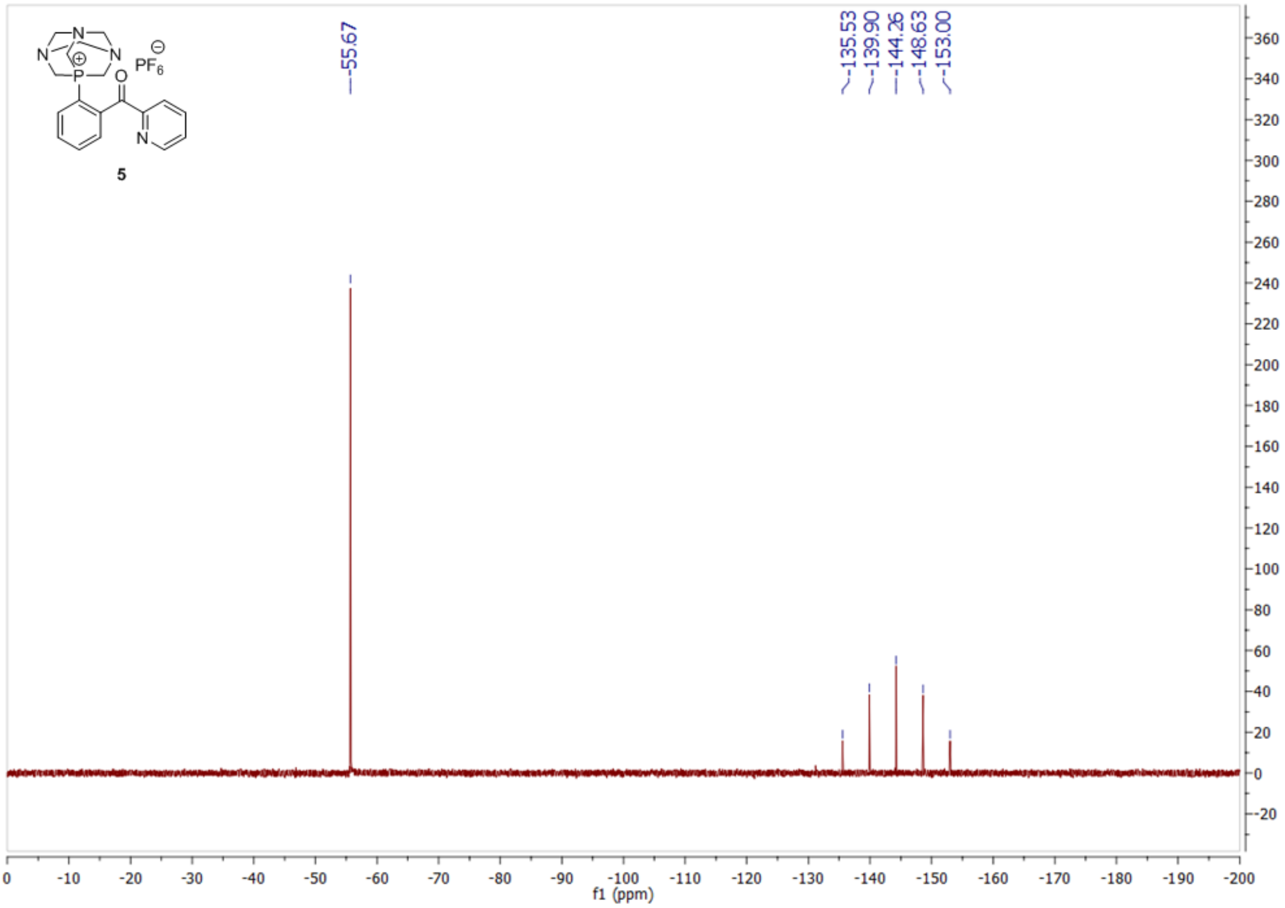

Figure S3. $^{31}P\{^{1}H\}$ NMR spectrum of **5** in Acetone-d_6.

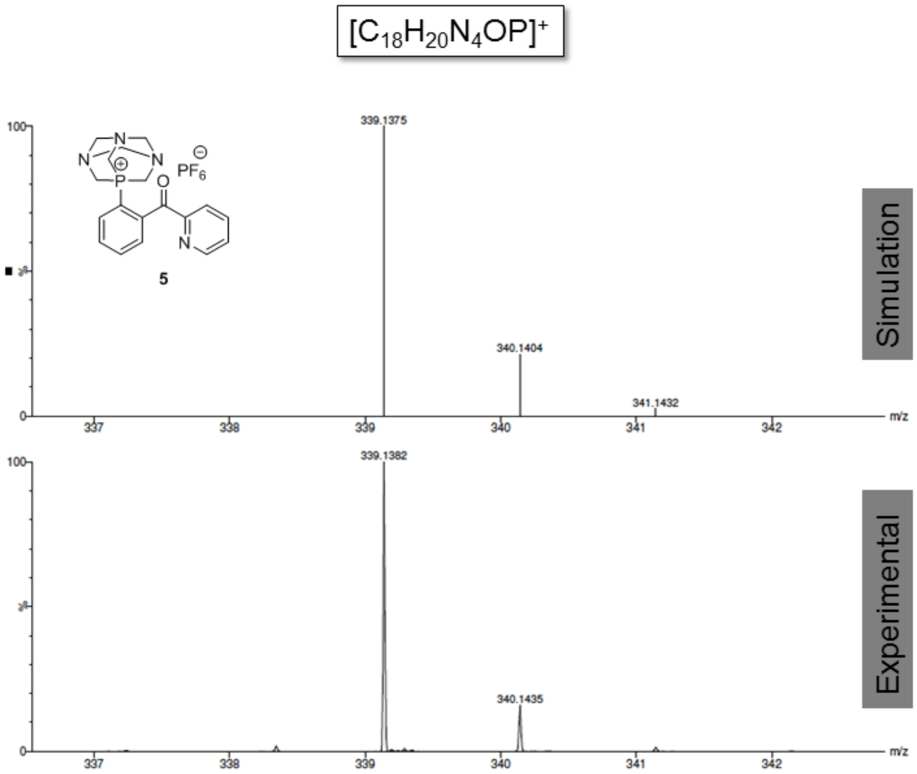

Figure S4. HR-ESI-MS simulated (top) and experimental (below) spectra of **5**.

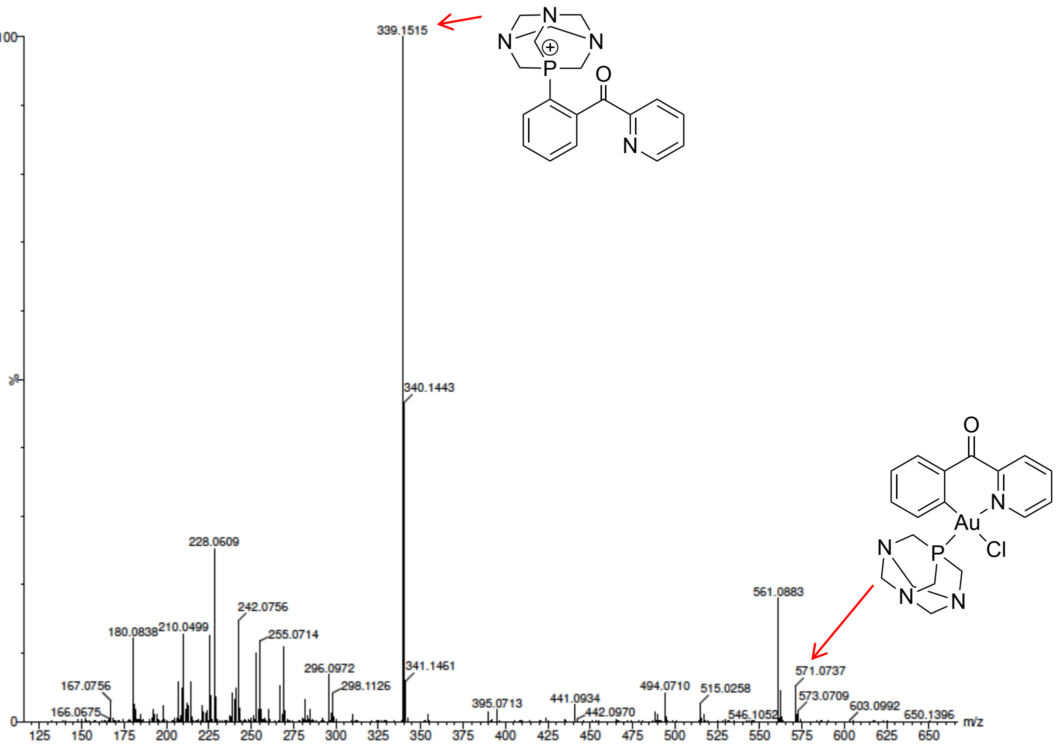

Figure S5. HR-ESI-MS spectra of the reaction mixture of **1** with 3 eq. of PTA and 5 eq. of KPF_6 after 24 h in Acetone-d_6, forming **5** and the corresponding coordination species.

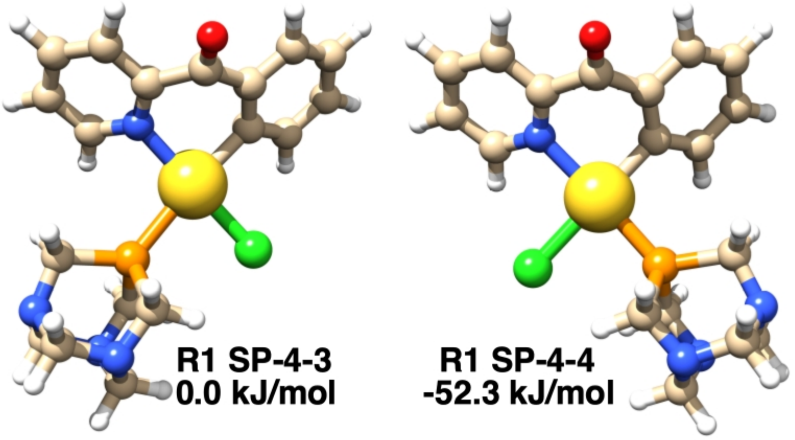

Figure S6. Structure and relative stability in acetone of the possible stereoisomers of the $[Au(C^{CO}N)Cl(PTA)]^+$ complexes (**R1** in Scheme 2), obtained by DFT calculations.

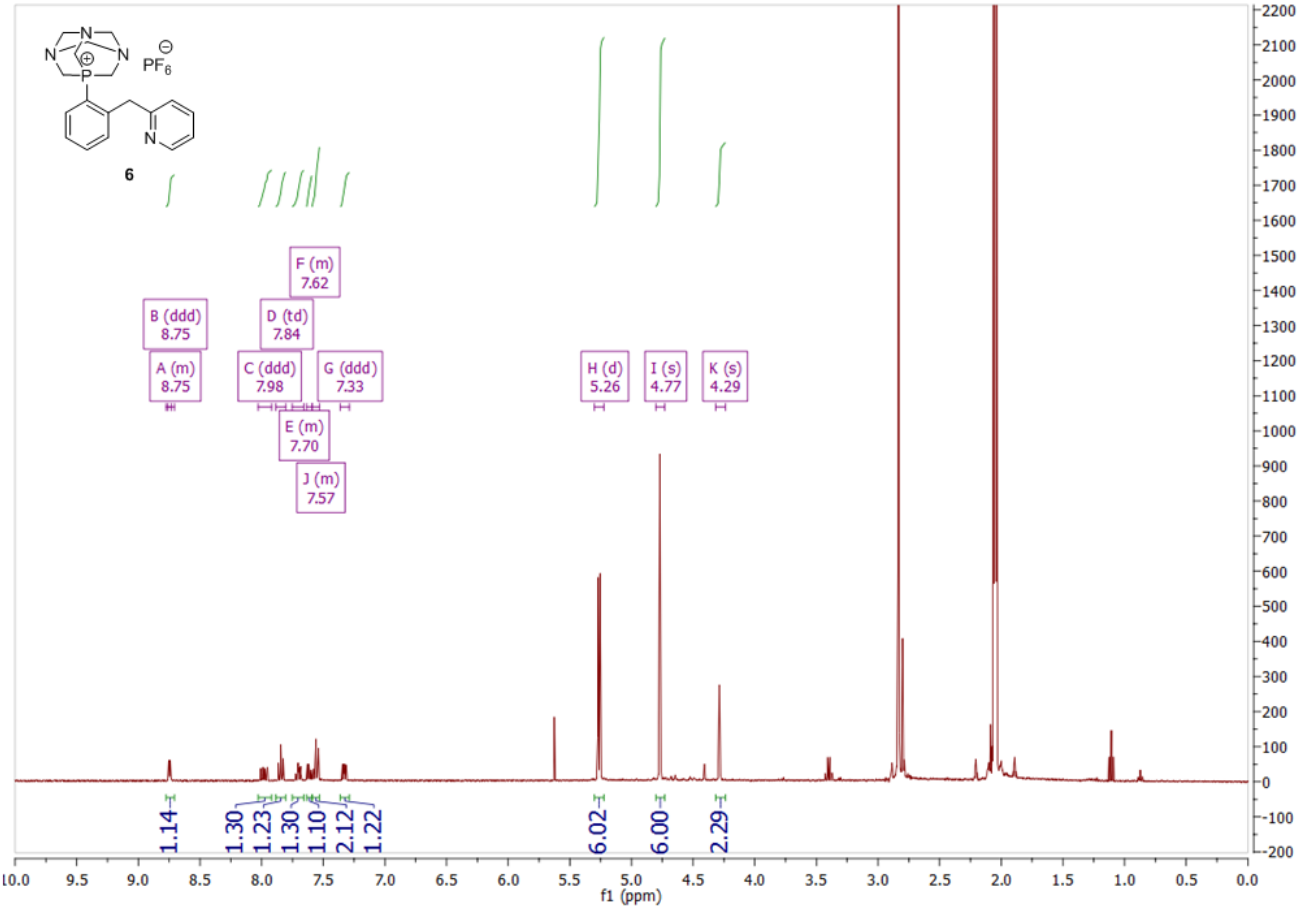

Figure S7. ^{1}H NMR spectrum of **6** in Acetone-d_6.

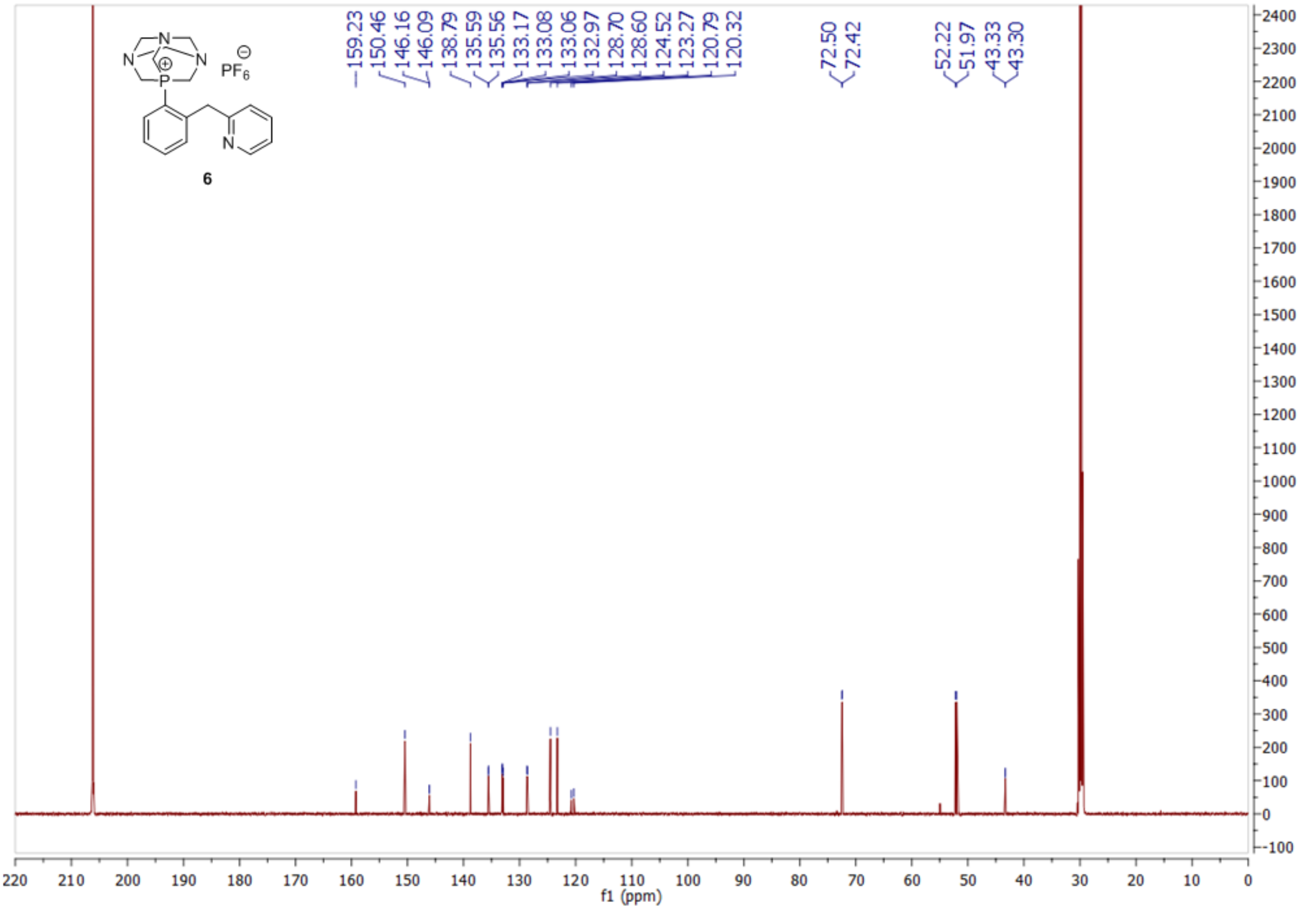

Figure S8. ^{13}C NMR spectrum of **6** in Acetone-d_6.

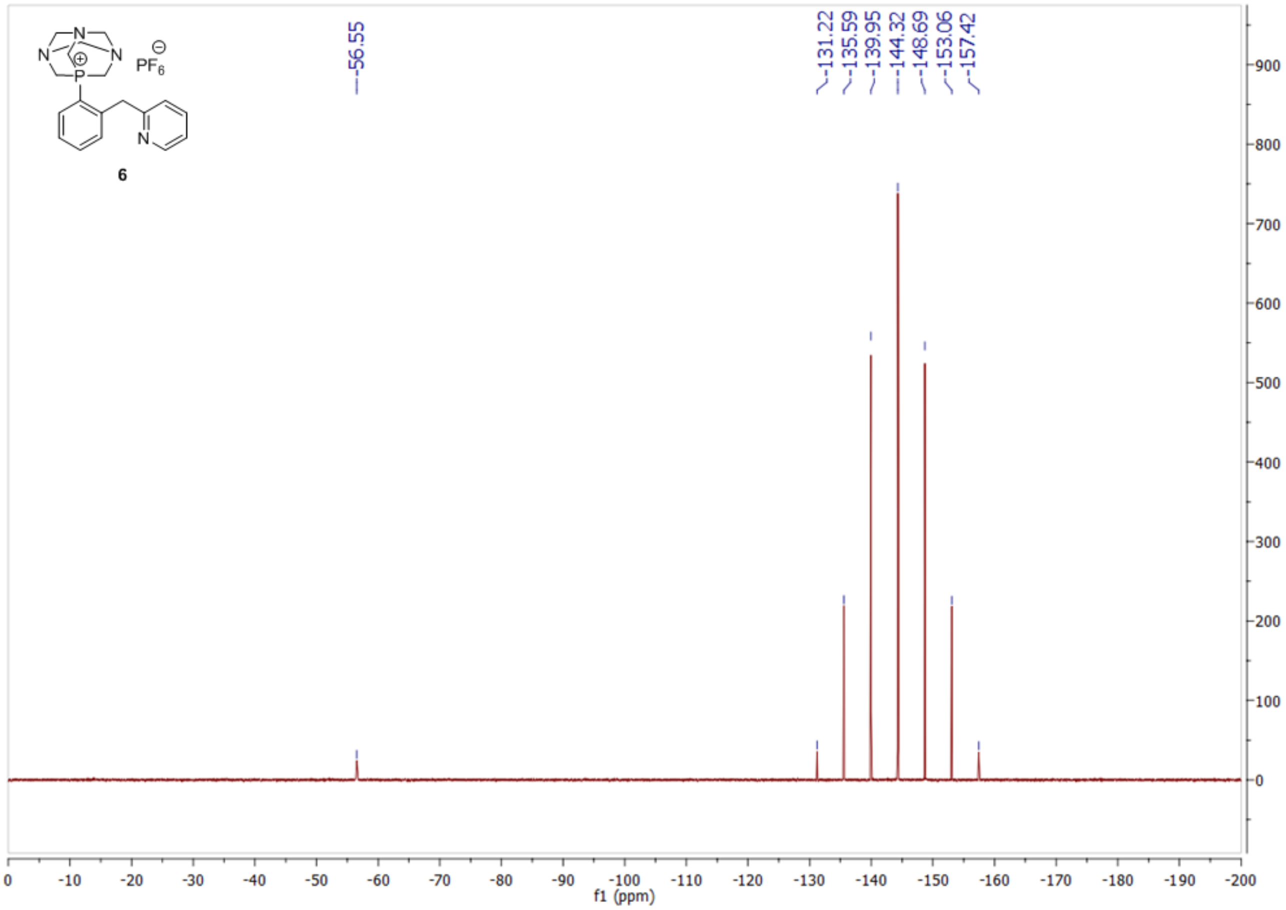

Figure S9. $^{31}P\{^{1}H\}$ NMR spectrum of **6** in Acetone-*d6*.

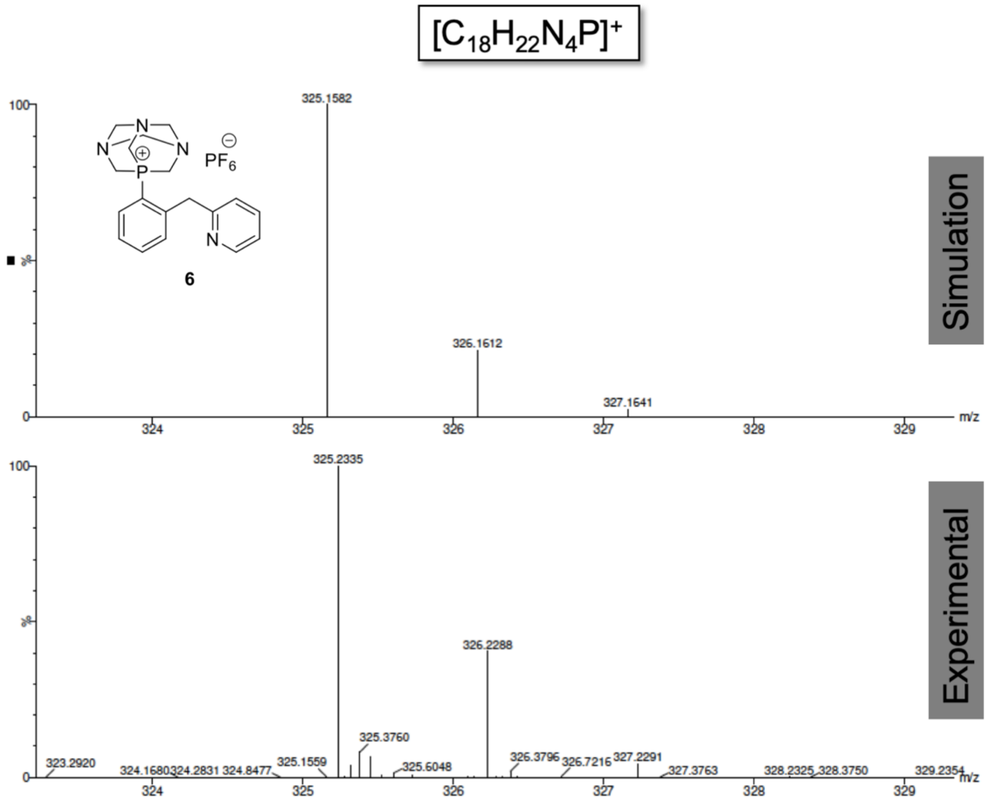

Figure S10. HR-ESI-MS simulated (top) and experimental (below) spectra of **6**.

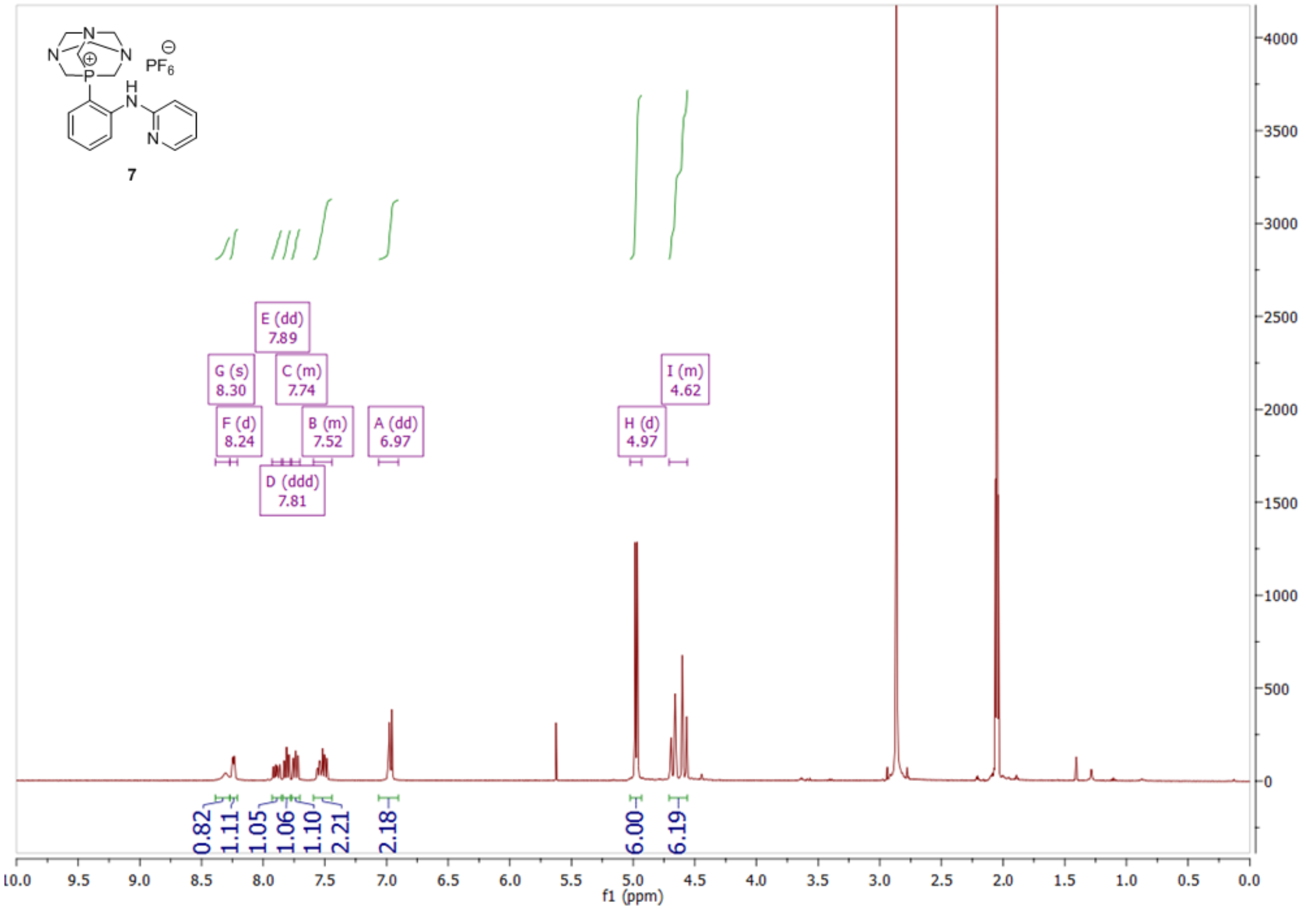

Figure S11. ^{1}H NMR spectrum of **7** in Acetone-d_6.

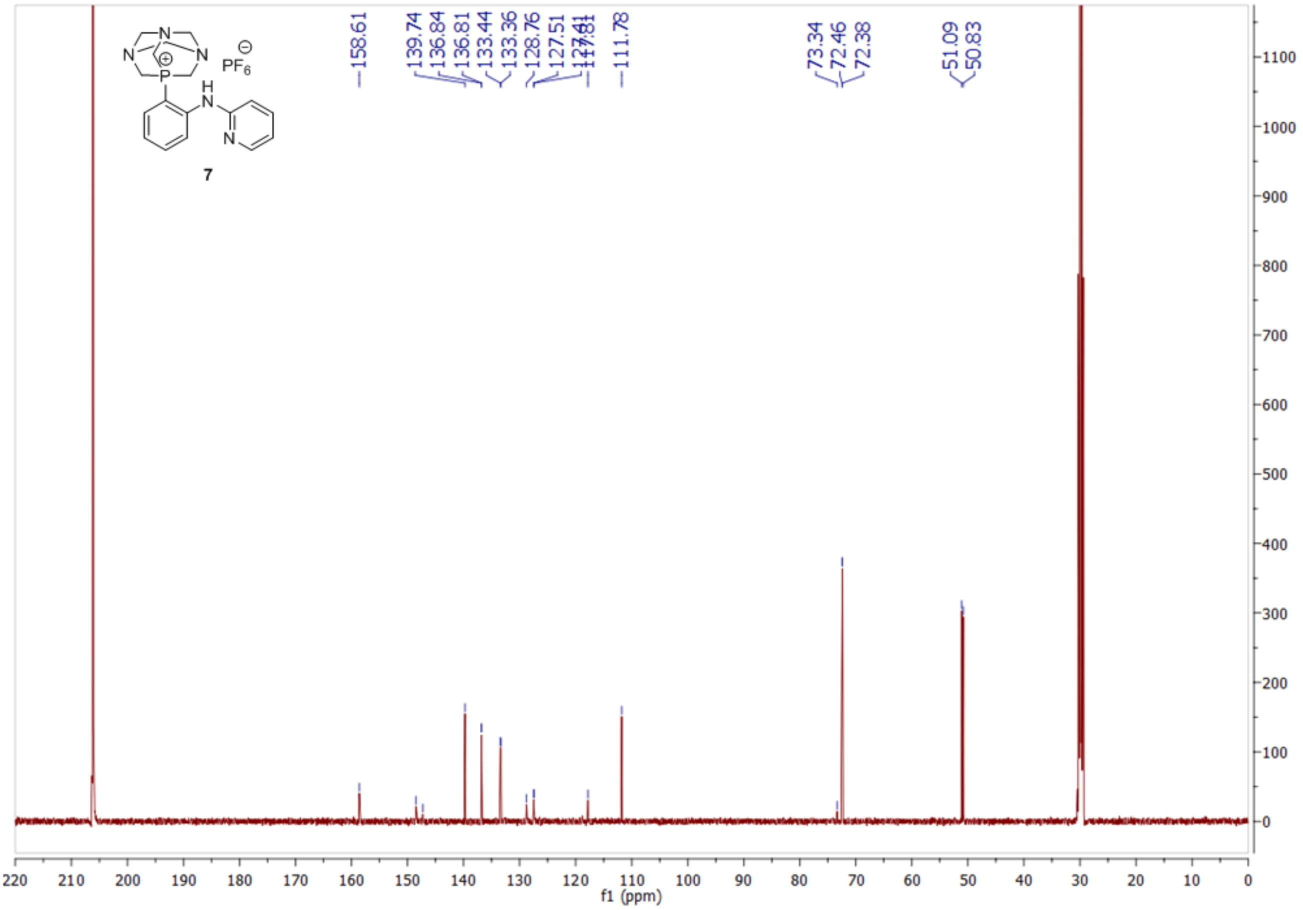

Figure S12. ^{13}C NMR spectrum of **7** in Acetone-d_6.

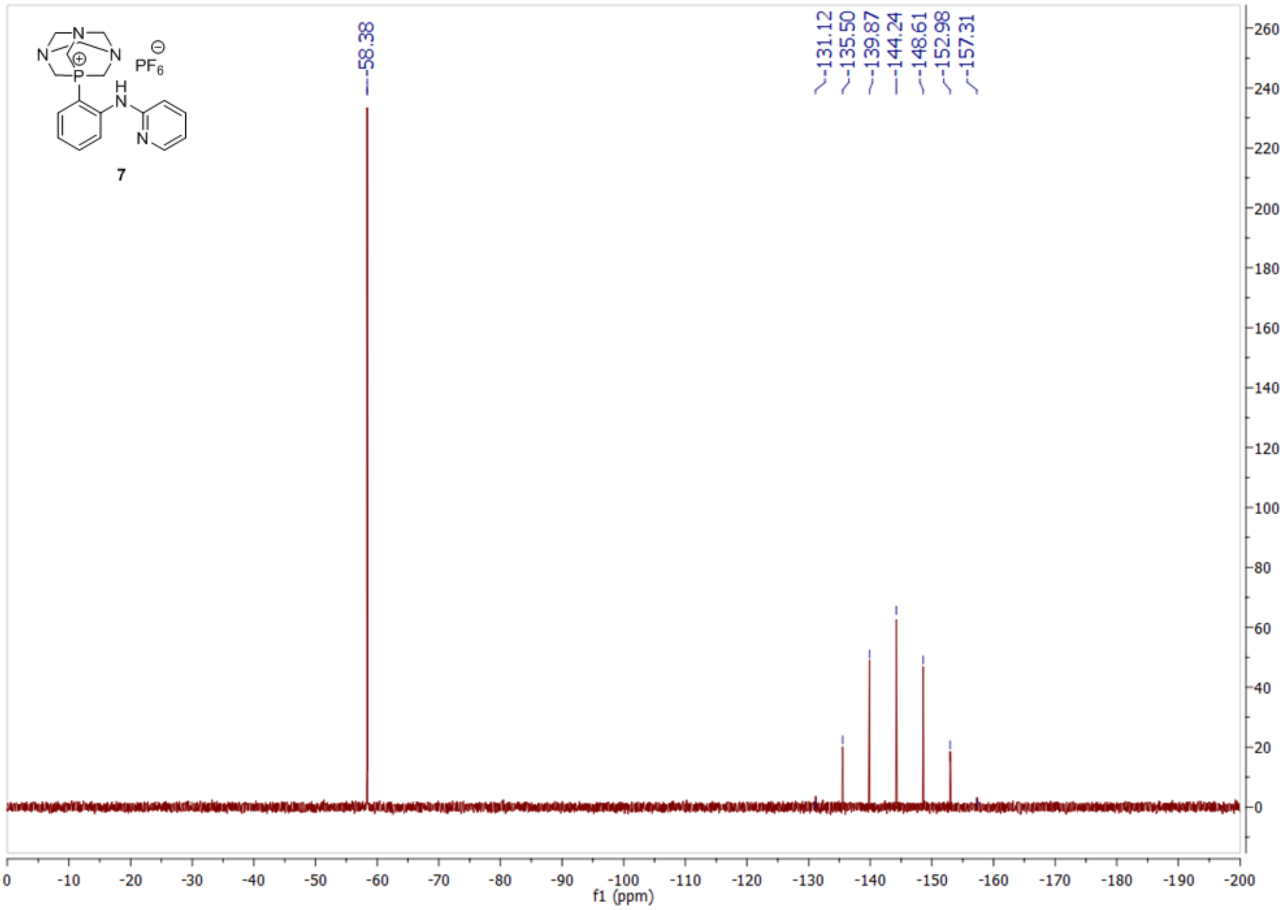

Figure S13. $^{31}P\{^{1}H\}$ NMR spectrum of **7** in Acetone-d_6.

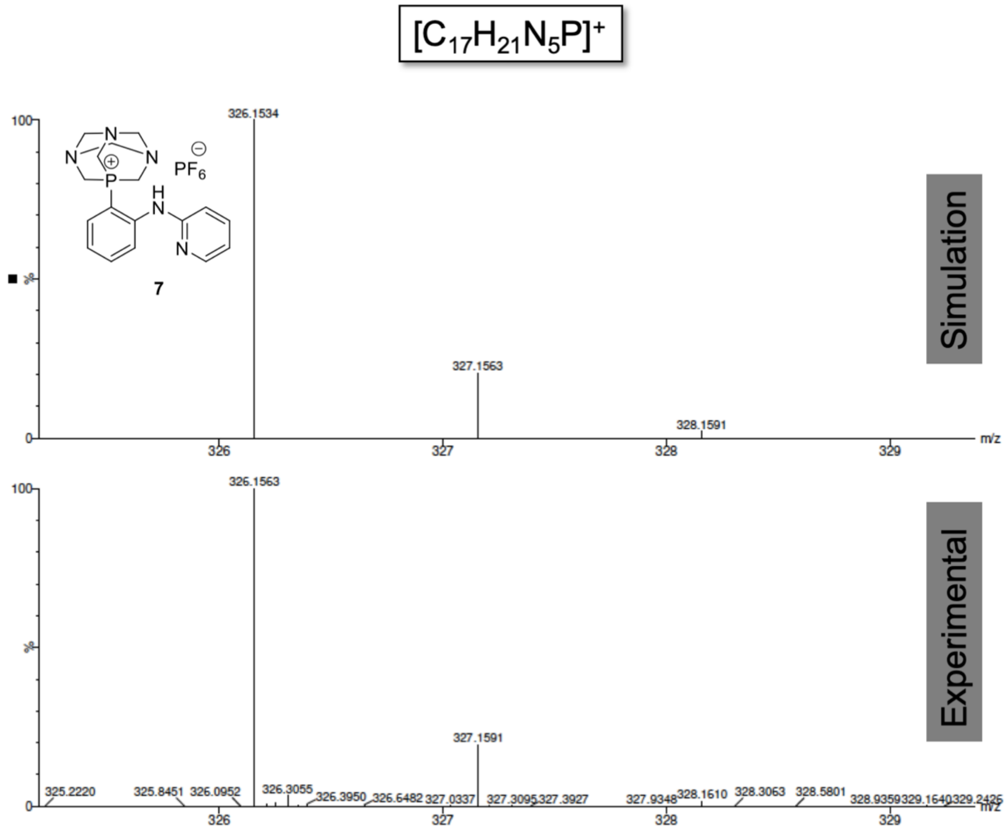

Figure S14. HR-ESI-MS simulated (top) and experimental (below) spectra of **7**.

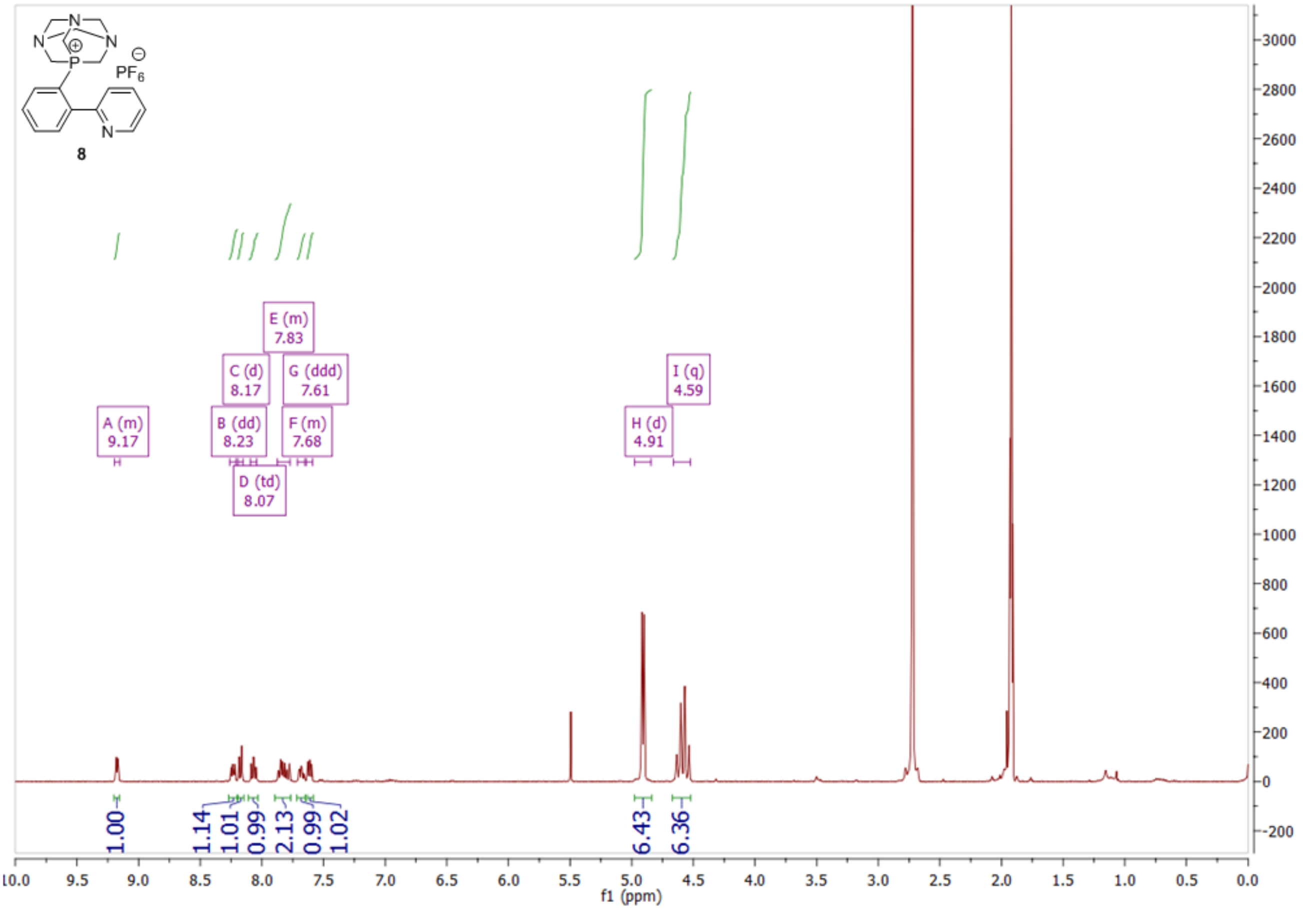

Figure S15. ^{1}H NMR spectrum of **8** in Acetone-d_6.

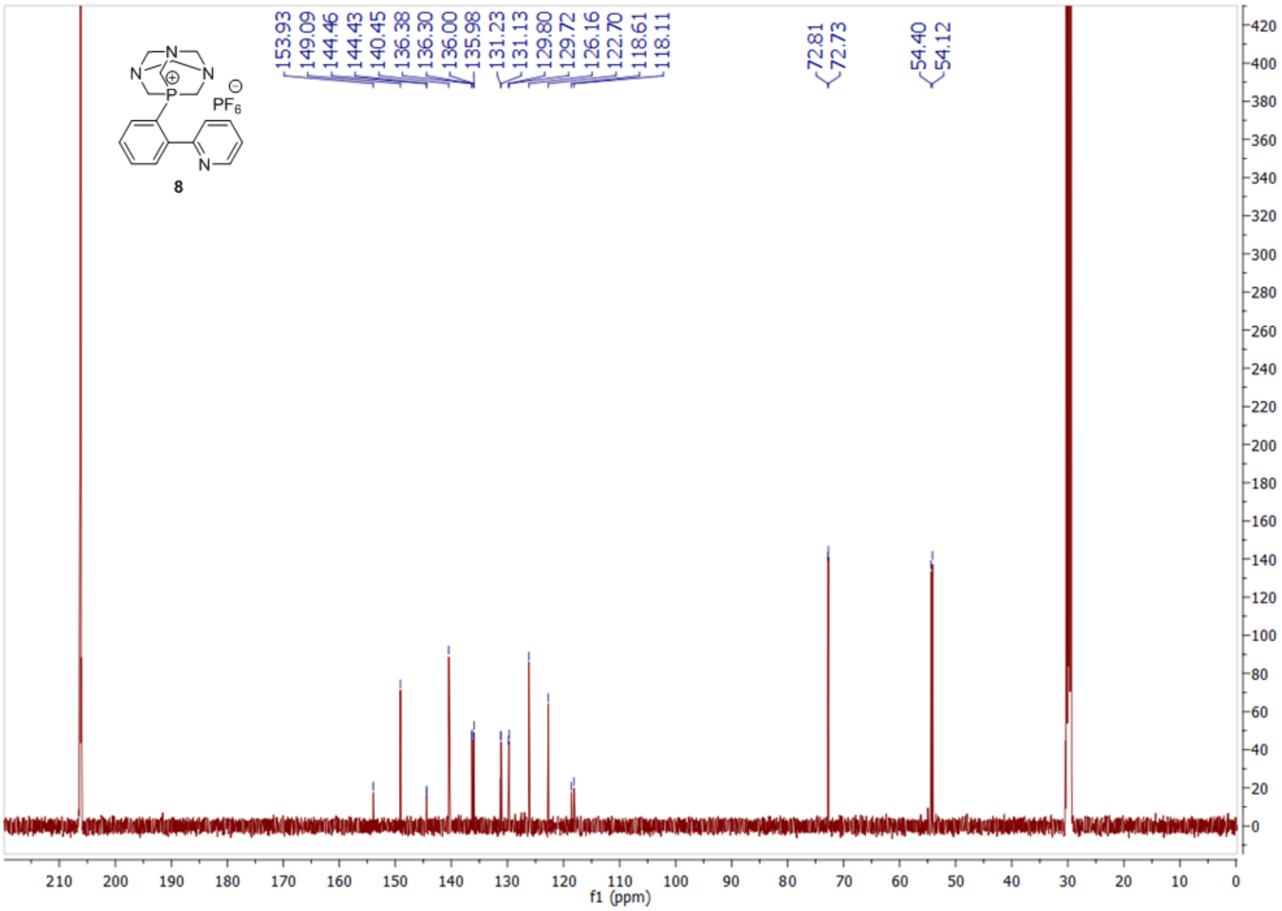

Figure S16. ^{13}C NMR spectrum of **8** in Acetone-d_6.

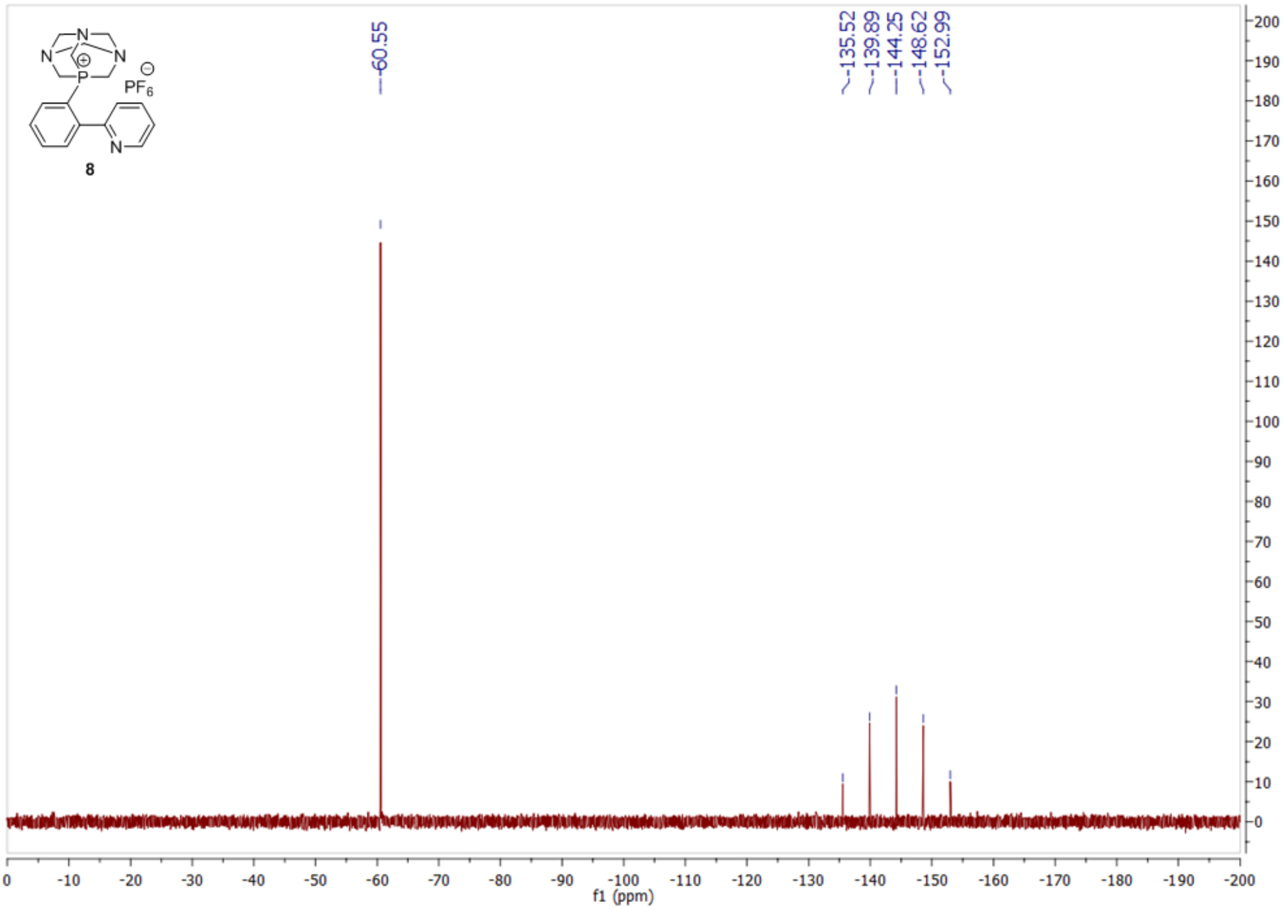

Figure S17. $^{31}P\{^{1}H\}$ NMR spectrum of **8** in Acetone-*d*$_6$.

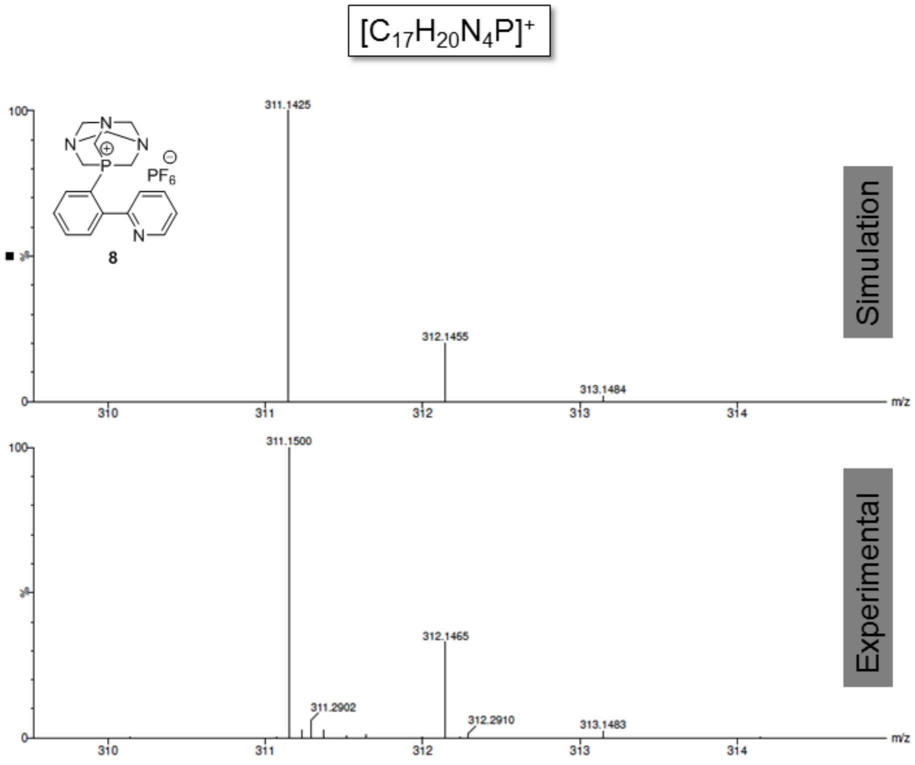

Figure S18. HR-ESI-MS simulated (top) and experimental (below) spectra of **8**.

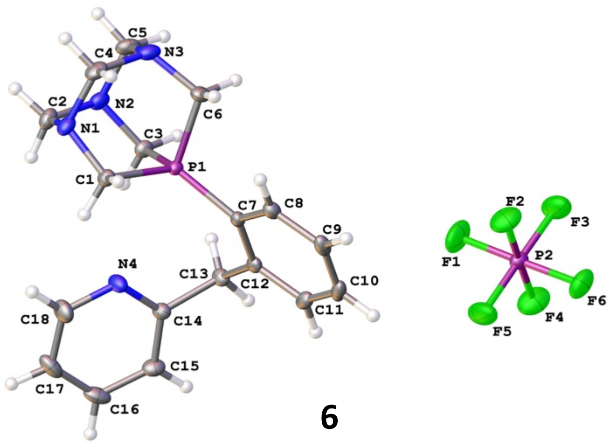

6

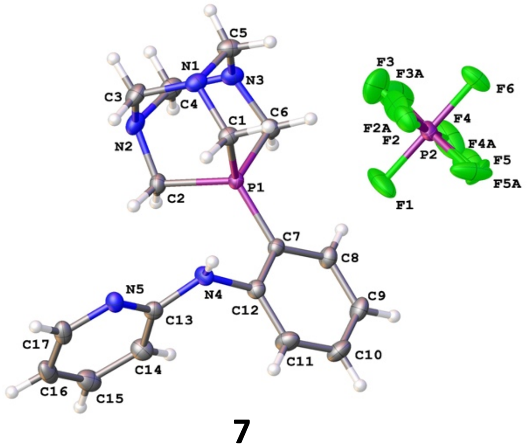

7

Figure S19. ORTEP plot of phosphonium compounds **6-7**. Thermal ellipsoids are shown at 50% probability.

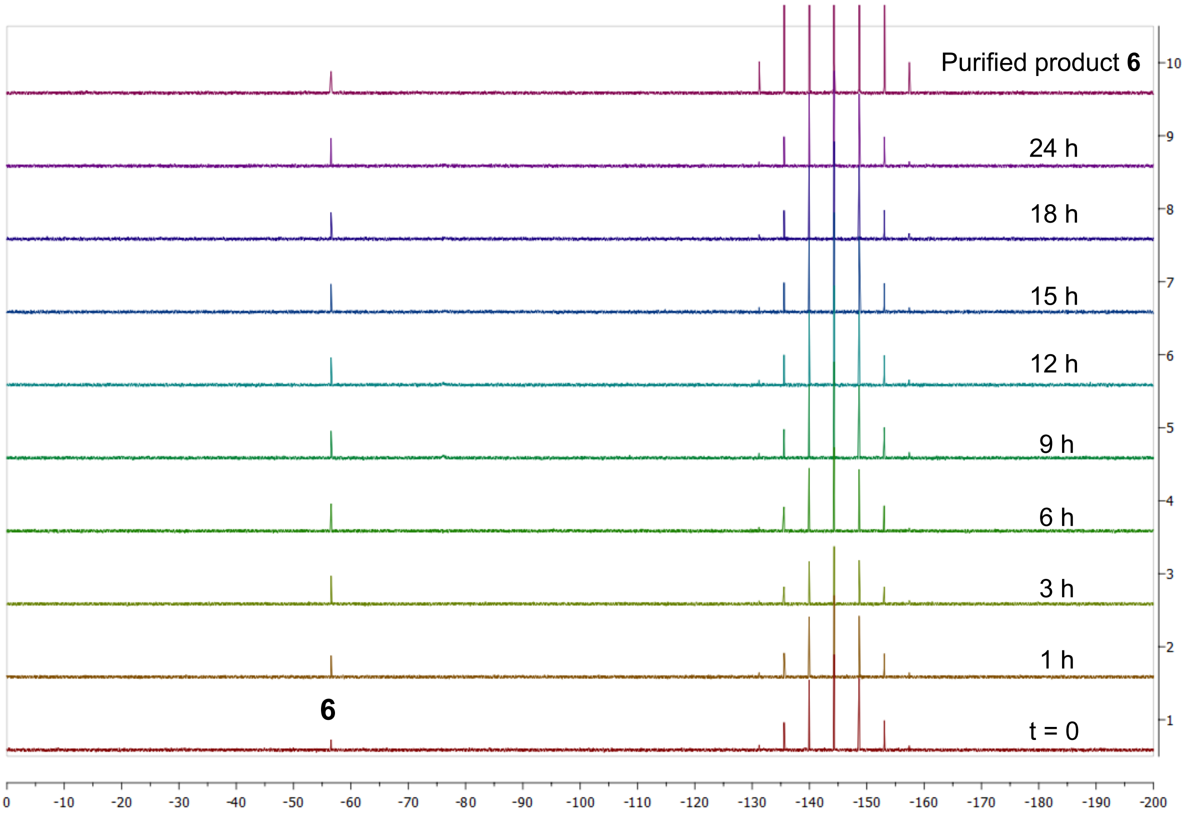

Figure S20. $^{31}P\ \{^{1}H\}$ NMR spectra of the reaction mixture of **2** with 3 eq. of PTA and 5 eq. of KPF_6 in Acetone-d_6 recorded over 24 h, compared to the spectrum of purified product **6**.

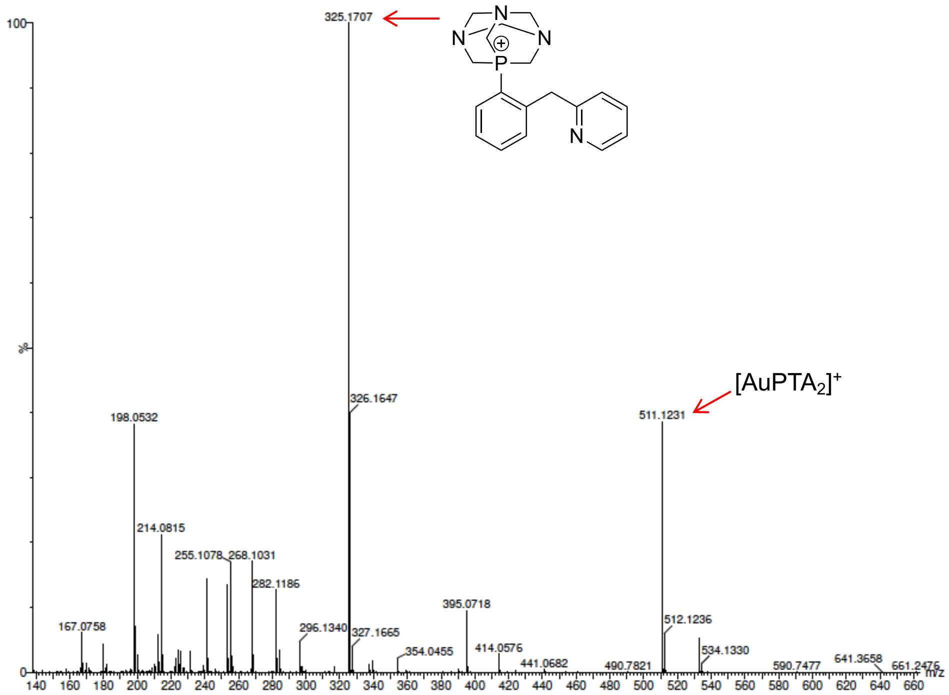

Figure S21. HR-ESI-MS spectra of the reaction mixture of **2** with 3 eq. of PTA and 5 eq. of KPF_6 after 24 h in Acetone-d_6, forming **6** and Au(I) coordination species resulting from the reductive elimination.

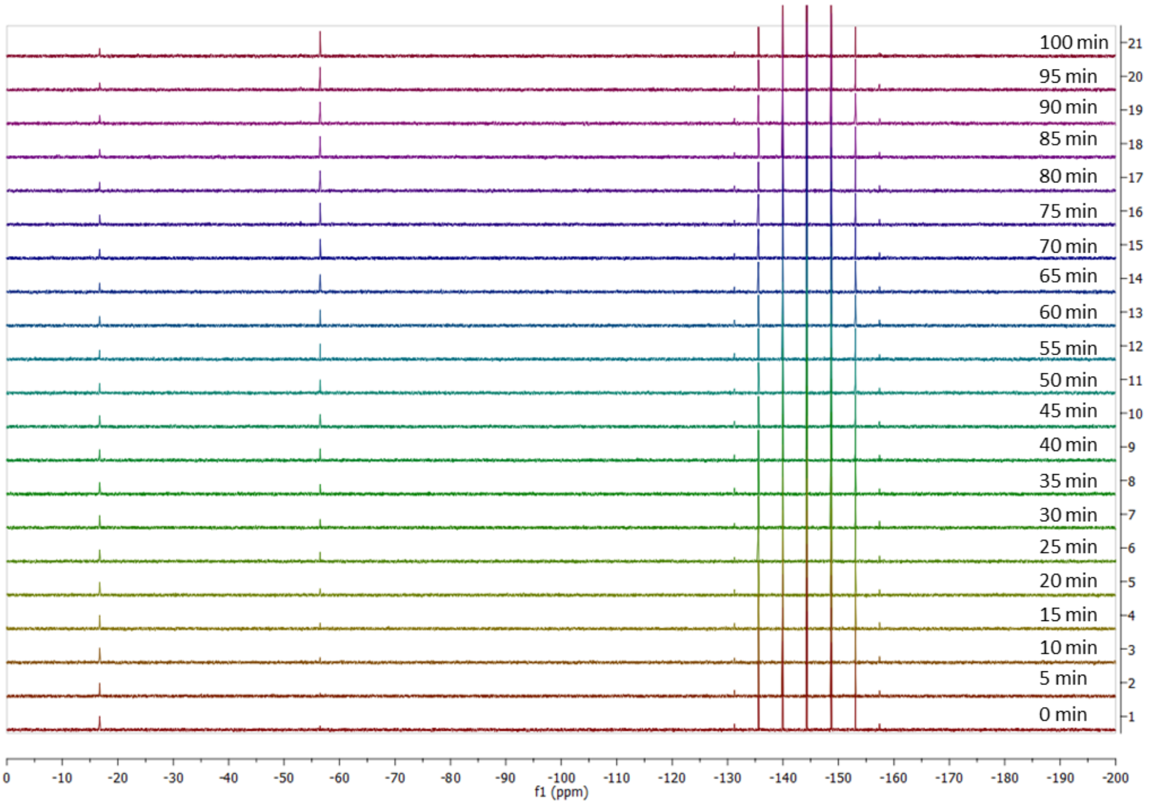

Figure S22. ^{31}P {^{1}H} NMR spectra of the reaction mixture of **2** with 3 eq. of PTA and 5 eq. of KPF_6 in Acetone-d_6 recorded over 100 min and 15°C.

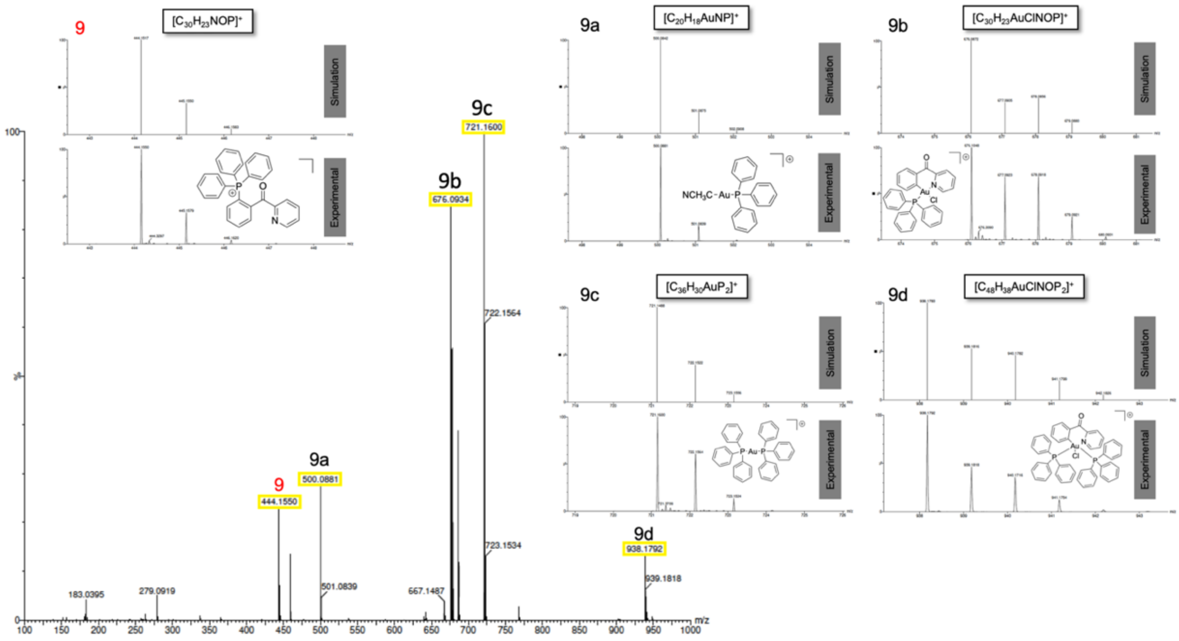

Figure S23. HR-ESI-MS spectrum of **9** and other species (**9a-d**) formed during the reaction of **1** with triphenylphosphine. Inserts show simulated (top) and experimental (bottom) spectra of **9** and **9a-d**.

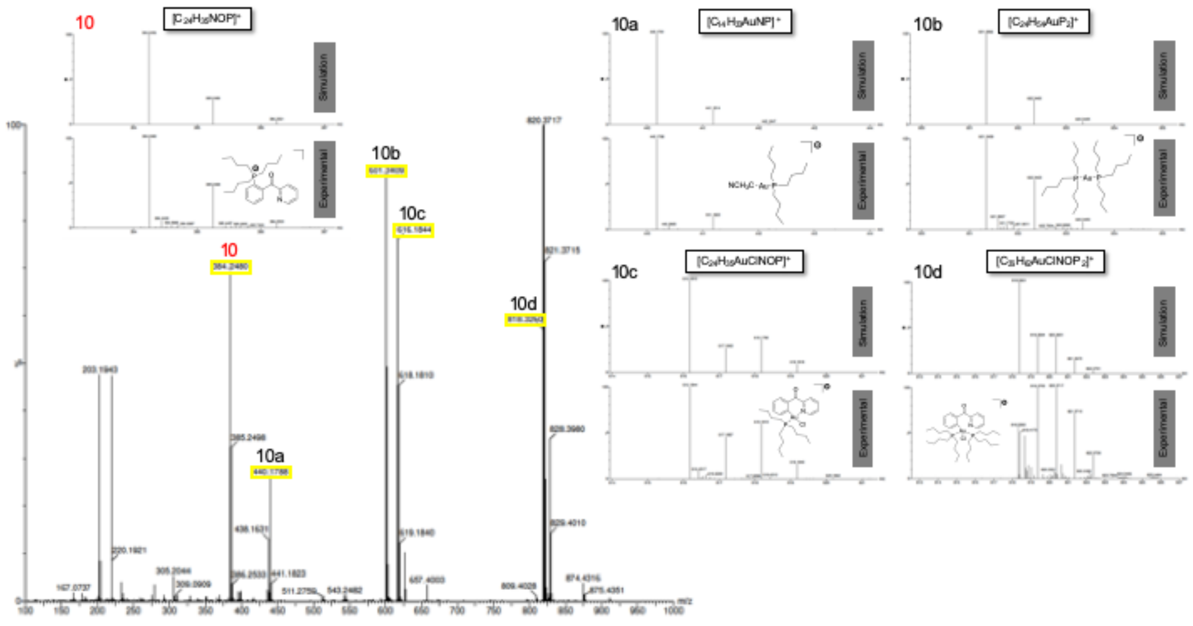

Figure S24. HR-ESI-MS spectrum of **10** and other species (**10a-d**) formed during the reaction of **1** with tri-n-butylphosphine. Inserts show simulated (top) and experimental (bottom) spectra of **10** and **10a-d**.

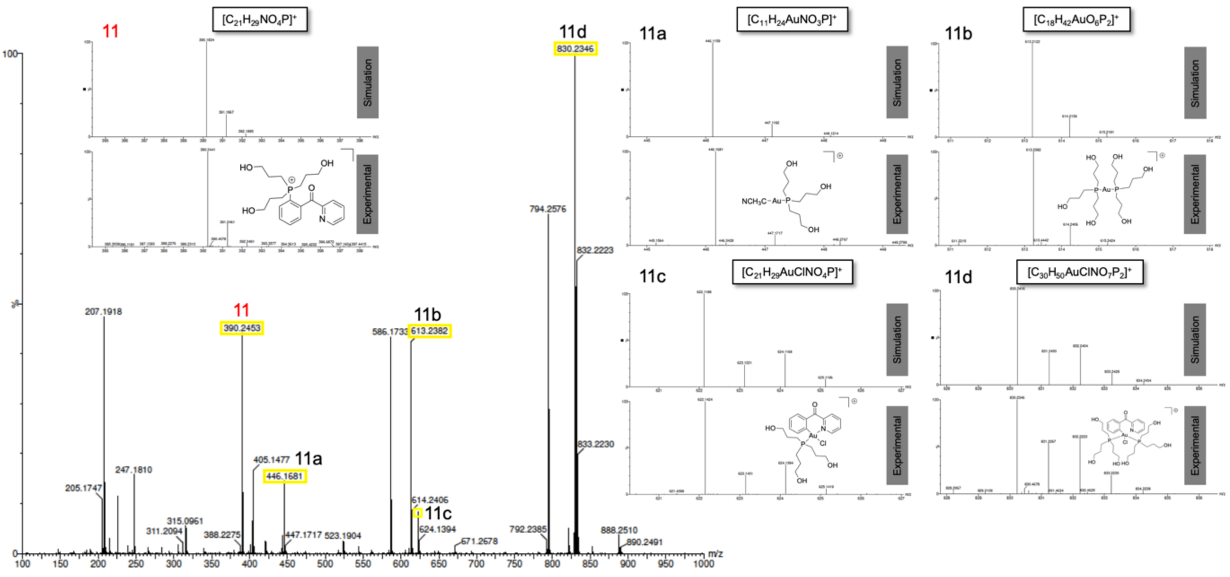

Figure S25. HR-ESI-MS spectrum of **11** and other species (**11a-d**) formed during the reaction of **1** with tris(hydroxypropyl)phosphine. Inserts show simulated (top) and experimental (bottom) spectra of **11** and **11a-d**.

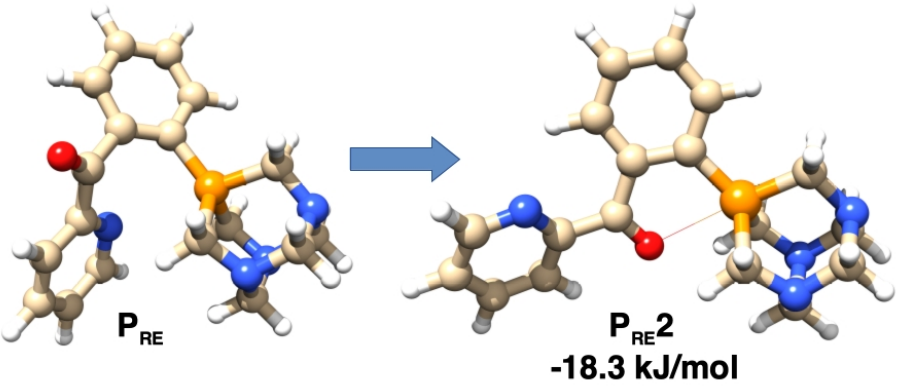

Figure S26. Conformer of the RE product, $\mathbf{P_{RE}2}$, obtained after rotation of the CO-phenyl bond and corresponding to compound **5**. Structure and energies in acetone have been obtained by DFT calculations.

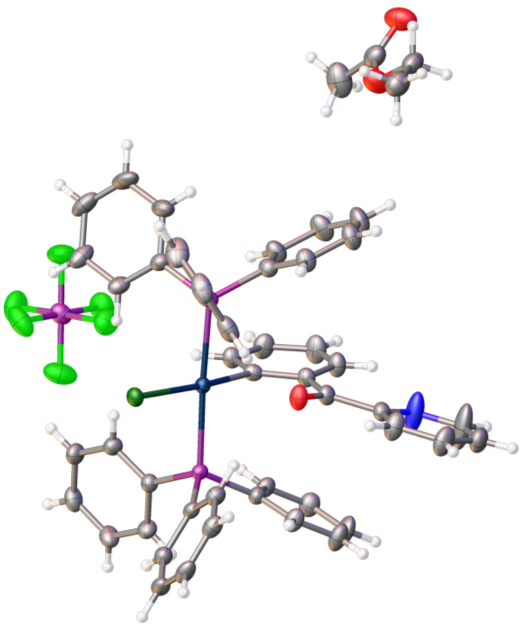

Figure S27. ORTEP plot of intermediate **I1** $[Au(C^{CO}N)Cl(triphenylphosphine)_2]^+$. Thermal ellipsoids drawn at the 50% probability level.

Table S1. Calculated relative standard Gibbs free energy values and activation barriers (kJ/mol) of the species involved in the proposed reaction pathway of the Au(III) C^N compound **1**.

**1 + PTA**	**TS0 = $E_0^‡$**	**R1 + Cl^-**				
0.0	12.9	-71.4				
**R1 + PTA**	**TS1 = $E_1^‡$**	**I1**	**TS1'**	**$E_{1'}^‡$**	**P_{RE} + Au(PTA)Cl**
0.0	0.4	-61.3.	27.9	89.2	-99.8
**R1 + PTA**	**TS00 = $E_{00}^‡$**	**R2 + Cl^-**			
0.0	8.5	-4.3			
**R2 + PTA**		**I2**	**TS2'**	**$E_{2'}^‡$**	**P_{RE} + $[Au(PTA)_2]^+$**
0.0		-45.1	10.4	55.5	-153.5

Table S2. Cartesian coordinates (Å) of the compounds reported in Figure 4, obtained by DFT calculations

1 R1 SP-4-3

C	2.549212	-0.932006	-0.210798
C	3.397817	-0.098738	0.524208
C	4.733982	-0.489762	0.729453
C	5.217680	-1.657236	0.163086
C	4.369607	-2.458991	-0.600559
C	3.028689	-2.111585	-0.769336
C	3.023892	1.227160	1.060629
H	5.372497	0.163426	1.316942
H	6.254787	-1.941768	0.308225
H	4.744542	-3.369672	-1.058760
H	2.367215	-2.769505	-1.323126
O	3.616846	1.750312	1.993389
C	2.027250	2.065603	0.301151
C	2.202583	3.446812	0.327290
C	1.419822	4.261518	-0.478323
C	0.476615	3.672032	-1.313010
C	0.313614	2.297657	-1.272173
N	1.063364	1.519871	-0.467779
H	2.973854	3.841630	0.978052
H	1.552484	5.337570	-0.465832
H	-0.141981	4.256553	-1.982891
H	-0.420775	1.789758	-1.885740
Au	0.530725	-0.475215	-0.241104
Cl	-0.057534	-2.729126	0.062862
P	-1.906900	-0.080866	-0.036430
C	-2.580220	-0.688741	1.587755
C	-2.785919	1.565447	-0.103748
C	-3.022386	-1.040046	-1.173710
H	-2.277134	-1.733403	1.714678
H	-2.136166	-0.108790	2.402796

N	-4.033780	-0.576386	1.629182
H	-2.654507	2.018960	-1.091714
N	-4.210588	1.382713	0.154142
H	-2.353149	2.243545	0.639753
H	-2.735395	-2.096106	-1.134767
N	-4.420166	-0.881563	-0.783495
H	-2.878530	-0.690107	-2.200901
C	-4.666119	-1.377880	0.575397
C	-4.842714	0.520562	-0.851002
C	-4.465809	0.816670	1.483270
H	-5.545782	0.855684	1.658641
H	-3.965193	1.431476	2.237772
H	-5.924262	0.553912	-0.683988
H	-4.629991	0.915269	-1.849407
H	-5.747416	-1.362985	0.746114
H	-4.312943	-2.411128	0.650235

1 R1 SP-4-4

C	-0.909952	1.528649	-0.469427
C	-1.953404	2.051863	0.300809
C	-2.195866	3.436328	0.263822
C	-1.456851	4.264171	-0.566617
C	-0.463806	3.719072	-1.377557
C	-0.177665	2.354821	-1.316970
C	-2.922215	1.253029	1.090101
H	-2.999920	3.826916	0.880601
H	-1.664005	5.328757	-0.595851
H	0.102011	4.351570	-2.054878
H	0.607571	1.948587	-1.948180
O	-3.513175	1.724655	2.051326
C	-3.361672	-0.092617	0.576004
C	-4.657193	-0.501614	0.879715
C	-5.154313	-1.674901	0.327577
C	-4.337207	-2.421195	-0.512942
C	-3.039450	-1.988653	-0.745804
N	-2.570995	-0.847634	-0.213960
H	-5.248376	0.126701	1.535151
H	-6.166184	-1.998373	0.546355
H	-4.682693	-3.336371	-0.978290
H	-2.341651	-2.560045	-1.347195
Au	-0.458797	-0.450555	-0.282231
Cl	0.092576	-2.844194	-0.173196
P	1.827075	-0.108282	0.005680
C	2.493965	-0.981413	1.502386
C	2.954981	-0.789550	-1.296330
C	2.585961	1.568396	0.261880
H	2.019422	-0.574469	2.400175
H	2.237025	-2.042054	1.426659
N	3.937961	-0.801807	1.563615
H	2.792425	-0.261261	-2.240651
N	4.336779	-0.629586	-0.856904
H	2.716133	-1.846978	-1.447484

H	2.114242	2.054655	1.121725
N	4.018429	1.403201	0.488753
H	2.422161	2.201411	-0.613898
C	4.305109	0.611648	1.689645
C	4.688672	0.780955	-0.658785
C	4.607072	-1.363921	0.385052
H	5.685948	-1.331038	0.564465
H	4.302082	-2.407181	0.260960
H	5.767359	0.835255	-0.483327
H	4.451842	1.344304	-1.566343
H	5.382396	0.665068	1.873807
H	3.780462	1.047063	2.545089

1 + PTA

C	1.930558	-1.331124	-0.438516
C	3.214013	-0.903341	-0.783947
C	4.041723	-1.772457	-1.519126
C	3.571518	-3.003880	-1.942795
C	2.268490	-3.391153	-1.630390
C	1.449327	-2.565506	-0.860398
C	3.765905	0.443413	-0.517934
H	5.043037	-1.430763	-1.764716
H	4.212033	-3.659017	-2.524184
H	1.884760	-4.348156	-1.971755
H	0.453891	-2.894439	-0.580491
O	4.969329	0.658330	-0.475706
C	2.832429	1.623801	-0.519069
C	3.338573	2.841073	-0.966200
C	2.485794	3.924169	-1.130663
C	1.136163	3.765469	-0.840669
C	0.690700	2.544424	-0.357250
N	1.528274	1.505033	-0.199708
H	4.396901	2.894248	-1.192500
H	2.867548	4.874042	-1.488725
H	0.425226	4.572550	-0.969260
H	-0.339623	2.374992	-0.066362
Au	0.825388	-0.181263	0.821974
Cl	-0.502000	1.248246	2.349998
Cl	0.232596	-2.073255	2.082271
P	-2.040498	0.063009	-0.861799
C	-2.813856	-1.076001	0.414963
C	-3.061918	1.567207	-0.383762
C	-3.165365	-0.466443	-2.272973
H	-2.483789	-2.103135	0.216227
H	-2.417226	-0.797491	1.399080
N	-4.277516	-1.040329	0.453803
H	-2.908691	2.351400	-1.135691
N	-4.497992	1.310288	-0.254014
H	-2.679086	1.946526	0.572735
H	-2.860888	-1.464291	-2.609783
N	-4.588841	-0.496017	-1.929427
H	-3.018310	0.218020	-3.116478

C	-4.851763	-1.434043	-0.834269
C	-5.063092	0.827333	-1.516580
C	-4.761604	0.303483	0.778454
H	-5.848144	0.244721	0.918222
H	-4.301283	0.629796	1.718410
H	-6.151859	0.769010	-1.395526
H	-4.836185	1.550245	-2.308637
H	-5.939523	-1.504928	-0.709998
H	-4.464754	-2.421586	-1.110320

1 TS0

C	-1.416396	1.491026	-0.261121
C	-2.697848	1.385830	-0.807648
C	-3.234804	2.513554	-1.463528
C	-2.505236	3.679932	-1.603043
C	-1.220970	3.756565	-1.064472
C	-0.683409	2.669765	-0.379397
C	-3.581277	0.192503	-0.804883
H	-4.237578	2.422583	-1.870189
H	-2.931649	4.528340	-2.128356
H	-0.634994	4.665840	-1.161985
H	0.294855	2.755444	0.084110
O	-4.794729	0.310420	-0.926979
C	-3.010050	-1.196299	-0.833933
C	-3.787306	-2.193266	-1.423726
C	-3.270688	-3.473635	-1.561775
C	-1.986231	-3.730510	-1.096939
C	-1.275473	-2.704637	-0.488568
N	-1.781386	-1.468863	-0.364826
H	-4.779229	-1.927747	-1.769668
H	-3.859219	-4.256705	-2.027755
H	-1.532305	-4.710238	-1.188353
H	-0.289247	-2.860759	-0.062819
Au	-0.622659	0.002724	0.860270
Cl	0.439503	-1.810290	2.202838
Cl	-0.450810	1.564728	2.752134
P	1.809054	0.015114	-0.410416
C	3.034668	0.602879	0.868330
C	2.638864	-1.617367	-0.782367
C	2.509962	0.970845	-1.859924
H	2.817421	1.647858	1.118736
H	2.885382	0.009715	1.778329
N	4.422574	0.485718	0.420647
H	2.159779	-2.073142	-1.656017
N	4.071525	-1.470262	-1.035872
H	2.479164	-2.282811	0.073989
H	2.275900	2.034455	-1.739536
N	3.958519	0.802775	-1.978389
H	2.026102	0.629414	-2.781348
C	4.659424	1.285794	-0.783916
C	4.321992	-0.601702	-2.189569
C	4.767398	-0.908081	0.126179

H	5.843689	-0.952356	-0.075861
H	4.548062	-1.523569	1.005246
H	5.394960	-0.638513	-2.409764
H	3.772739	-0.987790	-3.054825
H	5.734265	1.262727	-0.996981
H	4.361857	2.321774	-0.589890

1 R1 + Cl^-

C	-0.752659	1.501763	-0.701129
C	-1.723263	2.113243	0.097810
C	-1.855783	3.511018	0.050303
C	-1.071778	4.273767	-0.802558
C	-0.143960	3.646835	-1.630457
C	0.025174	2.262689	-1.570049
C	-2.726165	1.402781	0.934306
H	-2.607507	3.969097	0.686563
H	-1.191648	5.351933	-0.831846
H	0.462115	4.228714	-2.318598
H	0.769168	1.790890	-2.206451
O	-3.283932	1.957638	1.870687
C	-3.284097	0.084073	0.457899
C	-4.590833	-0.225118	0.826912
C	-5.206827	-1.353333	0.301601
C	-4.499344	-2.153584	-0.587233
C	-3.187325	-1.818431	-0.889870
N	-2.601670	-0.724484	-0.378113
H	-5.095650	0.444994	1.512458
H	-6.227002	-1.599360	0.576463
H	-4.940133	-3.035514	-1.036267
H	-2.569458	-2.435594	-1.532158
Au	-0.460921	-0.506868	-0.489043
Cl	-0.121435	-2.955331	-0.461965
Cl	-0.344780	-0.106517	2.566992
P	1.818777	-0.230681	-0.162801
C	2.565911	-1.463175	1.000575
C	3.036933	-0.334248	-1.556320
C	2.372167	1.339762	0.645533
H	2.008692	-1.403380	1.942023
H	2.443648	-2.466678	0.583412
N	3.977156	-1.152516	1.197696
H	2.834476	0.441043	-2.301669
N	4.387285	-0.167695	-1.018554
H	2.943048	-1.308059	-2.047741
H	1.816471	1.436567	1.584976
N	3.811089	1.280394	0.888041
H	2.134135	2.197718	0.009578
C	4.165873	0.177067	1.787344
C	4.562887	1.134191	-0.362107
C	4.728371	-1.224879	-0.058539
H	5.792638	-1.126775	0.178441
H	4.560139	-2.200897	-0.523755
H	5.626863	1.247271	-0.130542

H	4.268199	1.929220	-1.054461
H	5.224119	0.283100	2.047053
H	3.567200	0.252378	2.700377

1 R1 + PTA

C	1.988706	-0.148360	1.280312
C	3.283891	-0.674217	1.244347
C	4.156269	-0.399048	2.312386
C	3.728245	0.331091	3.410117
C	2.413038	0.788090	3.464061
C	1.547971	0.561037	2.393077
C	3.801094	-1.610376	0.217418
H	5.162762	-0.803928	2.261012
H	4.409830	0.526799	4.231190
H	2.053984	1.330912	4.333183
H	0.525360	0.923640	2.443432
O	4.997577	-1.717646	-0.012423
C	2.866031	-2.612021	-0.408126
C	3.403881	-3.841215	-0.780624
C	2.567911	-4.840747	-1.260717
C	1.207393	-4.581656	-1.373376
C	0.733998	-3.323624	-1.029599
N	1.546927	-2.369097	-0.549275
H	4.472302	-3.983168	-0.670184
H	2.972848	-5.807067	-1.541120
H	0.511957	-5.329155	-1.735333
H	-0.305945	-3.045758	-1.156367
Au	0.812232	-0.349696	-0.377446
Cl	-0.564863	-0.633908	-2.403920
P	0.391957	1.948392	-0.410733
C	-0.258051	2.639573	-2.005542
C	-0.768763	2.780652	0.783040
C	1.890285	3.018695	-0.186018
H	0.408794	2.344396	-2.820973
H	-1.243682	2.210200	-2.208801
N	-0.340302	4.090948	-1.902767
H	-0.448494	2.604988	1.814046
N	-0.785133	4.210945	0.506811
H	-1.770528	2.356846	0.665906
H	2.639616	2.746494	-0.935856
N	1.517585	4.422691	-0.326749
H	2.322982	2.837093	0.803832
C	0.968402	4.707308	-1.657538
C	0.537280	4.824596	0.685748
C	-1.266635	4.507615	-0.847695
H	-1.401073	5.590943	-0.923587
H	-2.235245	4.022229	-1.003925
H	0.411935	5.909673	0.621882
H	0.919559	4.572937	1.679762
H	0.849089	5.791326	-1.745507
H	1.675623	4.368794	-2.420175
P	-2.343213	-0.548576	0.849588

C	-3.546253	0.349515	-0.287223
C	-2.730479	-2.255725	0.166397
C	-3.534110	-0.662134	2.300457
H	-3.646575	1.391376	0.045342
H	-3.111956	0.366310	-1.295193
N	-4.882256	-0.244169	-0.348247
H	-2.246658	-3.009373	0.799734
N	-4.157065	-2.561514	0.061264
H	-2.284091	-2.320787	-0.834458
H	-3.623067	0.330857	2.757065
N	-4.870519	-1.143562	1.945967
H	-3.105355	-1.328308	3.057817
C	-5.514977	-0.257516	0.973679
C	-4.814851	-2.488949	1.369259
C	-4.827365	-1.622502	-0.841818
H	-5.858252	-1.969294	-0.982537
H	-4.315370	-1.633757	-1.811219
H	-5.845295	-2.843339	1.245404
H	-4.295626	-3.155537	2.067059
H	-6.549929	-0.597016	0.845530
H	-5.527065	0.762813	1.373286

1 TS1

C	-0.523331	1.318860	1.478129
C	-0.705345	2.700674	1.597034
C	-1.004446	3.225446	2.869387
C	-1.076230	2.409183	3.985993
C	-0.859894	1.039102	3.852978
C	-0.600578	0.492053	2.597254
C	-0.566471	3.717873	0.519907
H	-1.156021	4.298016	2.946485
H	-1.294944	2.837750	4.958462
H	-0.902667	0.386967	4.720155
H	-0.462422	-0.582828	2.501104
O	-1.174350	4.779183	0.582673
C	0.456995	3.558057	-0.569905
C	0.959037	4.719084	-1.157972
C	1.964854	4.621566	-2.110047
C	2.437459	3.362357	-2.463434
C	1.872974	2.245858	-1.860967
N	0.914403	2.347125	-0.931778
H	0.551565	5.672236	-0.842383
H	2.374915	5.515009	-2.568682
H	3.223270	3.234846	-3.198583
H	2.169879	1.235010	-2.129025
Au	-0.204570	0.467813	-0.345303
Cl	0.189771	-0.655057	-2.527247
P	-2.167945	-0.784153	-0.238789
C	-1.995329	-2.629660	-0.230652
C	-3.407473	-0.576093	1.125452
C	-3.278533	-0.583294	-1.711338
H	-1.392271	-2.928608	-1.094914

H	-1.474109	-2.947079	0.678658
N	-3.318939	-3.243014	-0.292273
H	-3.736612	0.467696	1.167025
N	-4.544488	-1.460267	0.878377
H	-2.948279	-0.820495	2.088104
H	-2.705369	-0.827017	-2.611422
N	-4.431732	-1.465495	-1.577652
H	-3.601697	0.459681	-1.783083
C	-4.032722	-2.876087	-1.520695
C	-5.222954	-1.147713	-0.384208
C	-4.145432	-2.871924	0.861715
H	-5.057778	-3.475740	0.827518
H	-3.604342	-3.105723	1.783499
H	-6.142715	-1.739251	-0.423675
H	-5.488507	-0.086582	-0.398995
H	-4.940947	-3.484415	-1.572423
H	-3.404130	-3.108067	-2.385632
P	2.309991	-0.734971	0.294091
C	3.666056	-0.925637	-0.984508
C	3.423238	-0.770718	1.800523
C	1.831804	-2.542515	0.348566
H	3.195858	-1.107553	-1.959005
H	4.226781	0.013866	-1.054322
N	4.593320	-2.014225	-0.678331
H	2.801415	-0.850516	2.699469
N	4.378488	-1.879156	1.772773
H	3.969991	0.176300	1.863495
H	1.293845	-2.778301	-0.578448
N	2.970500	-3.447812	0.496774
H	1.138165	-2.697549	1.184679
C	3.904018	-3.306924	-0.624533
C	3.699150	-3.177698	1.739736
C	5.263395	-1.795398	0.607285
H	6.034793	-2.566210	0.717767
H	5.748008	-0.813283	0.595441
H	4.461692	-3.956250	1.857397
H	3.002648	-3.239018	2.583130
H	4.666290	-4.088489	-0.526915
H	3.360376	-3.461053	-1.563103

1 I1

C	-0.020222	0.753645	1.605507
C	-0.572311	2.030255	1.443589
C	-0.742151	2.844655	2.571745
C	-0.319829	2.418535	3.824493
C	0.238161	1.151564	3.971616
C	0.372228	0.309985	2.865599
C	-1.128845	2.513473	0.145269
H	-1.203641	3.820027	2.443569
H	-0.438069	3.067537	4.686107
H	0.561836	0.803422	4.947922
H	0.775016	-0.690276	3.002103

O	-2.296042	2.897275	0.085531
C	-0.266378	2.551756	-1.072080
C	-0.857988	2.777520	-2.317900
C	-0.044191	2.847138	-3.441725
C	1.331015	2.729787	-3.272495
C	1.829005	2.535042	-1.983818
N	1.063210	2.422719	-0.897680
H	-1.933456	2.906373	-2.382361
H	-0.471929	3.005580	-4.426189
H	2.010844	2.795728	-4.114951
H	2.905516	2.475217	-1.820262
Au	0.036157	-0.551476	0.011343
Cl	0.061994	-2.085609	-1.909471
P	-2.353030	-0.681297	0.013978
C	-3.042719	-2.405569	-0.025627
C	-3.358980	0.027430	1.404664
C	-3.274518	0.041861	-1.429954
H	-2.649033	-2.919242	-0.908562
H	-2.706648	-2.949551	0.862086
N	-4.498913	-2.354426	-0.067297
H	-3.191782	1.108950	1.457853
N	-4.775220	-0.246173	1.170997
H	-3.037994	-0.416250	2.352989
H	-2.887224	-0.392971	-2.357338
N	-4.699257	-0.230304	-1.288387
H	-3.106367	1.123341	-1.453485
C	-4.984366	-1.669087	-1.269996
C	-5.248190	0.377408	-0.069707
C	-5.056119	-1.684532	1.114103
H	-6.142950	-1.812728	1.088932
H	-4.666817	-2.164757	2.017311
H	-6.337022	0.271393	-0.105133
H	-4.995254	1.442542	-0.055390
H	-6.070685	-1.795058	-1.312254
H	-4.540991	-2.135546	-2.154979
P	2.416898	-0.575371	0.088397
C	3.344090	-0.688047	-1.515813
C	3.370969	0.768928	0.934077
C	3.160939	-2.052150	0.940811
H	2.994498	-1.573434	-2.056492
H	3.124752	0.187067	-2.135202
N	4.775648	-0.779612	-1.252050
H	3.047798	0.839022	1.978496
N	4.800029	0.474053	0.867668
H	3.144898	1.727657	0.456553
H	2.808053	-2.962892	0.446074
N	4.617623	-1.980202	0.886747
H	2.826449	-2.083332	1.982437
C	5.110301	-1.990425	-0.494933
C	5.128841	-0.778951	1.557042
C	5.276394	0.388006	-0.516622
H	6.368796	0.324044	-0.492432

H	4.993311	1.299787	-1.052719
H	6.219342	-0.860894	1.602664
H	4.736922	-0.741018	2.578065
H	6.201045	-2.073680	-0.460681
H	4.708176	-2.864034	-1.016733

1 TS1'

C	0.573576	1.379433	-0.747545
C	0.504521	2.400783	0.244585
C	0.148910	3.691692	-0.171831
C	-0.128941	3.995104	-1.497005
C	-0.013701	3.003874	-2.469714
C	0.348825	1.715011	-2.101770
C	0.909862	2.300960	1.681196
H	0.111299	4.462851	0.590575
H	-0.412781	5.004886	-1.773385
H	-0.209349	3.225469	-3.513914
H	0.426405	0.946781	-2.865774
O	1.186472	3.328785	2.294303
C	1.042401	1.009196	2.430389
C	1.953588	0.961392	3.491019
C	2.027855	-0.194290	4.258577
C	1.172783	-1.251960	3.960859
C	0.294710	-1.117603	2.888285
N	0.229463	-0.017511	2.128262
H	2.577787	1.825050	3.693155
H	2.735267	-0.266877	5.078110
H	1.188787	-2.169336	4.538658
H	-0.377841	-1.926983	2.609049
Au	-0.263114	-0.615309	-0.448503
Cl	-0.090497	-3.099487	-0.484030
P	2.105787	-0.023441	-0.594300
C	2.870182	-1.352061	0.530494
C	2.727750	-0.626770	-2.244129
C	3.413253	1.302259	-0.287899
H	2.757078	-1.038309	1.573322
H	2.305861	-2.276822	0.368841
N	4.263447	-1.512797	0.197371
H	2.533117	0.131523	-3.009435
N	4.156585	-0.901799	-2.172993
H	2.180100	-1.535595	-2.511836
H	3.302796	1.678900	0.736122
N	4.742145	0.776084	-0.485953
H	3.212909	2.126657	-0.982398
C	5.053229	-0.306091	0.451742
C	4.942188	0.294030	-1.858840
C	4.466837	-1.945016	-1.190503
H	5.519865	-2.217030	-1.306998
H	3.849392	-2.826142	-1.387570
H	6.000521	0.044137	-1.976524
H	4.688615	1.093005	-2.561548
H	6.111016	-0.560126	0.340826

H	4.874529	0.034941	1.476324
P	-2.658152	-0.329416	-0.312750
C	-3.461186	-1.056796	1.199499
C	-3.347408	1.390324	-0.167327
C	-3.843178	-0.991441	-1.584452
H	-3.290994	-2.139384	1.209180
H	-2.980947	-0.636736	2.090244
N	-4.895109	-0.777781	1.232247
H	-3.103093	1.956846	-1.073106
N	-4.796885	1.371625	0.029684
H	-2.865458	1.894960	0.678560
H	-3.690218	-2.071259	-1.686651
N	-5.228220	-0.716780	-1.207421
H	-3.625907	-0.532280	-2.554268
C	-5.578805	-1.350716	0.068029
C	-5.481520	0.723980	-1.094640
C	-5.160769	0.664776	1.262546
H	-6.235676	0.802686	1.421746
H	-4.620932	1.111486	2.104016
H	-6.558839	0.863080	-0.954179
H	-5.183211	1.214881	-2.026670
H	-6.656427	-1.224714	0.218138
H	-5.355241	-2.420942	0.011257

1 P_{RE} + Au(PTA)Cl

C	3.771556	1.302776	-0.648506
C	2.926972	2.089920	0.161867
C	2.790130	3.448800	-0.140035
C	3.408052	4.011731	-1.249409
C	4.209371	3.223918	-2.069635
C	4.409049	1.885230	-1.756113
C	2.159464	1.636069	1.365333
H	2.157947	4.049832	0.505447
H	3.265239	5.064326	-1.470828
H	4.695846	3.648217	-2.941451
H	5.068305	1.295049	-2.385996
O	2.205852	2.297013	2.394672
C	1.263412	0.445524	1.273716
C	0.515369	0.081994	2.396191
C	-0.325779	-1.019190	2.306592
C	-0.400475	-1.701433	1.094851
C	0.378519	-1.256618	0.026227
N	1.210173	-0.213601	0.102615
H	0.614786	0.662857	3.306433
H	-0.910280	-1.340603	3.163167
H	-1.035385	-2.575145	0.978371
H	0.339481	-1.773028	-0.932374
Au	-2.240117	1.371401	0.025870
Cl	-0.666876	3.121750	0.254151
P	4.260991	-0.395243	-0.321616
C	4.069093	-1.201221	1.336659
C	3.701352	-1.732902	-1.465705

C	6.094717	-0.553135	-0.521543
H	4.529538	-0.572408	2.105773
H	3.015314	-1.339106	1.586907
N	4.729073	-2.499580	1.278649
H	3.921075	-1.445431	-2.499454
N	4.410478	-2.958086	-1.115266
H	2.621618	-1.869562	-1.370794
H	6.589705	0.100262	0.204297
N	6.502007	-1.938314	-0.319615
H	6.390316	-0.223418	-1.522583
C	6.171703	-2.396103	1.033200
C	5.863759	-2.834056	-1.291059
C	4.137362	-3.380331	0.263636
H	4.566530	-4.377166	0.398836
H	3.056056	-3.442961	0.420401
H	6.301000	-3.829011	-1.167792
H	6.075711	-2.480391	-2.304173
H	6.604729	-3.391260	1.168676
H	6.615929	-1.716570	1.766098
P	-3.800277	-0.234160	-0.209628
C	-5.483884	0.245789	-0.838174
C	-3.488694	-1.658128	-1.364240
C	-4.343524	-1.196155	1.285449
H	-5.916018	0.995745	-0.167930
H	-5.378680	0.705091	-1.826303
N	-6.368686	-0.913976	-0.921275
H	-2.579464	-2.187481	-1.059822
N	-4.618252	-2.584389	-1.376596
H	-3.317246	-1.266539	-2.372445
H	-4.736078	-0.499194	2.033277
N	-5.365899	-2.179778	0.937134
H	-3.480220	-1.701608	1.731025
C	-6.566533	-1.537231	0.390906
C	-4.868679	-3.148842	-0.045734
C	-5.845433	-1.928262	-1.841616
H	-6.613049	-2.700497	-1.959693
H	-5.658343	-1.467401	-2.816872
H	-5.624512	-3.934025	-0.153111
H	-3.944752	-3.600365	0.332002
H	-7.337886	-2.307266	0.282851
H	-6.922166	-0.781254	1.098350

1 TS00

C	-0.502684	1.391001	1.429492
C	-0.633476	2.764695	1.657625
C	-0.928061	3.211246	2.957654
C	-1.024176	2.313959	4.010442
C	-0.811081	0.956108	3.781872
C	-0.564714	0.492571	2.488605
C	-0.352181	3.825582	0.660127
H	-1.040966	4.280091	3.114398
H	-1.239842	2.673045	5.011267

H	-0.841931	0.248475	4.604894
H	-0.395181	-0.567482	2.317432
O	-0.857929	4.936276	0.733740
C	0.760395	3.612726	-0.334176
C	1.486570	4.731090	-0.734406
C	2.598323	4.570902	-1.551338
C	2.952061	3.291645	-1.963740
C	2.163338	2.219091	-1.573076
N	1.099650	2.382595	-0.769805
H	1.164129	5.702306	-0.378222
H	3.181614	5.431990	-1.858958
H	3.815910	3.113768	-2.592679
H	2.352965	1.208420	-1.917768
Au	-0.292609	0.761941	-0.504249
Cl	0.028713	0.102343	-2.848846
P	-2.068593	-0.749253	-0.302109
C	-2.148169	-2.129163	0.949746
C	-3.706808	0.055213	0.034548
C	-2.489601	-1.696338	-1.839828
H	-1.294829	-2.798624	0.798263
H	-2.092559	-1.719709	1.961703
N	-3.407172	-2.841768	0.787015
H	-3.894226	0.823176	-0.721961
N	-4.765060	-0.951073	0.005253
H	-3.675973	0.546808	1.013271
H	-1.653207	-2.354285	-2.096427
N	-3.705854	-2.467813	-1.617072
H	-2.622679	-0.991487	-2.665090
C	-3.539199	-3.447769	-0.541861
C	-4.853597	-1.608672	-1.304404
C	-4.566527	-1.976130	1.033235
H	-5.458087	-2.610187	1.053453
H	-4.456124	-1.494918	2.009661
H	-5.750376	-2.235749	-1.303310
H	-4.957984	-0.848599	-2.084142
H	-4.425022	-4.090284	-0.529084
H	-2.659757	-4.065381	-0.746775
P	1.537883	-1.949172	0.371562
C	2.572367	-2.155260	-1.181262
C	2.782999	-0.858562	1.266043
C	2.110438	-3.549554	1.177420
H	2.111522	-2.928558	-1.807462
H	2.529692	-1.221931	-1.756504
N	3.970323	-2.510950	-0.933079
H	2.465023	-0.747496	2.310089
N	4.157569	-1.359839	1.238300
H	2.761606	0.144646	0.819734
H	1.627506	-4.390180	0.665591
N	3.560173	-3.749042	1.158163
H	1.762205	-3.560878	2.217005
C	4.072736	-3.784249	-0.214212
C	4.252890	-2.675875	1.876019

C	4.647664	-1.484403	-0.137375
H	5.711730	-1.745738	-0.090082
H	4.547453	-0.515679	-0.641003
H	5.315013	-2.942967	1.933807
H	3.851342	-2.609611	2.893571
H	5.133683	-4.058193	-0.168036
H	3.534993	-4.555675	-0.776659

1 R1 + Cl$^-$

C	2.024504	1.408393	-0.739826
C	2.368447	2.392175	0.191788
C	3.682410	2.887097	0.205346
C	4.613563	2.447061	-0.725425
C	4.238925	1.517552	-1.693243
C	2.947978	0.983834	-1.689834
C	1.401360	3.085527	1.085767
H	3.935425	3.643639	0.942785
H	5.624533	2.841034	-0.708872
H	4.951024	1.189738	-2.444850
H	2.678183	0.234589	-2.429450
O	1.753056	3.663840	2.101965
C	0.021087	3.368545	0.534551
C	-0.583338	4.562771	0.917633
C	-1.740448	4.988162	0.277291
C	-2.255734	4.219745	-0.760031
C	-1.633286	3.019601	-1.071055
N	-0.538784	2.596686	-0.420753
H	-0.106377	5.142517	1.699282
H	-2.218555	5.917406	0.567580
H	-3.131617	4.525791	-1.319331
H	-2.003080	2.374155	-1.859072
Au	0.154878	0.538387	-0.506613
P	1.295480	-1.485063	-0.186918
C	1.833981	-2.564779	-1.600574
C	2.898587	-1.339770	0.744417
C	0.469374	-2.747197	0.896482
H	0.964703	-2.884632	-2.183368
H	2.495864	-2.005289	-2.268732
N	2.539140	-3.724675	-1.063100
H	2.675882	-0.847466	1.697746
N	3.465188	-2.665696	0.953138
H	3.600484	-0.716902	0.182799
H	-0.480416	-3.071825	0.457143
N	1.346776	-3.894708	1.079683
H	0.267951	-2.256533	1.856008
C	1.682858	-4.539368	-0.194028
C	2.589226	-3.515695	1.762533
C	3.744681	-3.342018	-0.316848
H	4.304433	-4.256411	-0.097281
H	4.363380	-2.691355	-0.942760
H	3.134043	-4.434457	2.000067
H	2.343712	-2.997825	2.694546

H	2.220864	-5.465198	0.031560
H	0.761074	-4.792223	-0.726929
P	-2.114685	-0.452478	-0.257606
C	-2.618394	-1.065170	1.430792
C	-3.641581	0.582831	-0.603035
C	-2.614320	-1.935691	-1.277662
H	-1.904642	-1.813768	1.784790
H	-2.560616	-0.225057	2.129426
N	-3.963541	-1.626202	1.406560
H	-3.630915	0.906348	-1.649055
N	-4.860478	-0.173372	-0.354150
H	-3.623065	1.474711	0.032988
H	-1.910630	-2.761909	-1.128320
N	-3.960802	-2.381407	-0.937172
H	-2.569498	-1.663538	-2.337587
C	-4.073061	-2.755518	0.477919
C	-4.957657	-1.347802	-1.226395
C	-4.959064	-0.617383	1.039913
H	-5.953239	-1.053773	1.180750
H	-4.857524	0.247148	1.703077
H	-5.950273	-1.787481	-1.085322
H	-4.858085	-1.032146	-2.269791
H	-5.055564	-3.216067	0.623124
H	-3.303159	-3.496480	0.717681
Cl	0.293582	0.418898	2.492685

1 R1 + PTA

C	-0.746437	-2.165839	0.776295
C	-1.117543	-2.625170	2.042838
C	-1.622127	-3.929067	2.179364
C	-1.700253	-4.771757	1.079498
C	-1.275813	-4.322440	-0.169577
C	-0.820369	-3.011150	-0.326273
C	-0.918083	-1.856777	3.297547
H	-1.920139	-4.263700	3.168916
H	-2.080419	-5.781122	1.195511
H	-1.310425	-4.981407	-1.031762
H	-0.528087	-2.665235	-1.315690
O	-1.624118	-2.014359	4.281766
C	0.348143	-1.048780	3.431024
C	0.949866	-1.002648	4.685897
C	2.213990	-0.442437	4.821445
C	2.855829	0.047995	3.689840
C	2.183609	0.012187	2.476913
N	0.953507	-0.505887	2.353129
H	0.415697	-1.434879	5.524057
H	2.698460	-0.407668	5.791012
H	3.855663	0.462557	3.733688
H	2.628822	0.395450	1.563188
Au	-0.280650	-0.142605	0.614743
P	-2.281382	0.013829	-0.626348
C	-2.213437	-0.375548	-2.443944

C	-3.679181	-1.115700	-0.128341
C	-3.228716	1.617933	-0.670841
H	-1.518434	0.302289	-2.949790
H	-1.839844	-1.395832	-2.578498
N	-3.548275	-0.260782	-3.015631
H	-3.957122	-0.907006	0.909736
N	-4.815902	-0.909792	-1.014329
H	-3.342269	-2.155068	-0.186034
H	-2.603322	2.420752	-1.076564
N	-4.417576	1.460249	-1.493888
H	-3.514583	1.887899	0.351211
C	-4.089163	1.095330	-2.876528
C	-5.334177	0.457949	-0.939035
C	-4.485404	-1.214724	-2.410198
H	-5.413203	-1.187528	-2.988983
H	-4.063682	-2.222605	-2.470689
H	-6.263892	0.500343	-1.514038
H	-5.553951	0.707455	0.102669
H	-5.009422	1.152662	-3.464807
H	-3.370537	1.815747	-3.278818
P	0.284193	2.260251	0.403887
C	-0.747774	3.605257	1.190285
C	1.962853	2.872857	0.969185
C	0.349965	2.936365	-1.334705
H	-1.786298	3.522529	0.856020
H	-0.742601	3.465407	2.275654
N	-0.226528	4.923587	0.851668
H	2.745281	2.294256	0.464513
N	2.133987	4.284253	0.665015
H	2.062053	2.716277	2.048401
H	-0.624393	2.819948	-1.821942
N	0.724275	4.343030	-1.341665
H	1.073881	2.351246	-1.913477
C	-0.235040	5.165609	-0.594876
C	2.060159	4.547246	-0.775560
C	1.137760	5.114853	1.349647
H	1.410890	6.162853	1.191565
H	1.166413	4.903206	2.422740
H	2.340283	5.592049	-0.940459
H	2.776273	3.903567	-1.296953
H	0.026164	6.215462	-0.759618
H	-1.242828	4.992987	-0.986989
P	2.135683	-0.691750	-1.107072
C	1.696071	-1.142118	-2.875685
C	3.827205	0.036380	-1.509212
C	2.772355	-2.384105	-0.604590
H	0.784822	-1.752458	-2.878244
H	1.466605	-0.222903	-3.427416
N	2.755762	-1.870057	-3.566078
H	4.367638	0.219420	-0.572749
N	4.632024	-0.830355	-2.362741
H	3.687776	1.004961	-2.004510

H	1.920867	-3.060105	-0.463659
N	3.699981	-2.964924	-1.571582
H	3.273812	-2.294617	0.366492
C	3.071041	-3.130305	-2.885179
C	4.891446	-2.127550	-1.730173
C	3.980980	-1.070343	-3.653536
H	4.689817	-1.610631	-4.290774
H	3.748338	-0.108690	-4.123339
H	5.603332	-2.669966	-2.362373
H	5.347627	-1.963535	-0.748023
H	3.765354	-3.689756	-3.521861
H	2.151154	-3.715171	-2.769614

1 I2

C	0.271267	-2.234260	-0.133133
C	0.169677	-2.942124	1.076259
C	0.373541	-4.333501	1.056687
C	0.686189	-5.002499	-0.116817
C	0.733682	-4.297409	-1.317820
C	0.510415	-2.920060	-1.325221
C	-0.349082	-2.382214	2.357918
H	0.267620	-4.872962	1.993228
H	0.864240	-6.072619	-0.102274
H	0.939469	-4.811602	-2.251838
H	0.524055	-2.387188	-2.273598
O	-1.053951	-3.084300	3.076838
C	-0.061337	-0.985043	2.812132
C	-1.027059	-0.318902	3.572231
C	-0.718526	0.930306	4.095277
C	0.555780	1.447344	3.874440
C	1.453541	0.704288	3.110882
N	1.159448	-0.478970	2.558482
H	-1.987820	-0.792618	3.747036
H	-1.449417	1.483798	4.676153
H	0.852988	2.406894	4.284573
H	2.464046	1.074180	2.941657
Au	0.040188	-0.147367	-0.263251
P	-2.312050	-0.710948	-0.442184
C	-2.767175	-0.968116	-2.230223
C	-2.954883	-2.328862	0.241348
C	-3.721937	0.392824	0.086828
H	-2.577949	-0.051593	-2.796976
H	-2.127360	-1.754174	-2.645316
N	-4.166741	-1.355327	-2.342010
H	-2.917224	-2.310599	1.335163
N	-4.325060	-2.543702	-0.199924
H	-2.315558	-3.146184	-0.107134
H	-3.611642	1.384696	-0.360972
N	-4.984898	-0.189241	-0.338793
H	-3.702461	0.501668	1.176593
C	-5.064069	-0.330784	-1.798019
C	-5.226446	-1.494989	0.282751

C	-4.431237	-2.626636	-1.660101
H	-5.452254	-2.934141	-1.904690
H	-3.736647	-3.386203	-2.031359
H	-6.252015	-1.794553	0.046977
H	-5.128463	-1.402889	1.368596
H	-6.090039	-0.614613	-2.050454
H	-4.841574	0.633341	-2.265981
P	-0.270804	2.286483	-0.342438
C	-1.309029	2.995060	1.036362
C	1.209935	3.408097	-0.150134
C	-1.059392	3.175309	-1.789192
H	-2.315001	2.565739	1.009652
H	-0.860208	2.706524	1.992342
N	-1.390728	4.445441	0.937114
H	1.870128	3.262295	-1.012409
N	0.813616	4.806946	-0.082650
H	1.756508	3.135691	0.759881
H	-2.049883	2.764379	-2.005706
N	-1.172202	4.599824	-1.507055
H	-0.439030	3.019932	-2.676968
C	-2.000505	4.872541	-0.327078
C	0.136593	5.230348	-1.311376
C	-0.069532	5.070250	1.058112
H	-0.213504	6.152838	1.127677
H	0.413168	4.720627	1.976817
H	-0.017528	6.312368	-1.255384
H	0.777193	5.011712	-2.170998
H	-2.157316	5.954314	-0.274047
H	-2.974027	4.386571	-0.444275
P	2.482227	-0.262092	-0.437571
C	3.052055	-0.595800	-2.181125
C	3.706968	1.091465	-0.013374
C	3.325693	-1.687558	0.421971
H	2.540981	-1.484743	-2.562900
H	2.778290	0.249802	-2.820201
N	4.494635	-0.808503	-2.215080
H	3.630918	1.342785	1.048519
N	5.056188	0.650898	-0.325691
H	3.478667	1.989565	-0.592970
H	2.828644	-2.620321	0.141685
N	4.729710	-1.747889	0.043929
H	3.210949	-1.552784	1.501587
C	4.897353	-1.958944	-1.397718
C	5.450665	-0.537033	0.438402
C	5.227230	0.374036	-1.755767
H	6.292869	0.204214	-1.937939
H	4.908122	1.246691	-2.333525
H	6.518487	-0.703501	0.266996
H	5.290865	-0.351819	1.505159
H	5.956795	-2.152844	-1.590898
H	4.318032	-2.837096	-1.701703

1 TS2’

C	-0.466065	-2.003300	-0.844637
C	-0.357179	-3.127886	0.018582
C	0.008183	-4.357330	-0.548548
C	0.270451	-4.496708	-1.904805
C	0.132010	-3.398607	-2.750623
C	-0.247757	-2.167493	-2.226291
C	-0.750366	-3.194790	1.460796
H	0.068038	-5.212199	0.117313
H	0.561779	-5.462408	-2.303509
H	0.318212	-3.491412	-3.815650
H	-0.356855	-1.317250	-2.893478
O	-1.042482	-4.279797	1.953241
C	-0.841046	-1.990264	2.344514
C	-1.752420	-2.011184	3.405320
C	-1.775649	-0.938932	4.288604
C	-0.858598	0.093693	4.110777
C	0.027144	0.018519	3.039858
N	0.032370	-0.986383	2.154673
H	-2.418238	-2.860440	3.517773
H	-2.486007	-0.914662	5.108218
H	-0.826016	0.942965	4.784450
H	0.771342	0.796814	2.876164
Au	0.154565	0.071818	-0.337326
P	-2.159579	-0.723170	-0.539056
C	-3.099991	0.407906	0.661584
C	-2.892916	-0.128997	-2.151105
C	-3.274630	-2.240724	-0.320846
H	-2.911356	0.075067	1.688586
H	-2.694274	1.418763	0.549826
N	-4.508624	0.390739	0.357549
H	-2.615590	-0.828182	-2.946756
N	-4.342372	-0.052378	-2.049038
H	-2.474271	0.849473	-2.403487
H	-3.105418	-2.676382	0.669411
N	-4.665304	-1.892998	-0.473511
H	-2.975442	-2.977025	-1.075768
C	-5.111979	-0.933091	0.538044
C	-4.953343	-1.362239	-1.809980
C	-4.790284	0.870111	-1.001880
H	-5.873467	0.986224	-1.097888
H	-4.321935	1.848153	-1.145487
H	-6.037193	-1.253113	-1.905144
H	-4.602156	-2.071779	-2.564405
H	-6.196904	-0.826525	0.454283
H	-4.868823	-1.311386	1.535990
P	0.079146	2.460446	-0.156755
C	-0.876522	3.468708	-1.404536
C	-0.514217	3.307281	1.400287
C	1.712535	3.343443	-0.318913
H	-0.519345	3.217739	-2.408855
H	-1.939881	3.209262	-1.357049

N	-0.713769	4.894866	-1.157675
H	0.087520	2.962540	2.247795
N	-0.400967	4.753898	1.276830
H	-1.554901	3.031424	1.603625
H	2.155058	3.092209	-1.289256
N	1.552731	4.788656	-0.213756
H	2.390533	2.991297	0.467516
C	0.686949	5.312813	-1.275047
C	0.989979	5.177313	1.083273
C	-1.209223	5.275358	0.169262
H	-1.196938	6.367913	0.232285
H	-2.241570	4.929008	0.281814
H	1.019288	6.269676	1.147422
H	1.608750	4.762168	1.885026
H	0.716324	6.405653	-1.222878
H	1.076939	4.997097	-2.247499
P	2.591278	-0.423543	-0.289008
C	3.401231	-0.428793	1.390909
C	3.047542	-2.160341	-0.785706
C	3.931744	0.474354	-1.230110
H	3.379200	0.582620	1.811618
H	2.817187	-1.078345	2.052090
N	4.779857	-0.900120	1.315632
H	2.782204	-2.316428	-1.837318
N	4.470467	-2.424083	-0.591012
H	2.458030	-2.862786	-0.184284
H	3.948180	1.528258	-0.933210
N	5.239286	-0.118705	-0.972038
H	3.708220	0.434392	-2.301267
C	5.597337	-0.045240	0.448468
C	5.297471	-1.520964	-1.397259
C	4.855543	-2.276728	0.816429
H	5.892972	-2.612295	0.916592
H	4.217591	-2.919036	1.431806
H	6.337450	-1.852800	-1.312071
H	4.991303	-1.591758	-2.445734
H	6.639419	-0.366295	0.547544
H	5.516193	0.992769	0.786859

1 P_{RE} + $[Au(PTA)_2]^+$

C	2.442374	-0.733077	1.727122
C	1.058412	-1.005051	1.674960
C	0.231467	-0.454393	2.662586
C	0.735064	0.381060	3.652596
C	2.099532	0.650757	3.698885
C	2.946152	0.083972	2.753556
C	0.338593	-1.890603	0.701586
H	-0.829664	-0.687170	2.628282
H	0.065158	0.807552	4.391809
H	2.511486	1.291193	4.471208
H	4.009648	0.292025	2.820185
O	-0.422745	-2.747295	1.135217

C	0.424535	-1.635201	-0.764614
C	-0.402665	-2.362680	-1.624177
C	-0.386635	-2.059344	-2.979775
C	0.464889	-1.053835	-3.426776
C	1.277142	-0.404314	-2.497443
N	1.264189	-0.672652	-1.188485
H	-1.038829	-3.140341	-1.212538
H	-1.023219	-2.597454	-3.674873
H	0.512301	-0.779529	-4.474664
H	1.973951	0.366681	-2.826918
Au	-1.890656	0.922185	-0.017051
P	3.701118	-1.403127	0.630296
C	3.463512	-2.960403	-0.345425
C	4.463711	-0.274539	-0.620207
C	5.209343	-1.872487	1.597354
H	3.174598	-3.773815	0.328102
H	2.681627	-2.840201	-1.098078
N	4.729540	-3.265656	-1.000073
H	4.799302	0.646169	-0.130222
N	5.583631	-0.972490	-1.236375
H	3.718462	-0.007294	-1.372633
H	4.944885	-2.653591	2.316972
N	6.248051	-2.344646	0.690423
H	5.578310	-1.010207	2.160984
C	5.813247	-3.534288	-0.048003
C	6.638725	-1.307325	-0.270279
C	5.156763	-2.201707	-1.917273
H	6.007949	-2.579096	-2.490808
H	4.342443	-1.970736	-2.611024
H	7.499857	-1.677989	-0.832915
H	6.934266	-0.403214	0.269543
H	6.669282	-3.910797	-0.615069
H	5.493803	-4.305808	0.658023
P	-0.282277	2.603001	-0.199575
C	-0.605136	4.240766	0.629479
C	1.439792	2.325687	0.443436
C	0.157797	3.248293	-1.890117
H	-1.537792	4.664205	0.243511
H	-0.732398	4.077166	1.704560
N	0.494738	5.171512	0.396063
H	1.860115	1.447923	-0.059238
N	2.287853	3.493928	0.233162
H	1.379461	2.100403	1.513814
H	-0.745602	3.638197	-2.369692
N	1.165221	4.301128	-1.805300
H	0.527336	2.423465	-2.508423
C	0.677623	5.448913	-1.032219
C	2.409203	3.824566	-1.191031
C	1.760686	4.668911	0.938117
H	2.502118	5.470492	0.855532
H	1.626715	4.420767	1.996182
H	3.153763	4.621919	-1.286800

H	2.768417	2.944911	-1.736101
H	1.415298	6.252803	-1.125418
H	-0.271191	5.791148	-1.457440
P	-3.666090	-0.591657	0.080923
C	-5.344006	0.093532	0.516252
C	-4.145700	-1.471625	-1.489296
C	-3.671765	-2.056840	1.231103
H	-5.302022	0.529276	1.519591
H	-5.597266	0.895189	-0.184965
N	-6.365666	-0.947242	0.469560
H	-3.304149	-2.081200	-1.834937
N	-5.315954	-2.318127	-1.284028
H	-4.354637	-0.727440	-2.264836
H	-3.574091	-1.700616	2.262186
N	-4.903629	-2.826286	1.089550
H	-2.804227	-2.689418	1.016369
C	-6.086308	-2.025138	1.423523
C	-5.064437	-3.349505	-0.271249
C	-6.483675	-1.532353	-0.869954
H	-7.352286	-2.199196	-0.868539
H	-6.654950	-0.733392	-1.598283
H	-5.921142	-4.031498	-0.268547
H	-4.166739	-3.912521	-0.548457
H	-6.952248	-2.695321	1.433827
H	-5.960786	-1.597961	2.423605

1 P_{RE}

C	-0.626860	1.641506	-0.219900
C	-1.903583	1.350510	0.298191
C	-2.892679	2.333663	0.229702
C	-2.657004	3.554060	-0.388149
C	-1.405933	3.826573	-0.926470
C	-0.390909	2.885619	-0.821076
C	-2.331895	0.069282	0.949287
H	-3.865674	2.113256	0.654836
H	-3.449594	4.291360	-0.445419
H	-1.208609	4.774613	-1.412934
H	0.590216	3.129887	-1.215113
O	-2.908363	0.098859	2.021971
C	-2.167979	-1.215030	0.205703
C	-2.696795	-2.384578	0.750793
C	-2.575554	-3.564293	0.030888
C	-1.939813	-3.528083	-1.203907
C	-1.437142	-2.312362	-1.662303
N	-1.537328	-1.171346	-0.979923
H	-3.187780	-2.344447	1.715563
H	-2.972880	-4.493800	0.422626
H	-1.829928	-4.422145	-1.806014
H	-0.930834	-2.257951	-2.623535
P	0.826727	0.600428	-0.043978
C	0.918921	-0.788530	1.178061
C	1.582253	-0.162190	-1.544976

C	2.246662	1.625391	0.549811
H	0.648019	-0.420086	2.171888
H	0.234850	-1.594664	0.911101
N	2.287690	-1.288799	1.172811
H	1.733127	0.605880	-2.308673
N	2.854848	-0.758408	-1.157908
H	0.908407	-0.918784	-1.949502
H	1.993586	2.046339	1.526821
N	3.447402	0.805729	0.643469
H	2.414522	2.457956	-0.138703
C	3.266991	-0.285841	1.605836
C	3.813926	0.235494	-0.658443
C	2.686821	-1.805148	-0.142798
H	3.647226	-2.312142	-0.022766
H	1.949749	-2.534700	-0.488203
H	4.780813	-0.260820	-0.547314
H	3.915794	1.039790	-1.390942
H	4.228135	-0.790648	1.729102
H	2.961992	0.127326	2.570425

1 P$_{RE}$2

C	0.083632	1.544060	-0.078721
C	-1.289478	1.217198	-0.203385
C	-2.225561	2.250269	-0.280649
C	-1.829034	3.581111	-0.221462
C	-0.488016	3.898358	-0.065588
C	0.463932	2.885361	0.007349
C	-1.669071	-0.206331	-0.347479
H	-3.271770	2.004554	-0.395265
H	-2.571522	4.367233	-0.295284
H	-0.170105	4.932871	-0.005453
H	1.504235	3.161904	0.125314
O	-0.800349	-1.017787	-0.663451
C	-3.058464	-0.689900	-0.111311
C	-3.452872	-1.889201	-0.707579
C	-4.727715	-2.369756	-0.447452
C	-5.546426	-1.649437	0.413002
C	-5.056034	-0.471162	0.971871
N	-3.842522	0.016239	0.717951
H	-2.764762	-2.415558	-1.358071
H	-5.074460	-3.290408	-0.903227
H	-6.547727	-1.989043	0.651258
H	-5.670807	0.108047	1.656470
P	1.410843	0.324224	-0.024758
C	1.415166	-1.040594	1.227817
C	1.976519	-0.545818	-1.557549
C	2.992621	1.165631	0.438240
H	1.273357	-0.612901	2.224042
H	0.598755	-1.732371	1.023201
N	2.696449	-1.726462	1.143255
H	2.193692	0.196180	-2.330461
N	3.173995	-1.305764	-1.230489
H	1.183508	-1.204440	-1.910128
H	2.884402	1.654812	1.411312
N	4.081968	0.190855	0.489402

H	3.235671	1.935641	-0.299826
C	3.824697	-0.853651	1.485300
C	4.288614	-0.447425	-0.814451
C	2.928507	-2.307770	-0.185385
H	3.812347	-2.947095	-0.120744
H	2.069382	-2.921873	-0.467067
H	5.183804	-1.070491	-0.744656
H	4.454762	0.320628	-1.574108
H	4.720009	-1.476542	1.556745
H	3.642032	-0.391943	2.458687

Sample ID: compound **5**

Crystal Data. $C_{18}H_{20}F_6N_4OP_2$, M_r = 484.32, monoclinic, $P2_1/n$ (No. 14), a = 10.8799(2) Å, b = 8.66890(10) Å, c = 21.0886(3) Å, β = 103.684(2)°, α = γ = 90°, V = 1932.55(5) Å^3, T = 100.00(10) K, Z = 4, Z' = 1, μ(CuK$_\alpha$) = 2.768 mm^{-1}, 32858 reflections measured, 3540 unique (R_{int} = 0.0618) which were used in all calculations. The final wR_2 was 0.1302 (all data) and R_1 was 0.0481 (I > 2(I)).

**Compound**	**5**
Formula	$C_{18}H_{20}F_6N_4OP_2$
$D_{calc.}$/ g cm^{-3}	1.665
μ/mm^{-1}	2.768
Formula Weight	484.32
Colour	colourless
Shape	needle
Size/mm^3	0.200×0.080×0.040
T/K	100.00(10)
Crystal System	monoclinic
Space Group	$P2_1/n$
a/Å	10.8799(2)
b/Å	8.66890(10)
c/Å	21.0886(3)
α/°	90
β/°	103.684(2)
γ/°	90
V/Å^3	1932.55(5)
Z	4
Z'	1
Wavelength/Å	1.54184
Radiation type	CuK$_\alpha$
Θ_{min}/°	4.228
Θ_{max}/°	68.249
Measured Refl.	32858
Independent Refl.	3540
Reflections with I > 2(I)	3401
R_{int}	0.0618
Parameters	280
Restraints	0
Largest Peak	0.762
Deepest Hole	-0.449
GooF	1.089
wR_2 (all data)	0.1302
wR_2	0.1283
R_1 (all data)	0.0498
R_1	0.0481

Structure Quality Indicators

**Reflections:**	d min (Cu) 0.83	I/σ 38.2	Rint 6.18%	complete 100% (IUCr) 100%
**Refinement:**	Shift 0.001	Max Peak 0.8	Min Peak -0.5	GooF 1.089

A colourless needle-shaped crystal with dimensions 0.200×0.080×0.040 mm³ was mounted on a MITIGEN holder in perfluoroether oil. Data were collected using a Rigaku 007HF diffractometer equipped with Varimax confocal mirrors and an AFC11 goniometer and HyPix 6000HE detector, and equipped with an Oxford Cryosystems low-temperature device operating at T = 100.00(10) K.

Data were measured using ω scans of 0.5 ° per frame for 0.5 to 2 s using CuK_α radiation (Rotating-anode X-ray tube, 40 kV, 30 mA). The total number of runs and images was based on the strategy calculation from the program **CrysAlisPro** (Rigaku, V1.171.39.46, 2018). The maximum resolution that was achieved was Θ = 68.249° (0.83 Å).

Cell parameters were retrieved using the **CrysAlisPro** (Rigaku, V1.171.39.46, 2018) software and refined using **CrysAlisPro** (Rigaku, V1.171.39.46, 2018) on 34573 reflections, 39% of the observed reflections.

Data reduction, scaling and absorption corrections were performed using **CrysAlisPro** (Rigaku, V1.171.39.46, 2018). The final completeness is 99.80 % out to 68.249° in Θ.

A multi-scan absorption correction was performed using CrysAlisPro 1.171.39.46 (Rigaku Oxford Diffraction, 2018) using spherical harmonics as implemented in SCALE3 ABSPACK.. The absorption coefficient μ of this material is 2.768 mm^{-1} at this wavelength (λ = 1.54184 Å) and the minimum and maximum transmissions are 0.540 and 1.000.

The structure was solved and the space group $P2_1/n$ (# 14) determined by the **ShelXT** (Sheldrick, 2015) structure solution program using Intrinsic Phasing and refined by Least Squares using version 2014/7 of **ShelXL** (Sheldrick, 2015). All non-hydrogen atoms were refined anisotropically. Hydrogen atom positions were calculated geometrically and refined using the riding model.

There is a single molecule in the asymmetric unit, which is represented by the reported sum formula. In other words: Z is 4 and Z' is 1.

Citations

CrysAlisPro Software System, Rigaku Oxford Diffraction, (2018).

O.V. Dolomanov and L.J. Bourhis and R.J. Gildea and J.A.K. Howard and H. Puschmann, Olex2: A complete structure solution, refinement and analysis program, *J. Appl. Cryst.*, (2009), **42**, 339-341.

Sheldrick, G.M., Crystal structure refinement with ShelXL, *Acta Cryst.*, (2015), **C27**, 3-8.

Sheldrick, G.M., ShelXT-Integrated space-group and crystal-structure determination, *Acta Cryst.*, (2015), **A71**, 3-8.

Reflection Statistics

Total reflections (after filtering)	34175	Unique reflections	3540
Completeness	0.998	Mean I/σ	29.42
hkl_{max} collected	(13, 9, 25)	hkl_{min} collected	(-13, -10, -25)
hkl_{max} used	(12, 10, 25)	hkl_{min} used	(-13, 0, 0)
Lim d_{max} collected	100.0	Lim d_{min} collected	0.77
d_{max} used	10.57	d_{min} used	0.83
Friedel pairs	4347	Friedel pairs merged	1
Inconsistent equivalents	0	R_{int}	0.0618
R_{sigma}	0.0262	Intensity transformed	0
Omitted reflections	0	Omitted by user (OMIT hkl)	0
Multiplicity	(3023, 2302, 2114, 1592, 1098, 594, 338, 179, 86, 19, 2)	Maximum multiplicity	23
Removed systematic absences	1317	Filtered off (Shel/OMIT)	0

Table 1: Fractional Atomic Coordinates (×10^4) and Equivalent Isotropic Displacement Parameters (Å^2×10^3) for **5**. U_{eq} is defined as 1/3 of the trace of the orthogonalised U_{ij}.

Atom	x	y	z	U_{eq}
P1	7746.0(5)	6329.1(6)	6064.0(2)	21.02(17)
O1	8506.5(15)	5674.2(18)	4938.2(7)	27.8(4)
N1	7717.3(16)	9396(2)	5949.2(8)	24.8(4)
N2	5717.6(17)	8015(2)	5492.1(9)	24.9(4)
N3	6490.7(17)	8308(2)	6685.1(9)	24.5(4)
N4	8129.0(18)	1750(2)	4495.9(9)	27.5(4)
C1	6260(2)	6481(3)	5433.7(10)	24.3(4)
C2	6580(2)	9266(3)	5407.0(10)	26.4(5)
C3	5386(2)	8212(3)	6124.9(10)	25.6(5)
C4	7317(2)	9558(3)	6564.7(10)	26.0(5)
C5	7179(2)	6831(2)	6792.7(10)	24.2(4)
C6	8589(2)	8111(2)	5962.9(11)	25.1(5)
C7	8598.2(19)	4538(2)	6191.2(10)	22.5(4)
C8	8972(2)	4017(3)	6835.1(10)	24.2(4)
C9	9691(2)	2692(3)	6987.8(10)	25.5(5)
C10	10022(2)	1844(3)	6499.1(11)	26.6(5)
C11	9616(2)	2310(3)	5850.0(10)	25.7(5)
C12	8917(2)	3642(2)	5687.9(10)	23.5(4)
C13	8596.7(19)	4269(3)	5010.7(10)	23.6(5)
C14	8363(2)	3253(3)	4419.7(10)	23.8(5)
C15	8320(2)	3936(3)	3818.1(10)	27.1(5)
C16	8004(2)	3027(3)	3261.1(11)	30.5(5)
C17	7736(2)	1493(3)	3328.2(11)	30.7(5)
C18	7818(2)	895(3)	3949.1(11)	29.4(5)
P2	5469.9(5)	6916.6(6)	3293.5(2)	23.96(18)
F1	6941.4(12)	7125.4(17)	3317.1(7)	35.1(3)
F2	5772.6(13)	5460.4(16)	3768.6(7)	35.2(3)
F3	5605.7(15)	8022.7(17)	3909.9(7)	39.4(4)
F4	5156.7(14)	8381.3(18)	2819.5(7)	40.4(4)
F5	3990.2(13)	6718.3(17)	3257.1(7)	36.8(3)
F6	5329.8(15)	5801(2)	2675.7(7)	45.1(4)

Table 2: Anisotropic Displacement Parameters (×10[4]) **5**. The anisotropic displacement factor exponent takes the form: $-2\pi^2[h^2a^{*2} \times U_{11} + ... +2hka^* \times b^* \times U_{12}]$

Atom	U_{11}	U_{22}	U_{33}	U_{23}	U_{13}	U_{12}
P1	25.9(3)	22.6(3)	13.6(3)	-0.74(18)	2.9(2)	0.7(2)
O1	38.4(9)	25.8(8)	19.6(7)	-0.3(6)	7.5(6)	2.7(7)
N1	27.8(9)	28.5(10)	17.6(8)	-2.5(7)	4.6(7)	-0.2(7)
N2	28.2(9)	26.9(10)	17.9(9)	-0.1(7)	1.9(7)	2.6(7)
N3	31.3(9)	23.9(9)	18.1(8)	-0.5(7)	5.1(7)	2.3(7)
N4	31.4(10)	31.2(10)	20.4(9)	-4.5(7)	7.2(7)	-2.0(8)
C1	27.2(10)	26.8(11)	17.5(10)	-1.9(8)	2.4(8)	-1.5(9)
C2	31.7(11)	28.2(12)	17.7(10)	3.3(8)	2.6(8)	1.1(9)
C3	27.0(11)	28.0(11)	22.0(11)	0.6(8)	5.9(9)	2.3(9)
C4	33.1(11)	25.4(11)	18.4(10)	-3.5(8)	4.1(8)	1.1(9)
C5	32.0(11)	23.5(11)	16.6(10)	-0.5(8)	4.8(8)	2.8(9)
C6	27.5(11)	26.0(11)	21.2(10)	-1.0(8)	4.9(8)	-0.7(8)
C7	25.1(10)	23.6(10)	18.4(9)	-0.7(8)	3.9(8)	-2.5(8)
C8	29.9(10)	26.5(11)	15.9(10)	-3.4(8)	4.6(8)	-1.1(9)
C9	28.5(11)	29.6(12)	17.1(10)	1.4(8)	2.6(8)	0.5(9)
C10	28.9(11)	26.5(11)	23.5(11)	1.6(9)	4.6(9)	2.0(9)
C11	29.8(11)	28.1(11)	19.5(10)	-2.5(8)	6.6(8)	0.2(9)
C12	26.8(10)	24.3(11)	18.9(10)	-2.0(8)	4.8(8)	-1.7(8)
C13	25.9(10)	26.5(12)	19.0(10)	-2.0(8)	6.3(8)	0.8(8)
C14	25.1(10)	28.2(11)	17.9(10)	-2.1(8)	4.8(8)	3.2(8)
C15	31.5(11)	29.8(12)	20.0(10)	1.3(9)	6.1(9)	5.0(9)
C16	35.0(12)	39.0(14)	16.0(10)	-0.3(9)	3.1(9)	7.5(10)
C17	31.5(11)	37.5(13)	21.1(11)	-9.0(9)	2.3(9)	4.4(10)
C18	32.5(11)	30.6(12)	25.5(11)	-6.4(9)	7.9(9)	-1.6(9)
P2	28.7(3)	23.3(3)	17.6(3)	-0.7(2)	0.9(2)	0.2(2)
F1	29.8(7)	37.1(8)	37.7(8)	8.1(6)	6.4(6)	-1.0(6)
F2	39.9(7)	29.7(7)	36.7(8)	9.9(6)	10.2(6)	2.2(6)
F3	53.1(9)	35.8(8)	28.2(7)	-10.1(6)	7.1(6)	-1.4(6)
F4	39.5(8)	40.8(8)	38.7(8)	18.0(7)	4.7(6)	5.3(6)
F5	30.2(7)	35.9(8)	43.8(8)	1.1(6)	7.5(6)	-0.6(6)
F6	51.3(9)	54.3(10)	29.0(8)	-18.1(7)	7.9(6)	-1.9(7)

Table 3: Bond Lengths in Å for **5**.

Atom	Atom	Length/Å
P1	C1	1.839(2)
P1	C5	1.839(2)
P1	C6	1.835(2)
P1	C7	1.796(2)
O1	C13	1.229(3)
N1	C2	1.477(3)
N1	C4	1.470(3)
N1	C6	1.459(3)
N2	C1	1.472(3)
N2	C2	1.472(3)
N2	C3	1.473(3)
N3	C3	1.475(3)
N3	C4	1.468(3)
N3	C5	1.473(3)
N4	C14	1.345(3)
N4	C18	1.345(3)
C7	C8	1.397(3)
C7	C12	1.423(3)
C8	C9	1.384(3)
C9	C10	1.381(3)
C10	C11	1.395(3)
C11	C12	1.380(3)
C12	C13	1.490(3)
C13	C14	1.498(3)
C14	C15	1.391(3)
C15	C16	1.388(3)
C16	C17	1.375(4)
C17	C18	1.392(3)
P2	F1	1.6002(14)
P2	F2	1.5967(14)
P2	F3	1.5939(14)
P2	F4	1.6021(14)
P2	F5	1.6027(14)
P2	F6	1.6008(14)

Table 4: Bond Angles in ° for **5**.

Atom	Atom	Atom	Angle/°
C5	P1	C1	100.11(10)
C6	P1	C1	103.53(10)
C6	P1	C5	100.00(10)
C7	P1	C1	120.29(10)
C7	P1	C5	110.07(10)
C7	P1	C6	119.42(10)
C4	N1	C2	108.78(16)
C6	N1	C2	112.23(17)
C6	N1	C4	112.47(17)
C1	N2	C2	112.07(17)
C1	N2	C3	111.70(17)
C2	N2	C3	108.96(17)
C4	N3	C3	108.62(17)
C4	N3	C5	111.02(17)
C5	N3	C3	111.28(17)
C14	N4	C18	116.6(2)
N2	C1	P1	107.78(14)
N2	C2	N1	113.80(17)
N2	C3	N3	113.83(17)
N3	C4	N1	113.80(17)
N3	C5	P1	109.61(14)
N1	C6	P1	107.77(14)
C8	C7	P1	116.48(16)
C8	C7	C12	118.9(2)
C12	C7	P1	124.66(16)
C9	C8	C7	120.85(19)
C10	C9	C8	120.1(2)
C9	C10	C11	120.0(2)
C12	C11	C10	120.9(2)
C7	C12	C13	118.23(19)
C11	C12	C7	119.27(19)
C11	C12	C13	122.18(19)
O1	C13	C12	118.34(19)
O1	C13	C14	119.10(19)
C12	C13	C14	122.56(19)
N4	C14	C13	118.03(19)
N4	C14	C15	123.7(2)
C15	C14	C13	118.1(2)
C16	C15	C14	118.6(2)
C17	C16	C15	118.5(2)
C16	C17	C18	119.3(2)
N4	C18	C17	123.2(2)
F1	P2	F4	89.61(8)
F1	P2	F5	179.01(8)
F1	P2	F6	89.75(8)
F2	P2	F1	90.78(7)
F2	P2	F4	179.53(9)
F2	P2	F5	90.06(8)
F2	P2	F6	89.92(9)
F3	P2	F1	90.43(8)
F3	P2	F2	89.90(8)
F3	P2	F4	89.83(9)
F3	P2	F5	90.10(8)
F3	P2	F6	179.75(10)
F4	P2	F5	89.55(8)
F6	P2	F4	90.34(9)
F6	P2	F5	89.72(8)

Table 5: Torsion Angles in ° for **5**.

Atom	Atom	Atom	Atom	Angle/°
P1	C7	C8	C9	-176.57(16)
P1	C7	C12	C11	177.63(16)
P1	C7	C12	C13	4.0(3)
O1	C13	C14	N4	-162.4(2)
O1	C13	C14	C15	13.5(3)
N4	C14	C15	C16	0.8(3)
C1	P1	C5	N3	-52.84(17)
C1	P1	C6	N1	49.88(16)
C1	P1	C7	C8	-130.78(17)
C1	P1	C7	C12	49.7(2)
C1	N2	C2	N1	-69.5(2)
C1	N2	C3	N3	69.4(2)
C2	N1	C4	N3	55.7(2)
C2	N1	C6	P1	-60.03(19)
C2	N2	C1	P1	59.28(19)
C2	N2	C3	N3	-54.9(2)
C3	N2	C1	P1	-63.32(19)
C3	N2	C2	N1	54.6(2)
C3	N3	C4	N1	-55.9(2)
C3	N3	C5	P1	60.4(2)
C4	N1	C2	N2	-54.9(2)
C4	N1	C6	P1	63.01(19)
C4	N3	C3	N2	55.5(2)
C4	N3	C5	P1	-60.68(19)
C5	P1	C1	N2	53.50(16)
C5	P1	C6	N1	-53.17(16)
C5	P1	C7	C8	-15.3(2)
C5	P1	C7	C12	165.15(18)
C5	N3	C3	N2	-67.0(2)
C5	N3	C4	N1	66.7(2)
C6	P1	C1	N2	-49.46(16)
C6	P1	C5	N3	52.99(16)
C6	P1	C7	C8	99.40(18)
C6	P1	C7	C12	-80.1(2)
C6	N1	C2	N2	70.2(2)
C6	N1	C4	N3	-69.2(2)
C7	P1	C1	N2	174.01(13)
C7	P1	C5	N3	179.53(14)
C7	P1	C6	N1	-173.13(13)
C7	C8	C9	C10	-1.6(3)
C7	C12	C13	O1	25.6(3)
C7	C12	C13	C14	-153.4(2)
C8	C7	C12	C11	-1.9(3)
C8	C7	C12	C13	-175.49(19)
C8	C9	C10	C11	-1.0(3)
C9	C10	C11	C12	2.1(3)
C10	C11	C12	C7	-0.6(3)
C10	C11	C12	C13	172.7(2)
C11	C12	C13	O1	-147.8(2)
C11	C12	C13	C14	33.2(3)
C12	C7	C8	C9	3.0(3)
C12	C13	C14	N4	16.6(3)
C12	C13	C14	C15	-167.47(19)

Atom	Atom	Atom	Atom	Angle/°
C13	C14	C15	C16	- 174.91(19)
C14	N4	C18	C17	0.2(3)
C14	C15	C16	C17	0.5(3)
C15	C16	C17	C18	-1.4(3)
C16	C17	C18	N4	1.1(3)
C18	N4	C14	C13	174.57(19)
C18	N4	C14	C15	-1.1(3)

Table 6: Hydrogen Fractional Atomic Coordinates (×10[4]) and Equivalent Isotropic Displacement Parameters (Å^2×10^3) for **5.** U_{eq} is defined as 1/3 of the trace of the orthogonalised U_{ij}.

Atom	x	y	z	U_{eq}
H1A	6422	6355	5004	29
H1B	5674	5682	5496	29
H2A	6123	10235	5364	32
H2B	6844	9093	5004	32
H3A	4861	7351	6192	31
H3B	4888	9146	6110	31
H4A	8064	9593	6923	31
H4B	6875	10532	6559	31
H5A	6623	6026	6881	29
H5B	7891	6916	7168	29
H6A	9325	8234	6323	30
H6B	8869	8076	5559	30
H8	8735	4568	7165	29
H9	9951	2372	7420	31
H10	10516	962	6603	32
H11	9819	1716	5522	31
H15	8499	4979	3789	33
H16	7973	3446	2852	37
H17	7504	863	2963	37
H18	7649	-148	3988	35

Sample ID: **compound 6**

Crystal Data and Experimental

Crystal Data. $C_{18}H_{22}F_6N_4P_2$, M_r = 470.33, monoclinic, $P2_1/n$ (No. 14), a = 8.69330(10) Å, b = 11.10590(10) Å, c = 20.6765(3) Å, β = 99.9350(10)°, α = γ = 90°, V = 1966.32(4) Å^3, T = 100.00(10) K, Z = 4, Z' = 1, μ(MoK_α) = 0.291 mm^{-1}, 44146 reflections measured, 4500 unique (R_{int} = 0.0149) which were used in all calculations. The final wR_2 was 0.0975 (all data) and R_1 was 0.0361 (I > 2(I)).

**Compound**	**6**
Formula	$C_{18}H_{22}F_6N_4P_2$
$D_{calc.}$/ g cm^{-3}	1.589
μ/mm^{-1}	0.291
Formula Weight	470.33
Colour	colourless
Shape	block
Size/mm^3	0.360×0.280×0.120
T/K	100.00(10)
Crystal System	monoclinic
Space Group	$P2_1/n$
a/Å	8.69330(10)
b/Å	11.10590(10)
c/Å	20.6765(3)
α/°	90
β/°	99.9350(10)
γ/°	90
V/Å^3	1966.32(4)
Z	4
Z'	1
Wavelength/Å	0.71075
Radiation type	MoK_α
Θ_{min}/°	2.089
Θ_{max}/°	27.486
Measured Refl.	44146
Independent Refl.	4500
Reflections with I > 2(I)	4349
R_{int}	0.0149
Parameters	271
Restraints	0
Largest Peak	0.856
Deepest Hole	-0.461
GooF	1.042
wR_2 (all data)	0.0975
wR_2	0.0968
R_1 (all data)	0.0371
R_1	0.0361

Structure Quality Indicators

**Reflections:**	d min (Mo) 0.77	I/σ 145.1	Rint 1.49%	complete 100% (IUCr) 100%
**Refinement:**	Shift -0.001	Max Peak 0.9	Min Peak -0.5	GooF 1.042

A colourless block-shaped crystal with dimensions 0.360×0.280×0.120 mm^3 was mounted on a MITIGEN holder in perfluoroether oil. Data were collected using a Rigaku FRE+ diffractometer equipped with VHF Varimax confocal mirrors and an AFC12 goniometer and HyPix 6000HE detector, and equipped with an Oxford Cryosystems low-temperature device operating at T = 100.00(10) K.

Data were measured using ω scans using MoK_α radiation. The total number of runs and images was based on the strategy calculation from the program **CrysAlisPro** (Rigaku, V1.171.40.39a, 2019). The maximum resolution that was achieved was Θ = 27.486° (0.77 Å).

The diffraction pattern was indexed and the unit cell was refined using **CrysAlisPro** (Rigaku, V1.171.40.39a, 2019) on 32715 reflections, 74% of the observed reflections.

Data reduction, scaling and absorption corrections were performed using **CrysAlisPro** (Rigaku, V1.171.40.39a, 2019). The final completeness is 99.60 % out to 27.486° in Θ.

A multi-scan absorption correction was performed using CrysAlisPro 1.171.40.39a (Rigaku Oxford Diffraction, 2019) using spherical harmonics as implemented in SCALE3 ABSPACK. The absorption coefficient μ of this material is 0.291 mm^{-1} at this wavelength (λ = 0.71075 Å) and the minimum and maximum transmissions are 0.777 and 1.000.

The structure was solved and the space group $P2_1/n$ (# 14) determined by the **ShelXT** (Sheldrick, 2015) structure solution program using Intrinsic Phasing and refined by Least Squares using version 2014/7 of **ShelXL** (Sheldrick, 2015). All non-hydrogen atoms were refined anisotropically. Hydrogen atom positions were calculated geometrically and refined using the riding model.

There is a single molecule in the asymmetric unit, which is represented by the reported sum formula. In other words: Z is 4 and Z' is 1.

Citations

CrysAlisPro Software System, Rigaku Oxford Diffraction, (2019).

O.V. Dolomanov and L.J. Bourhis and R.J. Gildea and J.A.K. Howard and H. Puschmann, Olex2: A complete structure solution, refinement and analysis program, *J. Appl. Cryst.*, (2009), **42**, 339-341.

Sheldrick, G.M., Crystal structure refinement with ShelXL, *Acta Cryst.*, (2015), **C27**, 3-8.

Sheldrick, G.M., ShelXT-Integrated space-group and crystal-structure determination, *Acta Cryst.*, (2015), **A71**, 3-8.

Reflection Statistics

Total reflections (after filtering)	45374	Unique reflections	4500
Completeness	0.997	Mean I/σ	95.98
hkl_{max} collected	(11, 13, 26)	hkl_{min} collected	(-11, -14, -26)
hkl_{max} used	(11, 14, 26)	hkl_{min} used	(-11, 0, 0)
Lim d_{max} collected	100.0	Lim d_{min} collected	0.36
d_{max} used	10.18	d_{min} used	0.77
Friedel pairs	8402	Friedel pairs merged	1
Inconsistent equivalents	3	R_{int}	0.0149
R_{sigma}	0.0069	Intensity transformed	0
Omitted reflections	0	Omitted by user (OMIT hkl)	2
Multiplicity	(3631, 5378, 4391, 2631, 1036, 275, 44, 19)	Maximum multiplicity	21
Removed systematic absences	1226	Filtered off (Shel/OMIT)	0

Table 7: Fractional Atomic Coordinates (×10⁴) and Equivalent Isotropic Displacement Parameters (Å²×10³) for **6**. U_{eq} is defined as 1/3 of the trace of the orthogonalised U_{ij}.

Atom	x	y	z	U_{eq}
P1	2141.2(4)	7609.6(3)	3504.6(2)	12.41(10)
N1	709.2(15)	9398.7(12)	2756.3(6)	19.4(3)
N2	-929.9(15)	8020.1(13)	3286.3(6)	19.5(3)
N3	202.7(16)	7326.6(13)	2340.3(6)	21.5(3)
N4	2654.4(17)	9178.4(12)	4748.9(6)	20.7(3)
C1	2201.9(17)	9153.2(13)	3191.7(7)	16.2(3)
C2	-619.0(18)	9271.5(15)	3107.9(8)	21.8(3)
C3	276.6(16)	7558.3(13)	3810.8(7)	16.1(3)
C4	462.0(19)	8595.4(16)	2182.2(7)	22.9(3)
C5	-1121.2(18)	7264.7(16)	2695.1(8)	24.0(3)
C6	1608.8(18)	6788.3(14)	2725.8(7)	18.3(3)
C7	3916.3(16)	6985.7(12)	3949.6(7)	13.8(3)
C8	5005.4(17)	6653.4(13)	3553.6(7)	17.8(3)
C9	6409.9(17)	6126.0(14)	3830.0(8)	20.8(3)
C10	6761.2(18)	5952.8(13)	4502.8(9)	22.2(3)
C11	5696.1(18)	6294.7(13)	4895.5(8)	19.8(3)
C12	4251.3(17)	6798.6(12)	4632.5(7)	15.2(3)
C13	3148.8(18)	7115.8(13)	5096.4(7)	17.0(3)
C14	3150.9(17)	8443.9(13)	5254.0(7)	15.6(3)
C15	3629.0(19)	8858.8(15)	5889.8(7)	21.1(3)
C16	3527(2)	10080.7(16)	6012.1(8)	25.4(3)
C17	2963(2)	10841.8(15)	5500.2(9)	26.4(4)
C18	2566(2)	10359.0(15)	4878.1(8)	26.3(3)
P2	10306.6(4)	3359.3(4)	3637.2(2)	17.78(11)
F1	8688.6(15)	4050.3(13)	3563.5(7)	49.9(4)
F2	10362.4(14)	3632.6(12)	2879.6(5)	38.7(3)
F3	9400.2(15)	2138.6(11)	3427.6(7)	45.9(3)
F4	10274.8(15)	3066.6(13)	4388.2(5)	41.8(3)
F5	11254.5(17)	4571.6(11)	3834.7(7)	51.7(4)
F6	11932.8(13)	2650.5(11)	3700.9(6)	37.2(3)

Table 8: Anisotropic Displacement Parameters (×10[4]) **6.** The anisotropic displacement factor exponent takes the form: $-2\pi^2[h^2a^{*2} \times U_{11} + ... + 2hka^* \times b^* \times U_{12}]$

Atom	U_{11}	U_{22}	U_{33}	U_{23}	U_{13}	U_{12}
P1	12.71(17)	13.42(17)	11.09(17)	-0.23(12)	2.02(13)	0.74(12)
N1	19.7(6)	23.1(6)	14.8(6)	4.5(5)	1.6(5)	5.2(5)
N2	14.1(6)	25.8(7)	18.2(6)	0.9(5)	1.3(5)	2.1(5)
N3	18.4(6)	30.3(7)	14.6(6)	-3.2(5)	-0.4(5)	-1.0(5)
N4	32.4(7)	13.9(6)	14.9(6)	1.3(5)	1.2(5)	-2.0(5)
C1	18.5(7)	16.2(7)	13.7(6)	2.9(5)	2.4(5)	0.4(5)
C2	20.2(7)	24.8(8)	20.3(7)	4.1(6)	3.8(6)	7.7(6)
C3	13.7(6)	19.5(7)	15.7(6)	0.6(5)	4.3(5)	0.5(5)
C4	23.5(7)	31.6(8)	12.4(6)	3.6(6)	-0.2(6)	3.6(6)
C5	16.8(7)	34.1(9)	19.8(7)	-3.4(6)	-0.6(6)	-3.3(6)
C6	19.8(7)	20.3(7)	14.5(6)	-4.9(5)	2.2(5)	-0.2(6)
C7	13.5(6)	10.6(6)	16.8(6)	-0.5(5)	1.1(5)	0.1(5)
C8	17.6(7)	16.1(7)	20.1(7)	-3.4(5)	4.3(5)	-0.6(5)
C9	15.3(7)	14.0(7)	34.0(8)	-3.9(6)	6.8(6)	-0.4(5)
C10	14.9(6)	12.0(6)	37.7(9)	3.6(6)	-0.8(6)	0.3(5)
C11	20.3(7)	14.1(7)	22.8(7)	4.8(5)	-2.5(6)	-1.7(5)
C12	18.0(7)	9.4(6)	17.8(7)	1.1(5)	1.8(5)	-1.9(5)
C13	24.1(7)	13.7(6)	13.5(6)	2.6(5)	4.0(5)	-0.7(5)
C14	17.9(7)	15.9(7)	13.6(6)	1.0(5)	4.1(5)	-2.0(5)
C15	22.4(7)	27.9(8)	13.1(7)	-0.5(6)	3.6(5)	-1.2(6)
C16	25.6(8)	32.2(9)	20.0(7)	-11.6(6)	8.3(6)	-9.5(7)
C17	32.7(9)	16.9(7)	32.9(9)	-8.4(6)	14.7(7)	-7.8(6)
C18	40.0(9)	14.3(7)	25.0(8)	2.8(6)	6.7(7)	-0.9(6)
P2	15.70(19)	20.5(2)	17.10(19)	0.52(14)	2.66(14)	1.21(14)
F1	35.3(6)	62.9(9)	51.2(7)	0.1(6)	6.9(6)	30.6(6)
F2	38.9(6)	56.2(8)	20.6(5)	9.9(5)	3.9(4)	1.8(5)
F3	45.4(7)	31.1(6)	53.8(8)	5.5(6)	-11.9(6)	-14.5(5)
F4	40.2(6)	63.5(8)	23.5(5)	7.7(5)	10.6(5)	4.6(6)
F5	64.6(9)	24.8(6)	55.0(8)	3.0(5)	-19.9(7)	-13.9(6)
F6	25.2(5)	47.5(7)	39.5(6)	10.8(5)	7.1(5)	14.4(5)

Table 9: Bond Lengths in Å for **6.**

Atom	Atom	Length/Å
P1	C1	1.8362(15)
P1	C3	1.8400(15)
P1	C6	1.8391(15)
P1	C7	1.7940(14)
N1	C1	1.4716(18)
N1	C2	1.473(2)
N1	C4	1.471(2)
N2	C2	1.475(2)
N2	C3	1.4653(19)
N2	C5	1.468(2)
N3	C4	1.473(2)
N3	C5	1.469(2)
N3	C6	1.467(2)
N4	C14	1.3368(19)
N4	C18	1.343(2)
C7	C8	1.404(2)
C7	C12	1.4070(19)

Atom	Atom	Length/Å
C8	C9	1.386(2)
C9	C10	1.385(2)
C10	C11	1.386(2)
C11	C12	1.397(2)
C12	C13	1.510(2)
C13	C14	1.511(2)
C14	C15	1.387(2)
C15	C16	1.386(2)
C16	C17	1.377(3)
C17	C18	1.381(2)
P2	F1	1.5864(12)
P2	F2	1.6046(11)
P2	F3	1.5902(12)
P2	F4	1.5912(11)
P2	F5	1.5944(12)
P2	F6	1.6035(11)

Table 10: Bond Angles in ° for **6.**

Atom	Atom	Atom	Angle/°
C1	P1	C3	103.34(7)
C1	P1	C6	99.95(7)
C6	P1	C3	100.51(7)
C7	P1	C1	117.83(7)
C7	P1	C3	122.85(7)
C7	P1	C6	108.77(7)
C1	N1	C2	111.34(11)
C4	N1	C1	111.84(12)
C4	N1	C2	108.80(13)
C3	N2	C2	112.10(12)
C3	N2	C5	112.34(12)
C5	N2	C2	109.24(13)
C5	N3	C4	108.58(13)
C6	N3	C4	111.44(12)
C6	N3	C5	111.15(12)
C14	N4	C18	117.53(14)
N1	C1	P1	108.21(10)
N1	C2	N2	114.00(12)
N2	C3	P1	107.16(10)
N1	C4	N3	114.07(12)
N2	C5	N3	113.76(13)
N3	C6	P1	109.51(10)
C8	C7	P1	114.16(11)
C8	C7	C12	120.19(13)
C12	C7	P1	125.63(11)
C9	C8	C7	120.37(14)
C10	C9	C8	119.92(14)
C9	C10	C11	119.78(14)
C10	C11	C12	121.91(14)
C7	C12	C13	123.97(13)
C11	C12	C7	117.80(14)
C11	C12	C13	118.22(13)
C12	C13	C14	112.81(12)
N4	C14	C13	116.04(13)
N4	C14	C15	122.77(14)
C15	C14	C13	121.18(13)
C16	C15	C14	118.68(15)
C17	C16	C15	119.10(15)
C16	C17	C18	118.44(15)
N4	C18	C17	123.40(16)
F1	P2	F2	89.45(7)
F1	P2	F3	89.82(8)
F1	P2	F4	91.62(7)
F1	P2	F5	91.53(8)
F1	P2	F6	179.10(8)
F3	P2	F2	89.55(7)
F3	P2	F4	90.04(7)
F3	P2	F5	178.27(9)
F3	P2	F6	89.55(7)
F4	P2	F2	178.86(7)
F4	P2	F5	91.02(8)
F4	P2	F6	89.03(7)
F5	P2	F2	89.37(7)
F5	P2	F6	89.09(8)
F6	P2	F2	89.90(6)

Table 11: Torsion Angles in ° for **6**.

Atom	Atom	Atom	Atom	Angle/°
P1	C7	C8	C9	-178.08(11)
P1	C7	C12	C11	179.64(11)
P1	C7	C12	C13	-0.7(2)
N4	C14	C15	C16	2.8(2)
C1	P1	C3	N2	50.19(11)
C1	P1	C6	N3	-53.07(12)
C1	P1	C7	C8	-74.58(12)
C1	P1	C7	C12	106.65(13)
C1	N1	C2	N2	-69.70(17)
C1	N1	C4	N3	68.39(17)
C2	N1	C1	P1	59.45(14)
C2	N1	C4	N3	-55.01(16)
C2	N2	C3	P1	-60.45(13)
C2	N2	C5	N3	55.06(17)
C3	P1	C1	N1	-50.16(11)
C3	P1	C6	N3	52.64(12)
C3	P1	C7	C8	154.77(10)
C3	P1	C7	C12	-24.00(16)
C3	N2	C2	N1	70.96(16)
C3	N2	C5	N3	-70.00(17)
C4	N1	C1	P1	-62.50(14)
C4	N1	C2	N2	54.00(16)
C4	N3	C5	N2	-55.60(17)
C4	N3	C6	P1	60.85(14)
C5	N2	C2	N1	-54.23(16)
C5	N2	C3	P1	63.02(14)
C5	N3	C4	N1	55.79(16)
C5	N3	C6	P1	-60.42(15)
C6	P1	C1	N1	53.25(11)
C6	P1	C3	N2	-52.78(11)
C6	P1	C7	C8	38.11(13)
C6	P1	C7	C12	-140.66(12)
C6	N3	C4	N1	-66.97(16)
C6	N3	C5	N2	67.33(18)
C7	P1	C1	N1	170.76(9)
C7	P1	C3	N2	-173.40(9)
C7	P1	C6	N3	-177.14(10)
C7	C8	C9	C10	-1.7(2)
C7	C12	C13	C14	-78.53(18)
C8	C7	C12	C11	0.9(2)
C8	C7	C12	C13	-179.42(13)
C8	C9	C10	C11	0.8(2)
C9	C10	C11	C12	1.0(2)
C10	C11	C12	C7	-1.8(2)
C10	C11	C12	C13	178.53(14)
C11	C12	C13	C14	101.11(15)
C12	C7	C8	C9	0.8(2)
C12	C13	C14	N4	63.52(18)
C12	C13	C14	C15	-117.12(15)
C13	C14	C15	C16	-176.56(14)
C14	N4	C18	C17	0.2(3)
C14	C15	C16	C17	-0.3(2)

Atom	Atom	Atom	Atom	Angle/°
C15	C16	C17	C18	-2.0(2)
C16	C17	C18	N4	2.1(3)
C18	N4	C14	C13	176.67(14)
C18	N4	C14	C15	-2.7(2)

Table 12: Hydrogen Fractional Atomic Coordinates (×10^4) and Equivalent Isotropic Displacement Parameters ($Å^2$×10^3) for **6.** U_{eq} is defined as 1/3 of the trace of the orthogonalised U_{ij}.

Atom	x	y	z	U_{eq}
H1A	2361	9732	3561	19
H1B	3077	9240	2946	19
H2A	-1567	9598	2829	26
H2B	-412	9762	3513	26
H3A	32	6721	3923	19
H3B	336	8062	4210	19
H4A	1385	8644	1962	27
H4B	-452	8886	1868	27
H5A	-2081	7516	2395	29
H5B	-1264	6418	2823	29
H6A	2479	6827	2475	22
H6B	1411	5932	2816	22
H8	4778	6791	3093	21
H9	7131	5883	3558	25
H10	7729	5601	4694	27
H11	5956	6183	5357	24
H13A	3449	6657	5509	20
H13B	2077	6869	4897	20
H15	4018	8316	6235	25
H16	3842	10389	6443	30
H17	2851	11679	5573	32
H18	2211	10890	4523	32

Sample ID: **compound 7**

Crystal Data and Experimental

Crystal Data. $C_{17}H_{21}F_6N_5P_2$, M_r = 471.33, monoclinic, $P2_1/c$ (No. 14), a = 8.5789(2) Å, b = 12.0441(2) Å, c = 19.9253(4) Å, β = 94.912(2)°, α = γ = 90°, V = 2051.22(7) Å^3, T = 100.00(10) K, Z = 4, Z' = 1, μ(MoK$_\alpha$) = 0.280 mm^{-1}, 45940 reflections measured, 4689 unique (R_{int} = 0.0317) which were used in all calculations. The final wR_2 was 0.0880 (all data) and R_1 was 0.0331 (I > 2(I)).

**Compound**	**7**
Formula	$C_{17}H_{21}F_6N_5P_2$
$D_{calc.}$/ g cm^{-3}	1.526
μ/mm^{-1}	0.280
Formula Weight	471.33
Colour	colourless
Shape	block
Size/mm^3	0.320×0.240×0.150
T/K	100.00(10)
Crystal System	monoclinic
Space Group	$P2_1/c$
a/Å	8.5789(2)
b/Å	12.0441(2)
c/Å	19.9253(4)
α/°	90
β/°	94.912(2)
γ/°	90
V/Å^3	2051.22(7)
Z	4
Z'	1
Wavelength/Å	0.71075
Radiation type	MoK$_\alpha$
Θ_{min}/°	1.978
Θ_{max}/°	27.478
Measured Refl.	45940
Independent Refl.	4689
Reflections with I > 2(I)	4395
R_{int}	0.0317
Parameters	312
Restraints	0
Largest Peak	0.467
Deepest Hole	-0.329
GooF	1.042
wR_2 (all data)	0.0880
wR_2	0.0866
R_1 (all data)	0.0353
R_1	0.0331

Structure Quality Indicators

**Reflections:**	d min (Mo) 0.77	I/σ 75.1	Rint 3.17%	complete 100% (IUCr) 100%
**Refinement:**	Shift -0.001	Max Peak 0.5	Min Peak -0.3	GooF 1.042

A colourless block-shaped crystal with dimensions 0.320×0.240×0.150 mm^3 was mounted on a MITIGEN holder in perfluoroether oil. Data were collected using a Rigaku FRE+ diffractometer equipped with VHF Varimax confocal mirrors and an AFC12 goniometer and HyPix 6000HE detector, and equipped with an Oxford Cryosystems low-temperature device operating at T = 100.00(10) K.

Data were measured using ω scans of 0.5 ° per frame for 1 s using MoK_α radiation. The total number of runs and images was based on the strategy calculation from the program **CrysAlisPro** (Rigaku, V1.171.40.39a, 2019) The maximum resolution that was achieved was Θ = 27.478° (0.77 Å).

The diffraction pattern was indexed and the unit cell was refined using **CrysAlisPro** (Rigaku, V1.171.40.39a, 2019) on 23428 reflections, 51% of the observed reflections.

Data reduction, scaling and absorption corrections were performed using **CrysAlisPro** (Rigaku, V1.171.40.39a, 2019). The final completeness is 99.80 % out to 27.478° in Θ.

A multi-scan absorption correction was performed using CrysAlisPro 1.171.40.39a (Rigaku Oxford Diffraction, 2019) using spherical harmonics as implemented in SCALE3 ABSPACK. The absorption coefficient μ of this material is 0.280 mm^{-1} at this wavelength (λ = 0.71075 Å) and the minimum and maximum transmissions are 0.673 and 1.000.

The structure was solved and the space group $P2_1/c$ (# 14) determined by the **ShelXT** (Sheldrick, 2015) structure solution program using Intrinsic Phasing and refined by Least Squares using version 2014/7 of **ShelXL** (Sheldrick, 2015). All non-hydrogen atoms were refined anisotropically. Hydrogen atom positions were calculated geometrically and refined using the riding model. The PF_6 was disordered.

There is a single molecule in the asymmetric unit, which is represented by the reported sum formula. In other words: Z is 4 and Z' is 1.

Citations

CrysAlisPro Software System, Rigaku Oxford Diffraction, (2019).

O.V. Dolomanov and L.J. Bourhis and R.J. Gildea and J.A.K. Howard and H. Puschmann, Olex2: A complete structure solution, refinement and analysis program, *J. Appl. Cryst.*, (2009), **42**, 339-341.

Sheldrick, G.M., Crystal structure refinement with ShelXL, *Acta Cryst.*, (2015), **C27**, 3-8.

Sheldrick, G.M., ShelXT-Integrated space-group and crystal-structure determination, *Acta Cryst.*, (2015), **A71**, 3-8.

Reflection Statistics

Total reflections (after filtering)	47116	Unique reflections	4689
Completeness	0.998	Mean I/σ	49.7
hkl_{max} collected	(11, 15, 25)	hkl_{min} collected	(-10, -15, -25)
hkl_{max} used	(11, 15, 25)	hkl_{min} used	(-11, 0, 0)
Lim d_{max} collected	100.0	Lim d_{min} collected	0.36
d_{max} used	12.04	d_{min} used	0.77
Friedel pairs	8089	Friedel pairs merged	1
Inconsistent equivalents	29	R_{int}	0.0317
R_{sigma}	0.0133	Intensity transformed	0
Omitted reflections	0	Omitted by user (OMIT hkl)	2
Multiplicity	(3250, 4908, 5015, 2771, 1274, 216, 25, 10)	Maximum multiplicity	18
Removed systematic absences	1174	Filtered off (Shel/OMIT)	0

Table 13: Fractional Atomic Coordinates (×10[4]) and Equivalent Isotropic Displacement Parameters (Å^2×10^3) for **7**. U_{eq} is defined as 1/3 of the trace of the orthogonalised U_{ij}.

Atom	x	y	z	U_{eq}
P1	4382.2(4)	2158.3(3)	3748.7(2)	15.54(9)
N1	1450.5(13)	2158.5(9)	4132.2(6)	22.3(2)
N2	3279.0(14)	728.7(10)	4619.8(6)	24.1(2)
N3	2221.6(13)	601.4(9)	3430.9(6)	23.2(2)
N4	6051.9(13)	3938.8(10)	4509.7(6)	19.7(2)
N5	6360.9(13)	4324.4(9)	5641.3(6)	20.4(2)
C1	2639.6(15)	2946.9(10)	3939.7(7)	19.4(2)
C2	4741.4(16)	1314.8(11)	4510.2(6)	20.9(3)
C3	2012.0(17)	1513.2(12)	4733.2(7)	26.5(3)
C4	2764.1(17)	3.9(11)	4049.7(8)	26.0(3)
C5	982.1(16)	1385.0(11)	3582.0(8)	24.8(3)
C6	3522.9(16)	1160.1(11)	3133.5(7)	20.9(3)
C7	5997.1(14)	2910.0(10)	3468.3(6)	17.2(2)
C8	6542.6(16)	2701.3(11)	2837.0(7)	21.5(3)
C9	7765.0(17)	3328.1(12)	2628.8(7)	25.5(3)
C10	8451.9(18)	4146.9(12)	3042.2(8)	28.9(3)
C11	7925.7(17)	4351.1(12)	3669.1(8)	26.9(3)
C12	6689.4(15)	3735.8(11)	3881.3(6)	19.0(2)
C13	6969.6(15)	3848.7(10)	5113.5(7)	19.0(2)
C14	8393.1(17)	3264.4(11)	5174.3(7)	25.1(3)
C15	9188.2(18)	3186.2(12)	5804.4(8)	29.1(3)
C16	8564.2(18)	3669.8(12)	6356.0(7)	27.9(3)
C17	7159.8(18)	4222.1(11)	6249.2(7)	24.8(3)
P2	2398.2(4)	4045.0(3)	1801.6(2)	23.58(10)
F1	3779.5(12)	4043.1(12)	2390.0(5)	55.0(3)
F2	1483(5)	4959(2)	2224(2)	34.4(7)
F2A	1316(12)	4569(18)	2250(5)	70(4)
F3	1499(5)	3105(3)	2209.9(18)	58.1(8)
F3A	1991(14)	2814(8)	1939(9)	75(5)
F4	3214(7)	3127(5)	1418(2)	62.3(16)
F4A	3729(9)	3567(10)	1290(4)	49.1(19)
F5	3170(6)	5018(5)	1439(2)	61.3(13)
F5A	3006(10)	5244(7)	1553(6)	53(2)
F6	1010.6(12)	4059.0(7)	1217.4(5)	36.5(2)

Table 14: Anisotropic Displacement Parameters (×10[4]) **7.** The anisotropic displacement factor exponent takes the form: $-2\pi^2[h^2a^{*2} \times U_{11} + ... + 2hka^* \times b^* \times U_{12}]$

Atom	U_{11}	U_{22}	U_{33}	U_{23}	U_{13}	U_{12}
P1	17.14(16)	15.82(16)	13.83(15)	0.72(11)	2.27(11)	1.33(11)
N1	19.0(5)	19.9(5)	28.8(6)	1.6(4)	6.1(4)	0.9(4)
N2	26.5(6)	21.9(5)	24.9(6)	7.3(5)	8.3(5)	2.7(5)
N3	21.6(5)	18.2(5)	29.9(6)	0.4(5)	2.0(5)	-0.8(4)
N4	19.5(5)	23.3(6)	16.4(5)	-5.0(4)	2.1(4)	3.5(4)
N5	25.4(6)	18.3(5)	17.2(5)	-1.1(4)	1.0(4)	0.4(4)
C1	18.4(6)	16.4(6)	23.5(6)	0.3(5)	3.2(5)	2.6(5)
C2	23.1(6)	22.2(6)	17.5(6)	4.7(5)	3.0(5)	3.7(5)
C3	27.4(7)	26.8(7)	26.8(7)	5.2(5)	12.0(6)	2.2(6)
C4	27.5(7)	17.0(6)	34.4(8)	4.8(5)	7.0(6)	1.1(5)
C5	19.4(6)	20.9(6)	34.0(7)	0.3(5)	1.5(5)	-0.6(5)
C6	23.2(6)	19.1(6)	20.1(6)	-2.0(5)	0.6(5)	-0.2(5)
C7	17.9(6)	16.8(6)	17.1(6)	1.8(4)	2.9(5)	1.7(4)
C8	27.5(7)	20.1(6)	17.5(6)	-1.5(5)	6.0(5)	1.8(5)
C9	31.3(7)	25.6(7)	21.4(6)	1.5(5)	12.1(5)	3.4(6)
C10	27.8(7)	27.1(7)	33.5(8)	1.7(6)	13.5(6)	-4.5(6)
C11	26.1(7)	24.8(7)	30.6(7)	-6.0(6)	6.0(6)	-5.9(5)
C12	19.6(6)	19.9(6)	17.9(6)	-1.7(5)	3.8(5)	2.7(5)
C13	21.8(6)	15.4(5)	19.7(6)	-1.4(5)	1.6(5)	-1.8(5)
C14	26.0(7)	21.5(6)	27.7(7)	-2.0(5)	1.7(5)	4.2(5)
C15	27.9(7)	21.7(7)	36.4(8)	4.9(6)	-5.0(6)	3.6(5)
C16	36.8(8)	20.7(6)	24.2(7)	6.0(5)	-9.0(6)	-3.0(6)
C17	37.1(8)	18.9(6)	17.9(6)	0.4(5)	-0.4(5)	-2.8(5)
P2	22.27(18)	29.8(2)	18.03(17)	3.67(13)	-1.77(13)	1.22(13)
F1	34.6(5)	96.4(10)	30.9(5)	-5.4(6)	-14.1(4)	18.8(6)
F2	37.3(14)	41.8(14)	23.2(11)	-6.2(8)	-2.4(9)	8.3(8)
F2A	30(3)	160(11)	22(2)	-2(5)	17.9(19)	19(5)
F3	77.9(17)	40.5(14)	57.0(15)	26.6(11)	13.0(13)	-5.6(12)
F3A	71(5)	46(4)	100(8)	51(5)	-34(5)	-19(3)
F4	60(2)	84(3)	40.8(14)	-17.9(16)	-5.2(14)	48(2)
F4A	39(3)	71(4)	39(3)	-14(2)	10(2)	26(3)
F5	53.4(18)	91(3)	41.3(13)	22.0(17)	14.2(12)	-37.5(19)
F5A	27(3)	24(2)	104(7)	7(3)	-19(3)	-6.6(18)
F6	40.2(5)	28.2(5)	37.4(5)	-7.0(4)	-18.8(4)	4.2(4)

Table 15: Bond Lengths in Å for **7**.

Atom	Atom	Length/Å
P1	C1	1.8383(13)
P1	C2	1.8301(13)
P1	C6	1.8266(13)
P1	C7	1.7843(13)
N1	C1	1.4685(16)
N1	C3	1.4736(18)
N1	C5	1.4682(18)
N2	C2	1.4720(17)
N2	C3	1.4723(18)
N2	C4	1.4702(19)
N3	C4	1.4685(18)
N3	C5	1.4720(17)
N3	C6	1.4709(17)
N4	C12	1.4295(16)
N4	C13	1.3845(17)
N5	C13	1.3421(17)
N5	C17	1.3453(17)
C7	C8	1.4019(17)
C7	C12	1.3913(18)
C8	C9	1.3842(19)
C9	C10	1.384(2)
C10	C11	1.386(2)
C11	C12	1.3892(19)
C13	C14	1.4057(19)
C14	C15	1.380(2)
C15	C16	1.391(2)
C16	C17	1.377(2)
P2	F1	1.5940(10)
P2	F2	1.628(4)
P2	F2A	1.484(10)
P2	F3	1.626(3)
P2	F3A	1.553(6)
P2	F4	1.545(3)
P2	F4A	1.695(6)
P2	F5	1.555(4)
P2	F5A	1.627(9)
P2	F6	1.5922(9)

Table 16: Bond Angles in ° for **7**.

Atom	Atom	Atom	Angle/°
C2	P1	C1	101.38(6)
C6	P1	C1	100.97(6)
C6	P1	C2	102.75(6)
C7	P1	C1	118.01(6)
C7	P1	C2	117.46(6)
C7	P1	C6	113.79(6)
C1	N1	C3	111.55(11)
C5	N1	C1	111.54(11)
C5	N1	C3	108.77(11)
C2	N2	C3	111.40(11)
C4	N2	C2	111.94(11)
C4	N2	C3	108.83(11)
C4	N3	C5	108.96(11)
C4	N3	C6	111.44(11)
C6	N3	C5	112.06(10)
C13	N4	C12	121.06(11)
C13	N5	C17	117.65(12)
N1	C1	P1	108.39(8)
N2	C2	P1	107.70(9)
N2	C3	N1	114.08(11)
N3	C4	N2	114.23(11)
N1	C5	N3	113.78(11)
N3	C6	P1	107.90(9)
C8	C7	P1	121.18(10)
C12	C7	P1	118.75(10)
C12	C7	C8	120.04(12)
C9	C8	C7	119.38(12)
C10	C9	C8	120.35(13)
C9	C10	C11	120.52(13)
C10	C11	C12	119.72(13)
C7	C12	N4	117.67(11)
C11	C12	N4	122.33(12)
C11	C12	C7	119.97(12)
N4	C13	C14	122.64(12)
N5	C13	N4	114.65(11)
N5	C13	C14	122.66(12)
C15	C14	C13	118.09(13)
C14	C15	C16	119.78(13)
C17	C16	C15	118.11(13)
N5	C17	C16	123.71(13)
F1	P2	F2	88.97(16)
F1	P2	F3	89.17(13)
F1	P2	F4A	86.9(2)
F1	P2	F5A	89.3(3)
F2A	P2	F1	91.2(4)
F2A	P2	F3A	97.9(4)
F2A	P2	F4A	174.3(5)
F2A	P2	F5A	92.2(7)
F2A	P2	F6	88.2(4)
F3	P2	F2	86.66(19)
F3A	P2	F1	91.9(3)
F3A	P2	F4A	87.5(5)
F3A	P2	F5A	169.7(7)
F3A	P2	F6	88.5(2)
F4	P2	F1	91.30(13)
F4	P2	F2	176.9(3)
F4	P2	F3	90.2(2)
F4	P2	F5	94.6(2)
F4	P2	F6	89.34(13)
F5	P2	F1	91.38(19)
F5	P2	F2	88.5(2)
F5	P2	F3	175.12(19)
F5	P2	F6	88.47(18)
F5A	P2	F4A	82.4(4)
F6	P2	F1	179.35(6)
F6	P2	F2	90.40(16)
F6	P2	F3	90.93(12)
F6	P2	F4A	93.7(2)
F6	P2	F5A	90.4(3)

Table 17: Torsion Angles in ° for **7**.

Atom	Atom	Atom	Atom	Angle/°
P1	C7	C8	C9	177.86(10)
P1	C7	C12	N4	-0.41(16)
P1	C7	C12	C11	-178.55(11)
N4	C13	C14	C15	-177.42(13)
N5	C13	C14	C15	-0.2(2)
C1	P1	C2	N2	53.04(10)
C1	P1	C6	N3	-52.84(10)
C1	P1	C7	C8	-119.78(11)
C1	P1	C7	C12	58.52(12)
C1	N1	C3	N2	-68.34(15)
C1	N1	C5	N3	68.13(14)
C2	P1	C1	N1	-52.50(10)
C2	P1	C6	N3	51.64(10)
C2	P1	C7	C8	118.33(11)
C2	P1	C7	C12	-63.38(12)
C2	N2	C3	N1	69.42(15)
C2	N2	C4	N3	-69.22(14)
C3	N1	C1	P1	60.46(12)
C3	N1	C5	N3	-55.31(14)
C3	N2	C2	P1	-62.18(12)
C3	N2	C4	N3	54.35(15)
C4	N2	C2	P1	59.93(12)
C4	N2	C3	N1	-54.47(15)
C4	N3	C5	N1	55.21(15)
C4	N3	C6	P1	-60.67(12)
C5	N1	C1	P1	-61.38(12)
C5	N1	C3	N2	55.10(15)
C5	N3	C4	N2	-54.77(15)
C5	N3	C6	P1	61.73(12)
C6	P1	C1	N1	53.06(10)
C6	P1	C2	N2	-51.11(10)
C6	P1	C7	C8	-1.73(13)
C6	P1	C7	C12	176.56(10)
C6	N3	C4	N2	69.40(14)
C6	N3	C5	N1	-68.59(15)
C7	P1	C1	N1	177.72(8)
C7	P1	C2	N2	-176.82(8)
C7	P1	C6	N3	179.70(8)
C7	C8	C9	C10	0.6(2)
C8	C7	C12	N4	177.91(12)
C8	C7	C12	C11	-0.2(2)
C8	C9	C10	C11	0.0(2)
C9	C10	C11	C12	-0.6(2)
C10	C11	C12	N4	-177.31(13)
C10	C11	C12	C7	0.7(2)
C12	N4	C13	N5	163.86(12)
C12	N4	C13	C14	-18.76(19)
C12	C7	C8	C9	-0.4(2)
C13	N4	C12	C7	121.88(13)
C13	N4	C12	C11	-60.03(18)
C13	N5	C17	C16	0.7(2)
C13	C14	C15	C16	0.6(2)
C14	C15	C16	C17	-0.4(2)
C15	C16	C17	N5	-0.3(2)

Atom	Atom	Atom	Atom	Angle/°
C17	N5	C13	N4	176.97(12)
C17	N5	C13	C14	-0.41(19)

Table 18: Hydrogen Fractional Atomic Coordinates (×10[4]) and Equivalent Isotropic Displacement Parameters (Å[2]×10[3]) for **7**. U_{eq} is defined as 1/3 of the trace of the orthogonalised U_{ij}.

Atom	x	y	z	U_{eq}
H4	5370(20)	4470(15)	4501(9)	27(4)
H1A	2907	3462	4306	23
H1B	2238	3368	3547	23
H2A	5572	785	4455	25
H2B	5054	1785	4894	25
H3A	2380	2024	5088	32
H3B	1140	1102	4889	32
H4A	3626	-476	3955	31
H4B	1922	-465	4180	31
H5A	669	1809	3179	30
H5B	79	966	3700	30
H6A	3143	1539	2722	25
H6B	4302	620	3025	25
H8	6087	2147	2561	26
H9	8127	3198	2209	31
H10	9273	4564	2898	35
H11	8398	4898	3946	32
H14	8788	2940	4801	30
H15	10138	2811	5860	35
H16	9081	3622	6785	33
H17	6740	4542	6618	30

Table 19: Atomic Occupancies for all atoms that are not fully occupied in **7**.

Atom	Occupancy
F2	0.686(15)
F2A	0.314(15)
F3	0.686(15)
F3A	0.314(15)
F4	0.686(15)
F4A	0.314(15)
F5	0.686(15)
F5A	0.314(15)

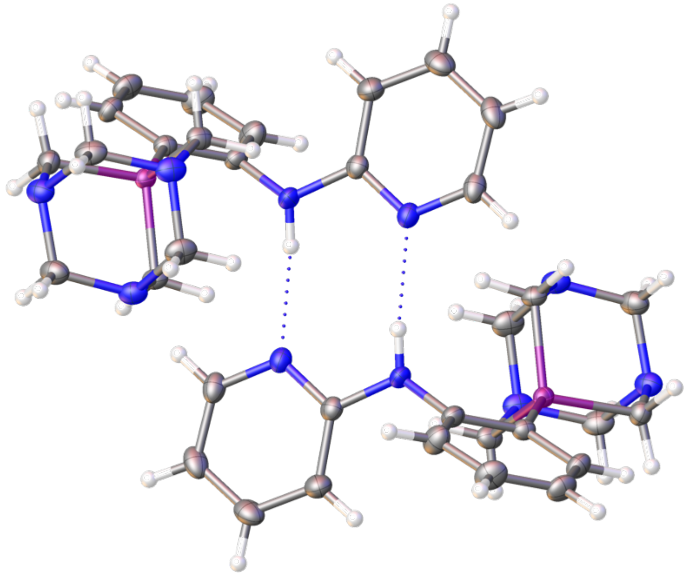

Sample ID: **[Au(C^CO^N)Cl(triphenylphosphine)$_2$]$^+$**

Crystal Data and Experimental

Crystal Data. $C_{52}H_{46}AuClF_6NO_3P_3$, M_r = 1172.22, orthorhombic, $P2_12_12_1$ (No. 19), a = 15.35729(7) Å, b = 16.32785(7) Å, c = 19.12684(9) Å, $\alpha = \beta = \gamma = 90°$, V = 4796.09(4) Å^3, T = 100.01(10) K, Z = 4, Z' = 1, $\mu(CuK_\alpha)$ = 7.803 mm^{-1}, 86713 reflections measured, 8539 unique (R_{int} = 0.0416) which were used in all calculations. The final wR_2 was 0.0495 (all data) and R_1 was 0.0184 (I > 2(I)).

**Compound**	**[Au(C^CO^N)Cl(triphenylphosphine)$_2$]$^+$**
Formula	$C_{52}H_{46}AuClF_6NO_3P_3$
$D_{calc.}$/ g cm^{-3}	1.623
μ/mm^{-1}	7.803
Formula Weight	1172.22
Colour	colourless
Shape	block
Size/mm^3	0.580×0.140×0.060
T/K	100.01(10)
Crystal System	orthorhombic
Flack Parameter	-0.0215(16)
Hooft Parameter	-0.0020(13)
Space Group	$P2_12_12_1$
a/Å	15.35729(7)
b/Å	16.32785(7)
c/Å	19.12684(9)
α/°	90
β/°	90
γ/°	90
V/Å^3	4796.09(4)
Z	4
Z'	1
Wavelength/Å	1.54184
Radiation type	CuK_α
Θ_{min}/°	3.559
Θ_{max}/°	67.052
Measured Refl.	86713
Independent Refl.	8539
Reflections with I > 2(I)	8507
R_{int}	0.0416
Parameters	606
Restraints	0
Largest Peak	0.708
Deepest Hole	-0.784
GooF	1.097
wR_2 (all data)	0.0495
wR_2	0.0494
R_1 (all data)	0.0185
R_1	0.0184

Structure Quality Indicators

**Reflections:**	d min (Cu) 0.84	I/σ 67.0	Rint 4.16%	complete 100% (IUCr) 100%
**Refinement:**	Shift 0.003	Max Peak 0.7	Min Peak -0.8	GooF 1.097	Flack -.022(2)

A colourless block-shaped crystal with dimensions 0.580×0.140×0.060 mm^3 was mounted on a MITIGEN holder in perfluoroether oil. Data were collected using a Rigaku 007HF diffractometer equipped with Varimax confocal mirrors and an AFC11 goniometer and HyPix 6000HE detector, and equipped with an Oxford Cryosystems low-temperature device operating at T = 100.01(10) K.

Data were measured using ω scans of 0.5 ° per frame for 0.5 s using CuK_α radiation. The total number of runs and images was based on the strategy calculation from the program **CrysAlisPro** (Rigaku, V1.171.40.53, 2019). The maximum resolution that was achieved was Θ = 67.052° (0.84 Å).

The diffraction pattern was indexed and the unit cell was refined using **CrysAlisPro** (Rigaku, V1.171.40.53, 2019) on 77083 reflections, 89% of the observed reflections.

Data reduction, scaling and absorption corrections were performed using **CrysAlisPro** (Rigaku, V1.171.40.53, 2019). The final completeness is 99.60 % out to 67.052° in Θ.

A multi-scan absorption correction was performed using CrysAlisPro 1.171.40.53 (Rigaku Oxford Diffraction, 2019) using spherical harmonics as implemented in SCALE3 ABSPACK. The absorption coefficient μ of this material is 7.803 mm^{-1} at this wavelength (λ = 1.54184 Å) and the minimum and maximum transmissions are 0.539 and 1.000.

The structure was solved and the space group $P2_12_12_1$ (# 19) determined by the **ShelXS** (Sheldrick, 2008) structure solution program using Direct Methods and refined by Least Squares using version 2014/7 of **ShelXL** (Sheldrick, 2015). All non-hydrogen atoms were refined anisotropically. Hydrogen atom positions were calculated geometrically and refined using the riding model.

There is a single molecule in the asymmetric unit, which is represented by the reported sum formula. In other words: Z is 4 and Z' is 1.

The Flack parameter was refined to -0.0215(16). Determination of absolute structure using Bayesian statistics on Bijvoet differences using the Olex2 results in -0.0020(13). Note: The Flack parameter is used to determine chirality of the crystal studied, the value should be near 0, a value of 1 means that the stereochemistry is wrong and the model should be inverted. A value of 0.5 means that the crystal consists of a racemic mixture of the two enantiomers.

Reflection Statistics

Total reflections (after filtering)	86907	Unique reflections	8539
Completeness	0.997	Mean I/σ	53.41
hkl_{max} collected	(18, 19, 21)	hkl_{min} collected	(-18, -19, -22)
hkl_{max} used	(18, 19, 22)	hkl_{min} used	(-18, 0, 0)
Lim d_{max} collected	100.0	Lim d_{min} collected	0.77
d_{max} used	16.33	d_{min} used	0.84
Friedel pairs	12058	Friedel pairs merged	0
Inconsistent equivalents	1	R_{int}	0.0416
R_{sigma}	0.0149	Intensity transformed	0
Omitted reflections	0	Omitted by user (OMIT hkl)	49
Multiplicity	(7054, 7675, 5430, 3091, 2376, 1671, 919, 485, 205, 118, 55)	Maximum multiplicity	25
Removed systematic absences	145	Filtered off (Shel/OMIT)	0

Table 20: Fractional Atomic Coordinates (×10^4^) and Equivalent Isotropic Displacement Parameters (Å^2×10^3) for **[Au(C^{CO}N)Cl(triphenylphosphine)$_2$]$^+$**. U_{eq} is defined as 1/3 of the trace of the orthogonalised U_{ij}.

Atom	x	y	z	U_{eq}
Au1	7590.0(2)	5031.5(2)	5214.9(2)	18.10(5)
Cl1	6622.1(6)	5231.9(5)	4272.5(4)	24.04(19)
P1	7964.6(6)	6431.4(6)	5208.7(5)	19.37(19)
P2	7283.2(6)	3613.6(6)	5205.1(5)	20.85(19)
O1	9257.8(19)	4626.6(19)	4913.5(13)	30.5(6)
N1	11137(3)	4647(4)	5972(2)	64.7(17)
C1	7148(3)	7027(2)	5653(2)	23.4(8)
C2	6351(3)	6684(3)	5830(2)	26.6(9)
C3	5734(3)	7131(3)	6196(2)	29.7(9)
C4	5907(3)	7933(3)	6385(2)	32.0(10)
C5	6694(3)	8286(3)	6209(2)	32.7(10)
C6	7317(3)	7846(2)	5848(2)	27.7(8)
C7	8098(2)	6772(2)	4318(2)	22.1(8)
C8	8473(3)	6244(3)	3828(2)	28.2(9)
C9	8636(3)	6515(3)	3153(2)	32.0(9)
C10	8428(3)	7308(3)	2958(2)	34.2(10)
C11	8047(3)	7839(3)	3439(2)	33.3(10)
C12	7880(3)	7575(3)	4115(2)	27.5(9)
C13	8982(3)	6666(2)	5634(2)	24.2(8)
C14	9015(3)	6751(3)	6368(2)	26.5(9)
C15	9787(3)	6922(3)	6696(2)	34.4(10)
C16	10547(3)	7000(4)	6306(3)	41.6(12)
C17	10527(3)	6903(3)	5587(2)	40.5(11)
C18	9747(3)	6737(3)	5257(2)	30.8(9)
C19	6248(3)	3418(2)	5612(2)	25.6(9)
C20	6019(3)	2615(3)	5816(2)	36.7(11)
C21	5233(3)	2474(3)	6137(3)	44.3(13)
C22	4672(3)	3111(4)	6274(2)	42.6(13)
C23	4885(3)	3897(3)	6079(2)	37.4(11)
C24	5673(3)	4051(3)	5751(2)	28.5(9)
C25	7271(3)	3266(2)	4310(2)	26.1(8)
C26	6658(3)	2679(3)	4085(2)	36.7(10)
C27	6693(4)	2392(3)	3401(3)	45.9(13)
C28	7318(3)	2678(3)	2948(2)	40.9(12)
C29	7923(3)	3261(3)	3160(2)	35.3(11)
C30	7902(3)	3556(3)	3846(2)	28.3(9)
C31	8055(3)	2966(2)	5654(2)	26.9(8)
C32	8039(3)	2928(3)	6387(2)	31.8(10)
C33	8642(3)	2438(3)	6734(2)	41.1(12)
C34	9261(3)	1993(3)	6361(3)	41.1(12)
C35	9273(3)	2034(3)	5641(3)	39.9(11)
C36	8676(3)	2524(3)	5288(2)	31.4(9)
C37	8272(2)	4885(2)	6113.3(18)	21.9(7)
C38	7793(2)	4975(3)	6732.9(18)	26.6(7)
C39	8199(3)	4861(3)	7380(2)	33.9(10)
C40	9071(3)	4653(3)	7406(2)	38.9(11)
C41	9550(3)	4572(3)	6791(2)	31.9(9)
C42	9169(3)	4701(2)	6139(2)	24.2(8)
C43	9656(3)	4639(3)	5464(2)	25.5(8)
C44	10646(3)	4602(3)	5407(2)	25.3(8)
C45	10988(3)	4537(3)	4750(2)	39.6(11)
C46	11885(3)	4496(4)	4669(3)	44.4(12)
C47	12397(3)	4505(3)	5247(3)	40.5(10)
C48	12005(4)	4593(6)	5874(3)	82(3)
P3	4683.9(8)	5454.2(7)	7590.6(6)	31.2(3)
F1	5511.1(19)	5197.0(18)	7138.4(14)	42.9(7)
F2	5253(2)	5382.0(19)	8292.1(13)	45.4(7)

Atom	x	y	z	U_{eq}
F3	3864(2)	5739(2)	8046.0(16)	53.4(8)
F4	4124(2)	5538.3(19)	6891.3(14)	50.1(8)
F5	4967(2)	6393.0(17)	7526.2(15)	52.0(8)
F6	4401(2)	4521.2(19)	7654.1(17)	52.4(9)
O2	8871(3)	-369(2)	6223.9(18)	47.6(9)
O3	8878(3)	-1154(2)	7173.5(19)	55.5(11)
C49	9338(4)	-836(3)	5105(3)	46.8(12)
C50	9414(3)	-972(3)	5873(3)	38.5(11)
C51	8661(3)	-525(3)	6890(3)	39.3(11)
C52	8163(4)	153(4)	7219(3)	60.9(17)

Table 21: Anisotropic Displacement Parameters (×10[4]) **[Au(C^CON)Cl(triphenylphosphine)$_2$]$^+$**. The anisotropic displacement factor exponent takes the form: $-2\pi^2[h^2a^{*2} \times U_{11} + ... +2hka^* \times b^* \times U_{12}]$

Atom	U_{11}	U_{22}	U_{33}	U_{23}	U_{13}	U_{12}
Au1	18.57(8)	21.39(8)	14.32(8)	0.37(6)	0.13(5)	0.10(6)
Cl1	23.9(4)	28.4(5)	19.8(4)	2.9(3)	-4.0(3)	2.0(3)
P1	19.7(5)	21.0(4)	17.4(4)	0.6(4)	0.7(4)	-1.8(3)
P2	24.7(5)	20.3(4)	17.6(4)	-0.4(4)	-0.8(4)	0.7(4)
O1	26.3(14)	50.1(17)	15.2(13)	-2.3(12)	-2.4(11)	4.2(13)
N1	25(2)	139(5)	30(2)	2(3)	-3.3(17)	9(2)
C1	22.6(19)	26.0(18)	21.5(19)	-0.1(16)	-0.3(16)	1.2(15)
C2	27(2)	27(2)	26(2)	1.6(16)	1.9(17)	-1.3(17)
C3	24(2)	35(2)	29(2)	-0.9(18)	5.7(18)	2.0(18)
C4	30(2)	41(2)	25(2)	-7.2(19)	-1.3(18)	11.3(19)
C5	34(2)	28(2)	37(2)	-10.4(18)	-7.4(19)	4.2(18)
C6	27(2)	26.2(18)	29(2)	-5.2(15)	-3.1(17)	-2.8(17)
C7	19.4(18)	27.1(19)	19.9(19)	3.7(16)	-2.2(16)	-4.7(15)
C8	27(2)	32(2)	26(2)	-0.5(17)	1.6(17)	-3.8(18)
C9	27(2)	47(3)	22(2)	-0.1(19)	0.7(17)	-7.2(19)
C10	26(2)	55(3)	22(2)	13(2)	-0.7(17)	-11(2)
C11	26(2)	38(2)	36(2)	18.3(19)	-2.6(19)	-5.1(19)
C12	25(2)	29(2)	29(2)	4.0(16)	-0.9(16)	-1.0(17)
C13	21.0(19)	25.5(19)	26(2)	1.0(17)	-0.7(17)	-0.7(15)
C14	24(2)	33(2)	22(2)	-2.1(17)	2.9(16)	-2.9(17)
C15	33(2)	48(3)	22(2)	-0.1(19)	-1.4(18)	-1(2)
C16	24(2)	66(3)	35(3)	-2(2)	-8.3(19)	-6(2)
C17	23(2)	69(3)	30(2)	4(2)	1.7(19)	-6(2)
C18	27(2)	48(2)	17.2(19)	-0.8(18)	-0.5(17)	-4.5(18)
C19	27(2)	27(2)	23(2)	2.2(17)	-1.3(18)	-5.3(16)
C20	41(3)	27(2)	42(3)	10.3(19)	-9(2)	-6.1(19)
C21	48(3)	46(3)	39(3)	23(2)	-10(2)	-22(2)
C22	37(3)	64(3)	26(2)	4(2)	2(2)	-25(3)
C23	32(2)	46(3)	35(2)	-15(2)	8.8(19)	-13(2)
C24	32(2)	26(2)	27(2)	-5.2(17)	3.0(17)	-7.1(17)
C25	30(2)	28.0(19)	20.3(19)	-3.0(16)	-6.3(17)	6.8(17)
C26	37(3)	40(3)	33(2)	-14(2)	-3(2)	-1(2)
C27	45(3)	53(3)	39(3)	-27(2)	-11(2)	5(2)
C28	46(3)	51(3)	25(2)	-18(2)	-13(2)	19(2)
C29	46(3)	40(2)	20(2)	-1.1(18)	-1.7(19)	19(2)
C30	32(2)	26.7(19)	26(2)	0.4(16)	-1.5(17)	6.4(17)
C31	29(2)	26(2)	25(2)	-1.3(17)	-3.9(18)	-0.2(16)
C32	38(2)	34(2)	24(2)	4.5(18)	0.6(18)	8.8(19)
C33	51(3)	49(3)	23(2)	6(2)	-3(2)	14(2)
C34	41(3)	46(3)	36(3)	4(2)	-8(2)	17(2)
C35	38(3)	48(3)	34(2)	-2(2)	-3(2)	15(2)
C36	34(2)	39(2)	21(2)	-2.6(18)	-0.6(18)	6.8(18)
C37	28.1(18)	22.2(18)	15.3(15)	0.1(15)	0.6(14)	-0.1(17)
C38	27.7(17)	33.9(19)	18.1(17)	-0.4(17)	1.8(14)	3(2)
C39	32(2)	53(3)	17.1(17)	-1.2(19)	2.5(15)	0(2)
C40	36(2)	64(3)	16.7(19)	2(2)	-4.8(18)	7(2)
C41	27(2)	47(3)	22(2)	0.8(19)	-2.3(17)	5.5(19)
C42	26(2)	29(2)	17.9(18)	0.4(15)	-1.6(15)	1.5(15)
C43	23(2)	30(2)	23(2)	-0.3(15)	1.4(16)	3.8(16)
C44	23(2)	27.5(19)	25(2)	0.4(15)	-0.6(15)	2.7(16)
C45	28(2)	67(3)	24(2)	-6(2)	-0.6(19)	-1(2)
C46	29(2)	72(3)	32(2)	-6(2)	7.1(19)	-2(2)
C47	24(2)	54(3)	44(3)	5(2)	3(2)	0.4(19)
C48	25(3)	179(8)	41(3)	7(4)	-7(2)	8(4)
P3	38.1(7)	33.3(6)	22.2(5)	2.6(4)	1.3(5)	4.8(5)
F1	43.8(15)	48.9(17)	35.9(13)	-8.8(12)	7.5(11)	2.8(13)
F2	49.8(17)	62.1(18)	24.3(12)	3.3(12)	-5.4(12)	6.1(14)

Atom	U_{11}	U_{22}	U_{33}	U_{23}	U_{13}	U_{12}
F3	41.5(16)	78(2)	40.5(17)	-7.6(15)	7.9(14)	8.5(16)
F4	68(2)	49.8(17)	32.2(14)	-6.2(13)	-15.3(14)	23.8(16)
F5	87(2)	32.1(14)	37.1(14)	-2.0(13)	6.7(16)	-0.7(15)
F6	63(2)	37.6(16)	56.3(19)	13.8(13)	-1.2(16)	-12.0(15)
O2	57(2)	45.7(19)	40.3(18)	11.3(15)	7.8(17)	7.0(17)
O3	81(3)	46(2)	39.3(19)	10.8(17)	-11(2)	-10(2)
C49	42(3)	52(3)	47(3)	-3(2)	5(2)	-3(2)
C50	30(2)	39(2)	47(3)	-2(2)	4(2)	-6(2)
C51	34(2)	48(3)	35(2)	8(2)	-6(2)	-8(2)
C52	61(3)	88(5)	34(3)	1(3)	-4(2)	22(4)

Table 22: Bond Lengths in Å for **[Au(C^{CO}N)Cl(triphenylphosphine)$_2$]$^+$**.

Atom	Atom	Length/Å
Au1	Cl1	2.3592(8)
Au1	P1	2.3569(9)
Au1	P2	2.3628(9)
Au1	C37	2.027(3)
P1	C1	1.800(4)
P1	C7	1.804(4)
P1	C13	1.803(4)
P2	C19	1.798(4)
P2	C25	1.803(4)
P2	C31	1.805(4)
O1	C43	1.218(5)
N1	C44	1.321(6)
N1	C48	1.350(7)
C1	C2	1.387(6)
C1	C6	1.412(5)
C2	C3	1.385(6)
C3	C4	1.385(6)
C4	C5	1.381(7)
C5	C6	1.382(6)
C7	C8	1.397(6)
C7	C12	1.408(6)
C8	C9	1.387(6)
C9	C10	1.385(7)
C10	C11	1.393(7)
C11	C12	1.388(6)
C13	C14	1.411(6)
C13	C18	1.384(6)
C14	C15	1.370(6)
C15	C16	1.390(7)
C16	C17	1.385(7)
C17	C18	1.380(6)
C19	C20	1.411(6)
C19	C24	1.385(6)
C20	C21	1.374(7)
C21	C22	1.375(8)
C22	C23	1.375(7)
C23	C24	1.387(6)
C25	C26	1.410(6)
C25	C30	1.397(6)
C26	C27	1.391(7)
C27	C28	1.374(8)
C28	C29	1.390(7)
C29	C30	1.400(6)
C31	C32	1.403(6)
C31	C36	1.386(6)
C32	C33	1.392(6)
C33	C34	1.393(7)
C34	C35	1.379(7)
C35	C36	1.391(6)
C37	C38	1.403(5)
C37	C42	1.410(5)
C38	C39	1.398(5)
C39	C40	1.383(7)
C40	C41	1.395(6)
C41	C42	1.394(5)
C42	C43	1.495(5)
C43	C44	1.525(6)
C44	C45	1.366(6)
C45	C46	1.388(7)
C46	C47	1.357(7)
C47	C48	1.350(7)
P3	F1	1.593(3)
P3	F2	1.606(3)
P3	F3	1.600(3)
P3	F4	1.596(3)
P3	F5	1.598(3)
P3	F6	1.589(3)
O2	C50	1.454(6)
O2	C51	1.338(6)
O3	C51	1.209(6)
C49	C50	1.489(7)
C51	C52	1.487(8)

Table 23: Bond Angles in ° for **$[Au(C^{CO}N)Cl(triphenylphosphine)_2]^+$**.

Atom	Atom	Atom	Angle/°
Cl1	Au1	P2	90.24(3)
P1	Au1	Cl1	90.89(3)
P1	Au1	P2	177.27(3)
C37	Au1	Cl1	171.83(10)
C37	Au1	P1	89.58(11)
C37	Au1	P2	89.65(11)
C1	P1	Au1	110.55(13)
C1	P1	C7	111.05(19)
C1	P1	C13	106.01(19)
C7	P1	Au1	109.34(13)
C13	P1	Au1	114.52(13)
C13	P1	C7	105.23(18)
C19	P2	Au1	110.30(14)
C19	P2	C25	110.2(2)
C19	P2	C31	105.7(2)
C25	P2	Au1	108.55(13)
C25	P2	C31	105.92(19)
C31	P2	Au1	116.05(14)
C44	N1	C48	116.5(5)
C2	C1	P1	120.8(3)
C2	C1	C6	118.6(4)
C6	C1	P1	120.5(3)
C3	C2	C1	121.0(4)
C2	C3	C4	119.9(4)
C5	C4	C3	120.0(4)
C4	C5	C6	120.7(4)
C5	C6	C1	119.9(4)
C8	C7	P1	119.4(3)
C8	C7	C12	119.2(4)
C12	C7	P1	121.3(3)
C9	C8	C7	120.2(4)
C10	C9	C8	120.4(4)
C9	C10	C11	120.1(4)
C12	C11	C10	119.9(4)
C11	C12	C7	120.1(4)
C14	C13	P1	120.1(3)
C18	C13	P1	121.2(3)
C18	C13	C14	118.6(4)
C15	C14	C13	120.4(4)
C14	C15	C16	120.0(4)
C17	C16	C15	120.2(4)
C18	C17	C16	119.7(4)
C17	C18	C13	121.0(4)
C20	C19	P2	120.3(3)
C24	C19	P2	120.9(3)
C24	C19	C20	118.7(4)
C21	C20	C19	119.9(5)
C20	C21	C22	120.6(4)
C23	C22	C21	120.3(4)
C22	C23	C24	119.9(5)
C19	C24	C23	120.5(4)
C26	C25	P2	120.8(3)
C30	C25	P2	119.2(3)
C30	C25	C26	119.9(4)
C27	C26	C25	119.4(5)
C28	C27	C26	120.3(5)
C27	C28	C29	121.1(4)
C28	C29	C30	119.6(5)
C25	C30	C29	119.7(4)
C32	C31	P2	119.3(3)
C36	C31	P2	121.1(3)
C36	C31	C32	119.5(4)
C33	C32	C31	119.4(4)
C32	C33	C34	120.6(4)
C35	C34	C33	119.7(4)
C34	C35	C36	120.2(4)
C31	C36	C35	120.6(4)
C38	C37	Au1	115.7(3)
C38	C37	C42	120.3(3)
C42	C37	Au1	124.0(3)
C39	C38	C37	120.0(3)
C40	C39	C38	119.8(4)
C39	C40	C41	120.2(4)
C42	C41	C40	121.3(4)
C37	C42	C43	118.2(3)
C41	C42	C37	118.3(4)
C41	C42	C43	123.5(4)
O1	C43	C42	119.8(4)
O1	C43	C44	115.9(3)
C42	C43	C44	124.3(3)
N1	C44	C43	120.5(4)
N1	C44	C45	122.5(4)
C45	C44	C43	117.0(4)
C44	C45	C46	119.3(4)
C47	C46	C45	118.9(4)
C48	C47	C46	117.8(4)
N1	C48	C47	124.8(5)
F1	P3	F2	90.02(16)
F1	P3	F3	178.34(19)
F1	P3	F4	89.82(17)
F1	P3	F5	89.65(17)
F3	P3	F2	89.70(16)
F4	P3	F2	179.2(2)
F4	P3	F3	90.45(17)
F4	P3	F5	89.96(18)
F5	P3	F2	89.24(18)
F5	P3	F3	88.70(18)
F6	P3	F1	90.40(18)
F6	P3	F2	90.85(18)
F6	P3	F3	91.24(19)
F6	P3	F4	89.95(19)
F6	P3	F5	179.9(2)
C51	O2	C50	116.7(4)
O2	C50	C49	108.0(4)
O2	C51	C52	112.7(4)
O3	C51	O2	121.5(5)
O3	C51	C52	125.8(5)

Table 24: Torsion Angles in ° for **[Au(C^{CO}N)Cl(triphenylphosphine)$_2$]$^+$**.

Atom	Atom	Atom	Atom	Angle/°
Au1	P1	C1	C2	-11.8(4)
Au1	P1	C1	C6	166.3(3)
Au1	P1	C7	C8	-37.0(3)
Au1	P1	C7	C12	146.8(3)
Au1	P1	C13	C14	-82.1(3)
Au1	P1	C13	C18	95.6(3)
Au1	P2	C19	C20	-165.0(3)
Au1	P2	C19	C24	13.0(4)
Au1	P2	C25	C26	-141.6(3)
Au1	P2	C25	C30	41.3(3)
Au1	P2	C31	C32	77.0(4)
Au1	P2	C31	C36	-101.3(3)
Au1	C37	C38	C39	178.1(3)
Au1	C37	C42	C41	-176.5(3)
Au1	C37	C42	C43	2.2(5)
P1	C1	C2	C3	177.5(3)
P1	C1	C6	C5	-177.8(3)
P1	C7	C8	C9	-175.5(3)
P1	C7	C12	C11	175.4(3)
P1	C13	C14	C15	179.5(3)
P1	C13	C18	C17	-179.0(4)
P2	C19	C20	C21	178.9(4)
P2	C19	C24	C23	-178.5(3)
P2	C25	C26	C27	-176.7(4)
P2	C25	C30	C29	177.0(3)
P2	C31	C32	C33	-179.1(4)
P2	C31	C36	C35	179.3(4)
O1	C43	C44	N1	177.2(5)
O1	C43	C44	C45	-1.7(6)
N1	C44	C45	C46	1.6(8)
C1	P1	C7	C8	-159.3(3)
C1	P1	C7	C12	24.5(4)
C1	P1	C13	C14	40.1(4)
C1	P1	C13	C18	-142.2(3)
C1	C2	C3	C4	0.5(6)
C2	C1	C6	C5	0.3(6)
C2	C3	C4	C5	0.0(7)
C3	C4	C5	C6	-0.3(7)
C4	C5	C6	C1	0.2(6)
C6	C1	C2	C3	-0.6(6)
C7	P1	C1	C2	109.8(3)
C7	P1	C1	C6	-72.2(4)
C7	P1	C13	C14	157.8(3)
C7	P1	C13	C18	-24.5(4)
C7	C8	C9	C10	-0.1(6)
C8	C7	C12	C11	-0.9(6)
C8	C9	C10	C11	-0.5(7)
C9	C10	C11	C12	0.4(7)
C10	C11	C12	C7	0.3(6)
C12	C7	C8	C9	0.8(6)
C13	P1	C1	C2	-136.5(3)
C13	P1	C1	C6	41.6(4)
C13	P1	C7	C8	86.5(3)
C13	P1	C7	C12	-89.7(3)
C13	C14	C15	C16	-1.1(7)
C14	C13	C18	C17	-1.3(7)
C14	C15	C16	C17	-0.1(8)
C15	C16	C17	C18	0.6(9)

Atom	Atom	Atom	Atom	Angle/°
C16	C17	C18	C13	0.1(8)
C18	C13	C14	C15	1.8(7)
C19	P2	C25	C26	-20.7(4)
C19	P2	C25	C30	162.2(3)
C19	P2	C31	C32	-45.5(4)
C19	P2	C31	C36	136.1(4)
C19	C20	C21	C22	-1.2(7)
C20	C19	C24	C23	-0.4(7)
C20	C21	C22	C23	1.2(7)
C21	C22	C23	C24	-0.8(7)
C22	C23	C24	C19	0.4(7)
C24	C19	C20	C21	0.8(7)
C25	P2	C19	C20	75.2(4)
C25	P2	C19	C24	-106.8(4)
C25	P2	C31	C32	-162.5(4)
C25	P2	C31	C36	19.2(4)
C25	C26	C27	C28	0.0(8)
C26	C25	C30	C29	-0.1(6)
C26	C27	C28	C29	-0.5(8)
C27	C28	C29	C30	0.8(7)
C28	C29	C30	C25	-0.5(6)
C30	C25	C26	C27	0.3(7)
C31	P2	C19	C20	-38.8(4)
C31	P2	C19	C24	139.2(4)
C31	P2	C25	C26	93.1(4)
C31	P2	C25	C30	-83.9(3)
C31	C32	C33	C34	0.5(8)
C32	C31	C36	C35	1.0(7)
C32	C33	C34	C35	-0.5(8)
C33	C34	C35	C36	0.7(9)
C34	C35	C36	C31	-1.0(8)
C36	C31	C32	C33	-0.7(7)
C37	C38	C39	C40	-0.5(7)
C37	C42	C43	O1	-10.7(6)
C37	C42	C43	C44	168.3(4)
C38	C37	C42	C41	3.3(6)
C38	C37	C42	C43	-178.0(4)
C38	C39	C40	C41	1.3(8)
C39	C40	C41	C42	0.3(8)
C40	C41	C42	C37	-2.6(7)
C40	C41	C42	C43	178.8(4)
C41	C42	C43	O1	168.0(4)
C41	C42	C43	C44	-13.1(6)
C42	C37	C38	C39	-1.8(6)
C42	C43	C44	N1	-1.8(7)
C42	C43	C44	C45	179.3(4)
C43	C44	C45	C46	-179.5(5)
C44	N1	C48	C47	0.5(12)
C44	C45	C46	C47	1.3(8)
C45	C46	C47	C48	-3.0(9)
C46	C47	C48	N1	2.3(12)
C48	N1	C44	C43	178.7(6)
C48	N1	C44	C45	-2.4(9)
C50	O2	C51	O3	-3.4(7)
C50	O2	C51	C52	175.6(4)
C51	O2	C50	C49	162.1(4)

Table 25: Hydrogen Fractional Atomic Coordinates (×10^4) and Equivalent Isotropic Displacement Parameters (Å^2×10^3) for **[Au(C^{CO}N)Cl(triphenylphosphine)$_2$]$^+$**. U_{eq} is defined as 1/3 of the trace of the orthogonalised U_{ij}.

**Atom**	**x**	**y**	**z**	U_{eq}
H2	6229	6147	5702	32
H3	5205	6892	6314	36
H4	5494	8234	6631	38
H5	6807	8826	6336	39
H6	7846	8088	5732	33
H8	8613	5710	3954	34
H9	8886	6161	2829	38
H10	8544	7486	2506	41
H11	7905	8371	3306	40
H12	7623	7929	4435	33
H14	8510	6690	6631	32
H15	9803	6987	7178	41
H16	11071	7117	6529	50
H17	11036	6950	5328	49
H18	9736	6671	4774	37
H20	6399	2183	5732	44
H21	5079	1944	6263	53
H22	4147	3010	6500	51
H23	4501	4325	6168	45
H24	5817	4583	5623	34
H26	6235	2486	4391	44
H27	6291	2005	3249	55
H28	7338	2477	2494	49
H29	8338	3455	2846	42
H30	8307	3944	3993	34
H32	7629	3227	6638	38
H33	8631	2408	7219	49
H34	9665	1671	6597	49
H35	9682	1733	5390	48
H36	8694	2554	4803	38
H38	7206	5111	6713	32
H39	7883	4926	7791	41
H40	9338	4566	7836	47
H41	10136	4429	6816	38
H45	10625	4521	4361	47
H46	12131	4462	4225	53
H47	12999	4452	5213	49
H48	12360	4619	6268	98
H49A	9711	-1213	4863	70
H49B	9509	-285	4996	70
H49C	8746	-922	4962	70
H50A	9223	-1521	5991	46
H50B	10016	-911	6019	46
H52A	8136	610	6904	91
H52B	8447	318	7644	91
H52C	7583	-30	7324	91
